# Outcomes after perioperative SARS-CoV-2 infection in patients with proximal femoral fractures: an international cohort study

**DOI:** 10.1136/bmjopen-2021-050830

**Published:** 2021-11-30

**Authors:** COVIDSurg Collaborative

**Keywords:** COVID-19, hip, trauma management

## Abstract

**Objectives:**

Studies have demonstrated high rates of mortality in people with proximal femoral fracture and SARS-CoV-2, but there is limited published data on the factors that influence mortality for clinicians to make informed treatment decisions. This study aims to report the 30-day mortality associated with perioperative infection of patients undergoing surgery for proximal femoral fractures and to examine the factors that influence mortality in a multivariate analysis.

**Setting:**

Prospective, international, multicentre, observational cohort study.

**Participants:**

Patients undergoing any operation for a proximal femoral fracture from 1 February to 30 April 2020 and with perioperative SARS-CoV-2 infection (either 7 days prior or 30-day postoperative).

**Primary outcome:**

30-day mortality. Multivariate modelling was performed to identify factors associated with 30-day mortality.

**Results:**

This study reports included 1063 patients from 174 hospitals in 19 countries. Overall 30-day mortality was 29.4% (313/1063). In an adjusted model, 30-day mortality was associated with male gender (OR 2.29, 95% CI 1.68 to 3.13, p<0.001), age >80 years (OR 1.60, 95% CI 1.1 to 2.31, p=0.013), preoperative diagnosis of dementia (OR 1.57, 95% CI 1.15 to 2.16, p=0.005), kidney disease (OR 1.73, 95% CI 1.18 to 2.55, p=0.005) and congestive heart failure (OR 1.62, 95% CI 1.06 to 2.48, p=0.025). Mortality at 30 days was lower in patients with a preoperative diagnosis of SARS-CoV-2 (OR 0.6, 95% CI 0.6 (0.42 to 0.85), p=0.004). There was no difference in mortality in patients with an increase to delay in surgery (p=0.220) or type of anaesthetic given (p=0.787).

**Conclusions:**

Patients undergoing surgery for a proximal femoral fracture with a perioperative infection of SARS-CoV-2 have a high rate of mortality. This study would support the need for providing these patients with individualised medical and anaesthetic care, including medical optimisation before theatre. Careful preoperative counselling is needed for those with a proximal femoral fracture and SARS-CoV-2, especially those in the highest risk groups.

**Trial registration number:**

NCT04323644

**Strengths and limitations of this study**

- This is a large, international, multicentre cohort study from which the results are generalisable across populations in other countries.
- This study described specific risk factors for mortality, which patients and those who care for them should use to make informed decisions regarding care.
- There is not control arm to assess contemporaneous patients with undergoing an operation for proximal femoral fractures without SARS-CoV-2 infection during the height of the pandemic. However with high-quality data present prepandemic strongly suggests a substantial increase in mortality.

## Background

The rapid worldwide spread of COVID-19, caused by the SARS-CoV-2 has had a severe effect on the elderly and frail population. A fracture of the proximal femur (neck of femur fracture) is a critical event in the elderly, frail population, with a high rate of death despite medical and surgical intervention.^1^ Since 2007, there has been a steady improvement in mortality after a proximal femoral fracture with 6.1% of patients dying within 30 days of injury in the UK in 2018.^2^ However, the emergence of COVID-19 presents a new and unquantified risk to this particularly vulnerable group.

Proximal femur fractures represent a large international burden with incidence between 43 and 920 per 100 000 population.^3^ As most fractures of the proximal femur happen as a result of trips or falls in the home, people have continued to present with this injury despite social restrictions.^4 5^ These patients typically have multiple comorbidities and frailty is common.^1^ Resultantly, they are particularly vulnerable to pulmonary complications.^1 6^ It is widely accepted that elderly patients with existing comorbidities are at higher risks of critical illness and mortality due to COVID-19, potentially due to a higher preponderance to release proinflammatory cytokines that result in severe disease.^7–9^

Clinicians have been swift to respond to this pandemic with large reorganisation of service provision.^10 11^ In response to this, the COVIDSurg collaborative (www.globalsurg.org/covidsurg) has collected an international, large volume dataset to inform the global community of the safety of surgery in patients with perioperative SARS-CoV-2 infection. The first report has demonstrated a 30-day mortality of 23.8% across patients undergoing any type of surgery.^12^ Data published so far have reported a high mortality rate in a small cohort of patients with proximal femoral fractures positive for SARS-CoV-2 infection, with a maximum cohort size of 114 patients (range 10–114 patients).^13–20^ However, few reports have the sample size sufficient to explore the factors that influence outcome. Furthermore, large-scale data are required to explore preoperative and operative variables that influence outcomes in order to inform the clinical decision-making processes.

### Aims

The primary aim of this study is to determine the mortality rate observed in patients undergoing surgery for proximal femoral fracture with perioperative SARS-CoV-2 infection. Secondarily, we aim to explore the patient and treatment factors associated with these outcomes.

## Methods

### Setting

This is an international, multicentre cohort study including consecutive patients who underwent surgery for proximal femoral fracture from 1 February 2020 to 3 April 2020. This study is a preplanned sub-analysis of a larger, ongoing study designed to assess outcomes following all surgery for patients with perioperative SARS-CoV-2 infection.^12^

The COVIDSurg collaborative is an international, multicentre, multidisciplinary team with individual collaborators collecting data locally, which is collated centrally. The collaborative methodology, which is well described and validated, was used for this project.^21^

### Inclusion criteria

Participating hospitals included consecutive patients undergoing surgery for proximal femoral fractures that had SARS-CoV-2 infection diagnosed (laboratory, clinical or radiologically) either 7 days preoperatively or up to 30 days postoperatively. For those diagnosed preoperative, this represents the timeframe where the majority of patients still active disease.^22^ For those patients who underwent multiple procedures, the procedure closest to the time of confirmation of SARS-CoV-2 infection was defined as the index procedure.

Patients received laboratory confirmation of SARS-CoV-2 using quantitative reverse transcription Polymerase Chain Reseaction (qRT-PCR). As qRT-PCR is not available in all participating hospitals, patients were included if their diagnosis was made by clinical or radiological findings. Clinical diagnosis was made in patients presenting with symptoms and a clinical pattern of COVID-19. These included cough, fever and/or myalgia.^23^ Radiological diagnosis was made through CT scanning of the thorax according to local protocols. All patients who were included solely on clinical or radiological suspicion but had a subsequent negative qRT-PCR test were excluded from the database by individual collaborators.

### Diagnosis

This study includes all patients identified as having an operation for a proximal femoral fracture. The diagnosis was established pragmatically by the local site teams according to their assessment of the fracture. The reported data were screened by a central dedicated data cleaning team, with only confirmed proximal femoral fractures included in the cohort.

### Patient identification

Researchers at participating centres screened consecutive patients undergoing surgery to ensure all patients were identified. The study was initiated in some countries after their peak of infection, and therefore retrospective identification and data collection was permitted, as long as the data collection was consecutive at that site.

To reduce selection bias, a variety of written materials were distributed to site leads to highlight possible methods of identifying patients ensuring all eligible patients were included. Investigators were invited to social media groups and online teleconferences to troubleshoot recruitment issues, share learning and ensure consistent recruitment into the wider cohort.

### Outcome measures

The primary outcome measure was 30-day all-cause mortality, with the day of surgery defined as day zero. The secondary outcome measure was rate of pulmonary complications, which is a composite outcome defined previously from the Prevention of Respiratory Insufficiency after Surgical Management randomised controlled trial.^24 25^

Pulmonary complications were defined as pneumonia, acute respiratory distress syndrome and/or unexpected postoperative ventilation; these have been identified as the most frequent COVID-19 related pulmonary complications in medical patients.^23^ Unexpected postoperative ventilation was defined as either: (1) any episode of non-invasive ventilation, invasive ventilation or extracorporeal membrane oxygenation after initial extubation following surgery or (2) unexpected failure to extubate following surgery.^12^

### Data collection and quality assurance

Data were collected online using the Research Electronic Data Capture web application.^26^ Demographic variables recorded consisted of age, sex and American Society of Anesthesiologists (ASA) physical status classification. Age was collected as a categorical variable by deciles of age. ASA at the time of surgery was dichotomised to: (1) grades 1–2 and (2) grades 3–5 for the purpose of analysis, time to surgery to (1) under 24 hours, (2) 24–48 hours and (3) over 48 hours and surgery to (1) hemiarthroplasty, (2) total hip replacement, (3) dynamic hip screw, (4) cannulated screws and (5) intramedullary nail. The timing of SARS-CoV-2 diagnosis was recorded as either preoperative or postoperative.

Before data were entered into analysis, site principle investigators were required to confirm all consecutive eligible cases had been completed and uploaded. Where diagnosis was unclear, authors were contacted for clarification.

### Statistical analysis

The study was reported according to Strengthening the Reporting of Observational Studies in Epidemiology guidelines.^27^ Proportions are expressed with 95% CIs, and the mean and 95% CIs were used where data were assumed to be approximately normally distributed. Fisher’s exact test was used for categorical data. Non-parametric data was summarised with the median and IQRs. Statistical significance was assessed at the 5% level.

The risk of death at 30 days was chosen as the primary outcome for the study. Mixed-effects logistic regression analysis was used to assess the strength and significance of associations between a number of explanatory variables and death within 30 days. Random effects were included in the mixed-effects model to account for the hierarchical structure of the data (individual hospital effects are naturally nested within country effects), and fixed effects were included to adjust for a range of preoperative variables that may influence mortality in this population and relevant factors related to the injury or treatment (eg, type of operation, time from admission to operation and type of anaesthetic). An additional analysis of the same factors was undertaken using the same model structure for the secondary outcome of pulmonary complications. This was an exploratory analysis with the significance level set at 5%, with no specific adjustments made for model testing. All analyses were implemented in R (R Core Team (2020). R: A language and environment for statistical computing. R Foundation for Statistical Computing, Vienna, Austria. URL https://www.R-project.org).

### Patient and public involvement

Patients were not involved in the design, conduct or reporting of this study.

## Results

### Population

This study returned 30-day follow-up for 1063 patients with proximal femoral fractures. Data were collected in 174 hospitals from 19 countries (online supplemental table 1). Of these, 65.5% were female (696/1063). A percentage of 7.8% (83/1063) patients were <70 years old, 17.8% (189/1063) were between 70 and 79 years, 47.7% (507/1063) were between 80 and 89 years old and 26.7% (284/1063) were 90+ years old.

10.1136/bmjopen-2021-050830.supp1 Supplementary data

### Mortality

Overall 30-day mortality was 29.4% (313/1063). With each decile of age, mortality significantly increased, being highest in those patients >90 years old (38.7% (110/284), p=0.001).

**Figure 1:**
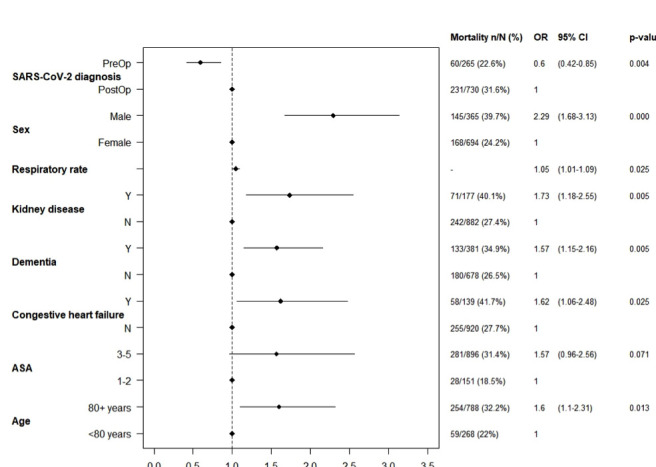
Mixed-effects logistic regression model for 30-day mortality. ASA, American Society of Anesthesiologists.

In an adjusted model (figure 1), 30-day mortality was associated with male gender (OR 2.29, 95% CI 1.68 to 3.13, p<0.001), age >80 years (OR 1.60, 95% CI 1.1 to 2.31, p=0.013), diagnosis of dementia (OR 1.57, 95% CI 1.15 to 2.16, p=0.005), chronic kidney disease (OR 1.73, 95% CI 1.18 to 2.55, p=0.005) and congestive heart failure (OR 1.62, 95% CI 1.06 to 2.48, p=0.025). Thirty-day mortality was lower in patients with a preoperative diagnosis of SARS-CoV-2 (OR 0.60, 95% CI 0.42 to 0.85, p=0.004). Non-adjusted values are presented in online supplemental table 2.

### Pulmonary complications

**Figure 2:**
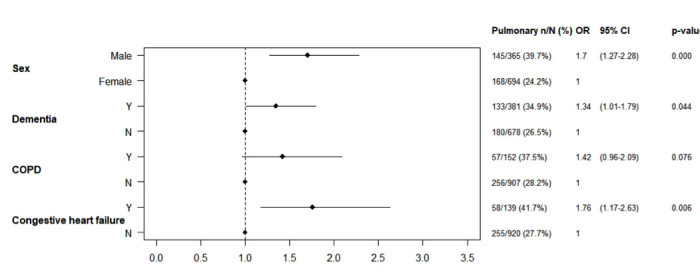
Mixed-effects logistic regression model for pulmonary complications. COPD, chronic obstructive pulmonary disorder.

In an adjusted model (figure 2), respiratory complications were associated with male gender (OR 1.7, 95% CI 1.27 to 2.28, p<0.001), diagnosis of dementia (OR 1.34, 95% CI 1.01 to 1.79, p=0.044) and congestive heart failure (OR 1.76, 95% CI 1.17 to 2.63, p=0.006). The presence of chronic obstructive pulmonary disorder dshowed no significant association (OR 1.42, 95% CI 0.96 to 2.09, p=0.076).

### Diagnosis

The majority of diagnosis of SARS-CoV-2 was made via PCR swab testing 93.3% (992/1063) (online supplemental tables 1 and 3), and there was no difference in mortality between those diagnosed clinically (p=0.668). The majority of patients 69% (733/1063) were diagnoses postoperatively.

### Preoperative variables

Preoperative symptoms (online supplemental table 4), including breathlessness, cough and fever (>38°C) were not significantly different in patients who were alive or dead at 30 days postoperatively. On examination of preoperative observations, a high respiratory rate was predictive of mortality (OR 1.73 95% CI 1.18 to 2.55, p=0.025) (figure 1). However, there was no significant difference in patient’s heart rate, systolic or diastolic blood pressure (online supplemental table 5 and figure 1) between those who were alive or dead at 30 days.

Those patients with ASA grade 3–5 had a significantly higher mortality of 31.4% (281/899) versus ASA of 1–2 of 18.5% (28/151), p=0.001.

### Procedures

The operations were carried out under a general anaesthetic in 49.6% (527/1063) of patients (online supplemental table 6). A percentage of 67.2 (714/1063) of patients did not require any preoperative oxygen therapy. In this cohort, 31.8% (338/1063) of patients had their operation within 24 hours of presentation to hospital, 21.1% (224/1063) had their operation between 24 and 47 hours and 19.2% (205/1063) of patients had their operation after 48 hours of presentation to hospital.

In this cohort, 45.1% (479/1063) of patients underwent haemiarthroplasty with a further 4.2% undergoing total hip replacement (45/1063). For patients who underwent fixation, 26% (276/1063) underwent dynamic hip screw fixation, 22.9% (243/1063) patients underwent intramedullary fixation, 0.5% (5/1063) underwent cannulated screw fixation, while a further 1.4% (15/1053) underwent internal fixation.

There was no difference in mortality between patients undergoing general and regional anaesthesia (29.9% (157/527) vs 29.0% (152/524), p=0.787). However, there was an increased mortality in those patients requiring preoperative oxygen therapy (34.3% (115/336) vs 27.2% (194/714), p=0.031).

There was no significant difference in mortality for patients with delayed operation. The highest mortality was for patients operated between 24 and 47 hours of admission (34.4% (77/224)) but was not significantly higher than less than those operated after 48 hours (p=0.220).

Mortality was highest in March (33.7%, 159/474) compared with April (27.0%, 150/558) and February (11.5%, 3/26), p=0.007 (online supplemental table 1).

## Discussion

The 30-day mortality rate for patients with a perioperative diagnosis of SARS-CoV-2 infection undergoing surgery for proximal femoral fracture is substantial. An overall rate of 29.4% compares with the reported 30-day mortality in the literature for proximal femoral fractures ranging between 3.5% and 6.8%.^2 28–32^ This rate is higher than found at the 1 year time point.^33^ Furthermore, elderly patients and those with medical comorbidities such as dementia, chronic kidney disease and congestive heart failure were associated with higher risk of 30-day mortality. Notably, patients with a preoperative diagnosis of SARS-CoV-2 infection had lower rates of 30-day mortality, likely reflecting early recognition and closer management of these patients. Findings from this study will be useful in guiding clinicians to identify high-risk patients that may warrant closer medical and surgical input during the COVID-19 pandemic.

Considering this high mortality, it is critical that patients who present without a diagnosis SARS-CoV-2 with proximal femoral fractures are protected from contracting SARS-CoV-2 in the perioperative period. A study by Kayani *et al*^17^ has suggested that half of infections in patients with proximal femoral fractures occur in hospital, as denoted by having negative preoperative samples. Similarly, a study by Hall *et al*^34^ has suggested nearly half of cases were due to nosocomial transmission. Within this study, 733 (69%) of infections were diagnosed postoperatively. This may infer that infections have been transferred in hospital, although due to incubation period of the virus, it is hard to know the proportion that contracted the virus prior to presentation or in hospital.^7 35^ Higher mortality was observed in people who had a postoperative diagnosis, which emphasises the critical importance of avoiding in-hospital transmission. Hospitals should consider implementation of careful infection control processes to minimise and prevent transmission of SARS-CoV-2 infection. Within the elective setting, the creation of COVID-19 free surgical pathways for elective patients has been shown to reduce infection and subsequent mortality^36–38^ and while only some of the principles are transferrable to the emergency setting, it demonstrates the value of meticulous infection control processes throughout the hospital stay. Furthermore, patients should be reinforced of methods to reduce risk of transmission in the community after discharge, including (but not limited to) social distancing, isolation and hygiene.

For those patients presenting with SARS-CoV-2 (either existing diagnosis or clinical findings suggestive of) and a proximal femoral fracture, it is important for data to be used as part of the informed consent process. In patients with multiple high-risk factors such as those who are more elderly, have respiratory and cardiac comorbidities, non-operative management may be considered following an appropriate discussion with the patient and/or their family. Every year in the UK, 2.5% of hip fractures are treated non-operatively.^39^ A study performed before the pandemic reported that the mortality within 30 days for conservatively treated patients was 31.3%.^40^ We do not know the mortality from non-operative management during the pandemic for patients with SARS-Cov-2, but the particularly high mortality associated with surgery in high-risk groups may change the balance of benefit and harm towards conservative treatment, and this should be considered.

The 30-day mortality of 29.4% identified within this study is comparable with published literature in the UK (range from 16.3% to 35.6%),^15–17 19 34 41^ Italy (18.75%),^14^ Spain (30.4%)^13^ and the USA (range from 35.3% to 56%).^18 20^ From a study within the UK, the authors also found a correlation between male sex and increased mortality (OR 2.69), which is similar to that demonstrated in this study (OR 2.29).^16^ Additionally, another UK study reported having more than three comorbidities as a risk factor for mortality.^17^ This study has specifically delineated a diagnosis of dementia, chronic kidney disease and congestive heart failure as being independent risk factors for mortality. In a study from USA, the authors found those patients who died were older with multiple comorbidities, and this was reflected in statistically significant higher ASA scores in comparison with their negative counterparts.^20^

This study found that there was no significant increase in mortality with delay to surgery. Current guidelines suggest early surgery should be undertaken,^42^ and this is associated with lower mortality.^43^ This would suggest that those patients at the highest risk of mortality can have medical optimisation, if appropriate, and will not result in a higher mortality from SARS-CoV-2 infection. This includes correction of concurrent medical issues often found in this population, examples of which include correction of acute renal failure, electrolyte disturbances and/or anticoagulation related issues. With regards to recovery from SARS-CoV-2 infection, it is important to consider that an increased risk of mortality for those undergoing surgery persists until 7 weeks after diagnosis.^44^ This risk reduces gradually after 2 weeks after diagnosis and should be considered.

Similarly, previous studies have found a higher rate of mortality in patients undergoing general versus regional anaesthesia for proximal femoral fractures.^45 46^ This study reports no difference between general and regional anaesthetic (29.9% vs 29.0%, p=0.787). While this was not the primary outcome of this study, this suggests that a positive test for SARS-CoV-2 should not have a large influence on anaesthetic decisions. This should be interpreted with caution in the light of this being an exploratory study. Out of all clinical features, respiratory rate at presentation was associated with higher mortality. Clinicians should focus on this as an important finding when counselling patients of their perioperative mortality.

This study has also found an increased mortality during the month of March 2020. This corresponds to the peak of caseload of infections internationally.^47 48^ Increased circulation of SARS-CoV-2 within countries has shown to increase mortality through higher viral loads.^47 49^ This study validates that surgical patients are particularly susceptible during surge of cases.

This is a large, varied cohort of patients undergoing surgery for a proximal femoral fractures with SARS-CoV-2 infection diagnosed perioperatively. This study was conducted in multiple centres, internationally, allowing it to be generalisable across populations in other countries.

### Strength and Limitations

This study was conducted in hospitals in the early to midphase of the pandemic where routine testing was not available in all participating centres. As such, to be pragmatic, patients were included if a clinical diagnosis was made by the treating physician. Protocols were not standardised for clinical diagnosis and were left the senior treating physician. Laboratory diagnosis was made by qRT-PCR, from which false-negative results may have excluded patients from analysis. Indeed, the sensitivity of qRT-PCR testing for has shown to be as low as 32% for throat swabs.^50^ However, in patients with negative results and high clinical suspicion of SARS-CoV-2 infection, multiple samples are often taken, including broncho-alveolar lavage. Thus, the number of patients excluded is expected to be low. While this study reports a higher mortality from postoperative diagnosis of SARS-CoV-2 infection, it is unclear whether the infection was contracted preoperatively or not, as has been discussed previously.

This study does not have a control arm, assessing contemporaneous patients with undergoing an operation for proximal femoral fractures without SARS-CoV-2 infection during the height of the pandemic. However, comparison with high-quality prepandemic data strongly suggests a substantial increase in mortality. Patients and those who care for them should consider this carefully when making decisions in this common and challenging clinical scenario.

## Conclusion

Patients undergoing surgery for a proximal femoral fracture with a peri-operative infection of SARS-CoV-2 have a high rate of mortality. The study would support the approach of providing these patients with individualised medical and anaesthetic care, including medical optimisation before theatre. It is imperative to prevent transmission of COVID-19 in the hospital setting. Careful preoperative counselling is needed for those with a proximal femoral fracture and SARS-CoV-2, especially those in the highest risk groups.

## Supplementary Material

Reviewer comments

Author's manuscript

## Data Availability

Data are available upon reasonable request. All data relevant to the study are included in the article or uploaded as supplementary information.
