# Supplementary material for: Outcomes after perioperative SARS-CoV-2 infection in patients with proximal femoral fractures: an international cohort study
Source: BMJ Open. 2021 Nov 30;11(11):e050830. doi: 10.1136/bmjopen-2021-050830 (PMC8634634; doi:10.1136/bmjopen-2021-050830)
Supplement: Supplementary data [file bmjopen-2021-050830supp001.pdf]

Supplementary Material

Table S1. Baseline characteristics (sex, age, ASA grade, cardiac risk, time of diagnosis, method of diagnosis and month study participant recruited) of the full study population (n = 1063), and died (n = 313) and alive (n =746) groups; p-values are for Fisher's exact tests comparing groups for each characteristic.

Characteristic	Full (n = 1063)	Alive (n = 746)	Died (n = 313)	Died (%)
*Sex (p-value = <0.001 ***)*				
Female	696 (65.5%)	526	168	24.2
Male	367 (34.5%)	220	145	39.7
Missing	0 (0.0%)	0	0	0.0
*Age (p-value = 0.001 **)*				
20-29 years	3 (0.3%)	3	0	0.0
30-39 years	2 (0.2%)	1	1	50.0
40-49 years	4 (0.4%)	3	1	25.0
50-59 years	24 (2.3%)	21	3	12.5
60-69 years	50 (4.7%)	39	11	22.0
70-79 years	189 (17.8%)	145	43	22.9
80-89 years	507 (47.7%)	360	144	28.6
90+ years	284 (26.7%)	174	110	38.7
Missing	0(0.0%)	0	0	0.0
*ASA (p-value = 0.001 **)*				
1-2	151 (14.2%)	123	28	18.5
3-5	899 (84.6%)	615	281	31.4
Missing	13(1.2%)	8	4	30.8

*Cardiac risk (p-value = <0.001 ***)*				
0	487 (45.8%)	372	114	23.5
1	349 (32.8%)	238	110	31.6
2	169 (15.9%)	106	61	36.5
3	44 (4.1%)	23	21	47.7
4	8 (0.8%)	5	3	37.5
5	1 (0.1%)	0	1	100.0
Missing	5 (0.47%)	2	3	60.0
*Time of diagnosis (p-value = 0.006 **)*				
Post-op	733 (69%)	499	231	31.6
Pre-op	266 (25%)	205	60	22.6
Missing	64 (6.0%)	42	22	34.4
Diagnosis (p-value = 0.668)				
Clinical	62 (5.8%)	42	20	32.3
Swab	992 (93.3%)	696	292	29.6
Missing				
*Month (p-value = 0.007 **)*				
February	26 (2.4%)	23	3	11.5
March	474 (44.6%)	313	159	33.7
April	558 (52.5%)	406	150	27.0
Missing	1(0.09%)	0	1	100.0

Table S2. Comorbidity data summaries by died (n = 313) and alive (n =746) groups. Data tabulated are counts, with estimated odds ratios (OR), with 95% confidence intervals, and p-values from Fisher's exact tests for each comorbidity.

Comorbidity	Alive	Died	OR	p-value

	(n = 746)	(n = 313)	(95% CI)		
	Y:N (%Y)	Y:N (%Y)			
Current smoker	34:712 (4.6%)	9:304 (2.9%)	0.62 (0.26, 1.34)	0.235	-
Asthma	53:693 (7.1%)	21:292 (6.7%)	0.94 (0.53, 1.62)	0.895	-
Current cancer diagnosis	57:689 (7.6%)	24:289 (7.7%)	1.00 (0.58, 1.68)	0.999	-
Chronic kidney disease (moderate/severe)	106:640 (14.2%)	71:242 (22.7%)	1.77 (1.25, 2.51)	0.001	**
Chronic obstructive pulmonary disease (COPD)	95:651 (12.7%)	57:256 (18.2%)	1.53 (1.05, 2.21)	0.027	*
Congenital abnormality - cardiac	4:742 (0.5%)	1:312 (0.3%)	0.60 (0.01, 6.04)	0.999	-
Congenital abnormality - non-cardiac	0:746 (0.0%)	4:309 (1.3%)	-	-	-
Congestive heart failure	81:665 (10.9%)	58:255 (18.5%)	1.87 (1.27, 2.73)	<0.001	***
Dementia	248:498 (33.2%)	133:180 (42.5%)	1.48 (1.12, 1.96)	0.005	**
Diabetes mellitus	142:604 (19.0%)	63:250 (20.1%)	1.07 (0.76, 1.51)	0.671	-
Hypertension	387:359 (51.9%)	186:127 (59.4%)	1.36 (1.03, 1.79)	0.026	*
Myocardial infarction or ischemic heart disease	103:643 (13.8%)	63:250 (20.1%)	1.57 (1.09, 2.25)	0.012	*
Peripheral vascular disease	34:712 (4.6%)	21:292 (6.7%)	1.51 (0.82, 2.72)	0.172	-
Stroke/ TIA	107:639 (14.3%)	57:256 (18.2%)	1.33 (0.92, 1.92)	0.114	-
Other (including other lung disease)	377:369 (50.5%)	158:155 (50.5%)	1.00 (0.76, 1.31)	0.999	-

Table S3. Diagnosis data summaries by died (n = 313) and alive (n =746) groups. Data tabulated are counts, with estimated odds ratios (OR), with 95% confidence intervals, and p-values from Fisher's exact tests for each diagnosis method.

Diagnosis	Alive (n = 746)	Died (n = 313)	OR (95% CI)	p-value	
	Y:N (%Y)	Y:N (%Y)			
Pre-op 4-7days					
CT thorax scan (negative for SARS-CoV-2)	10:736 (1.3%)	2:311 (0.6%)	0.47 (0.05, 2.24)	0.526	-

CT thorax scan (positive for SARS-CoV-2)	12:734 (1.6%)	0:313 (0%)	0.00 (0.00, 0.85)	0.023	*
Swab (negative for SARS-CoV-2)	17:729 (2.3%)	3:310 (1%)	0.42 (0.08, 1.45)	0.215	-
Swab (positive for SARS-CoV-2)	31:715 (4.2%)	8:305 (2.6%)	0.61 (0.24, 1.37)	0.283	-
Pre-op 1-3days					
CT thorax scan (negative for SARS-CoV-2)	8:738 (1.1%)	3:310 (1%)	0.89 (0.15, 3.75)	0.999	-
CT thorax scan (positive for SARS-CoV-2)	10:736 (1.3%)	3:310 (1%)	0.71 (0.13, 2.79)	0.765	-
Swab (negative for SARS-CoV-2)	41:705 (5.5%)	9:304 (2.9%)	0.51 (0.22, 1.08)	0.080	-
Swab (positive for SARS-CoV-2)	86:660 (11.5%)	15:298 (4.8%)	0.39 (0.20, 0.69)	<0.001	*
Pre-op surgery					
CT thorax scan (negative for SARS-CoV-2)	0:746 (0%)	0:313 (0%)	-	0.999	-
CT thorax scan (positive for SARS-CoV-2)	4:742 (0.5%)	0:313 (0%)	0.00 (0.00, 3.61)	0.326	-
Swab (negative for SARS-CoV-2)	13:733 (1.7%)	2:311 (0.6%)	0.36 (0.04, 1.62)	0.254	-
Swab (positive for SARS-CoV-2)	18:728 (2.4%)	7:306 (2.2%)	0.93 (0.32, 2.35)	1	-
Post-op Admission					
CT thorax scan (negative for SARS-CoV-2)	4:742 (0.5%)	1:312 (0.3%)	0.60 (0.01, 6.04)	0.999	-
CT thorax scan (positive for SARS-CoV-2)	8:738 (1.1%)	4:309 (1.3%)	1.19 (0.26, 4.50)	0.756	-
Swab (negative for SARS-CoV-2)	51:695 (6.8%)	12:301 (3.8%)	0.54 (0.26, 1.05)	0.064	-
Swab (positive for SARS-CoV-2)	317:429 (42.5%)	143:170 (45.7%)	1.14 (0.87, 1.50)	0.342	-
Discharge 30days					
CT thorax scan (negative for SARS-CoV-2)	4:742 (0.5%)	0:313 (0%)	0.00 (0.00, 3.61)	0.326	-
CT thorax scan (positive for SARS-CoV-2)	0:746 (0%)	1:312 (0.3%)	-	-	-
Swab (negative for SARS-CoV-2)	8:738 (1.1%)	0:313 (0%)	0.00 (0.00, 1.39)	0.114	-
Swab (positive for SARS-CoV-2)	75:671 (10.1%)	27:286 (8.6%)	0.85 (0.51, 1.36)	0.496	-
Diagnosis					
Positive SARS-CoV-2 swab - before surgery	122:624 (16.4%)	31:282 (9.9%)	0.56 (0.36, 0.86)	0.007	*
Positive SARS-CoV-2 swab - after surgery	409:337 (54.8%)	177:136 (56.5%)	1.07 (0.82, 1.41)	0.636	-

CT scan of the chest confirming SARS-CoV-2 - before surgery	20:726 (2.7%)	2:311 (0.6%)	0.23 (0.03, 0.97)	0.033	*
CT scan of the chest confirming SARS-CoV-2 - after surgery	9:737 (1.2%)	5:308 (1.6%)	1.33 (0.35, 4.46)	0.569	-
Clinical diagnosis or chest x-ray - suspected before time of surgery	39:707 (5.2%)	12:301 (3.8%)	0.72 (0.34, 1.43)	0.432	-
Clinical diagnosis or chest x-ray - suspected after time of surgery	67:679 (9%)	31:282 (9.9%)	1.11 (0.69, 1.77)	0.643	-

Table S4. SARS-CoV-2 symptoms data summaries by died (n = 313) and alive (n =746) groups. Data tabulated are counts, with estimated odds ratios (OR), with 95% confidence intervals, and p-values from Fisher's exact tests for each symptom.

Symptom	Alive (n = 746)	Died (n = 313)	OR (95% CI)	p-value	
	Y:N (%Y)	Y:N (%Y)			
Abdominal pain	11:735 (1.5%)	1:312 (0.3%)	0.21 (0.01, 1.49)	0.124	-
Breathlessness (dyspnoea)	54:692 (7.2%)	31:282 (9.9%)	1.41 (0.86, 2.28)	0.172	-
Cough	73:673 (9.8%)	35:278 (11.2%)	1.16 (0.73, 1.81)	0.505	-
Diarrhoea	8:738 (1.1%)	1:312 (0.3%)	0.30 (0.01, 2.22)	0.295	-
Fatigue	21:725 (2.8%)	10:303 (3.2%)	1.14 (0.47, 2.56)	0.695	-
Fever (>38 celsius)	61:685 (8.2%)	25:288 (8%)	0.98 (0.57, 1.61)	0.999	-
Haemoptysis	0:746 (0.0%)	0:313 (0.0%)	-	-	-
Myalgia	10:736 (1.3%)	3:310 (1%)	0.71 (0.13, 2.79)	0.765	-
Nausea/vomiting	13:733 (1.7%)	7:306 (2.2%)	1.29 (0.43, 3.52)	0.623	-
Sputum	8:738 (1.1%)	4:309 (1.3%)	1.19 (0.26, 4.50)	0.756	-
Other	311:435 (41.7%)	136:177 (43.5%)	1.08 (0.82, 1.42)	0.633	-

Table S5. Pre-surgery measures data (n, mean and sd) for the full study population (n = 1063), and died (n = 313) and alive (n =746) groups, and the difference in means between groups, with 95% confidence interval, and p-values from unpaired t-tests.

Measure	Full (n = 1063)		Alive (n = 746)		Died (n = 313)		Difference (95%CI)	p-value
	n	Mean (SD)	n	Mean (SD)	n	Mean (SD)		
Respiratory rate (breaths/minute)	996	17.75 (3.52)	706	17.65 (3.68)	289	18.02 (3.09)	-0.37 (-0.85, 0.11)	0.132
Heart rate (bpm)	1022	81.10 (14.63)	723	81.16 (14.59)	298	80.95 (14.77)	0.22 (-1.76, 2.20)	0.830
Systolic blood pressure (mmHg)	1023	138.15 (26.04)	724	138.33 (25.85)	298	137.61 (26.53)	0.72 (-2.80, 4.24)	0.687
Diastolic blood pressure (mmHg)	1021	72.97 (13.89)	723	73.07 (13.86)	297	72.70 (14.00)	0.37 (-1.52, 2.25)	0.703
Haemoglobin (g/L)	1062	117.94 (19.22)	745	118.35 (19.21)	313	116.93 (19.26)	1.41 (-1.13, 3.95)	0.276
White cell count (10^9/L)	1060	10.33 (4.26)	744	10.33 (4.36)	313	10.34 (4.04)	-0.01 (-0.57, 0.56)	0.976
C-reactive protein (mg/L)	738	54.70 (66.26)	514	54.84 (67.68)	221	54.63 (63.34)	0.21 (-10.28, 10.70)	0.969

Table S6. Operation details for the full study population (n = 1063), and died (n = 313) and alive (n =746) groups; p-values are for Fisher's exact tests comparing groups for each characteristic.

Characteristic	Full (n = 1063)	Alive (n = 746)	Died (n = 313)	Died (%)
Anaesthesia (p-value = 0.787)				
General	527 (49.6%)	368	157	29.9
Regional	524 (49.3%)	372	152	29.0
Missing	12 (27.8%)	6	4	33.3
*Pre-op respiration (p-value = 0.031 *)*				
None	714 (67.2%)	520	194	27.2
Oxygen	336 (31.6%)	220	115	34.3
Ventilated	2 (0.2%)	2	0	0.0
Missing	11(1.03%)	4	4	36.4
Pre-op delay (p-value = 0.220)				

< 6 hours	16 (1.5%)	11	5	31.2
6-23 hours	322 (30.3%)	232	88	27.5
24-47 hours	224 (21.1%)	147	77	34.4
48-71 hours	82 (7.7%)	61	21	25.6
72+ hours	123 (11.6%)	94	29	23.6
Missing	296 (27.8%)	201	93	31.4
*Procedure (p-value = 0.015 *)*				
LIMB - lower limb - total hip replacement	45 (4.2%)	41	4	8.9
LIMB - lower limb fracture - Cannulated Screws	5 (0.5%)	5	0	0.0
LIMB - lower limb fracture - Reduction and Internal Fixation	15 (1.4%)	10	5	33.3
LIMB - lower limb fracture - Dynamic Hip Screw	276 (26%)	195	81	29.3
LIMB - lower limb fracture - Reduction and Intramedullary Fixation	243 (22.9%)	169	73	30.2
LIMB - lower limb fracture - Partial Hip Replacement (Hemiarthroplasty)	479 (45.1%)	326	150	31.5
Missing	0 (0.0%)	0	0	0.0

Figure S1. Boxplots showing distributions of pre-surgery measures by outcome status (died or alive). Boxes show interquartile range (IQR), bars medians and whiskers are 1.5 times IQR.

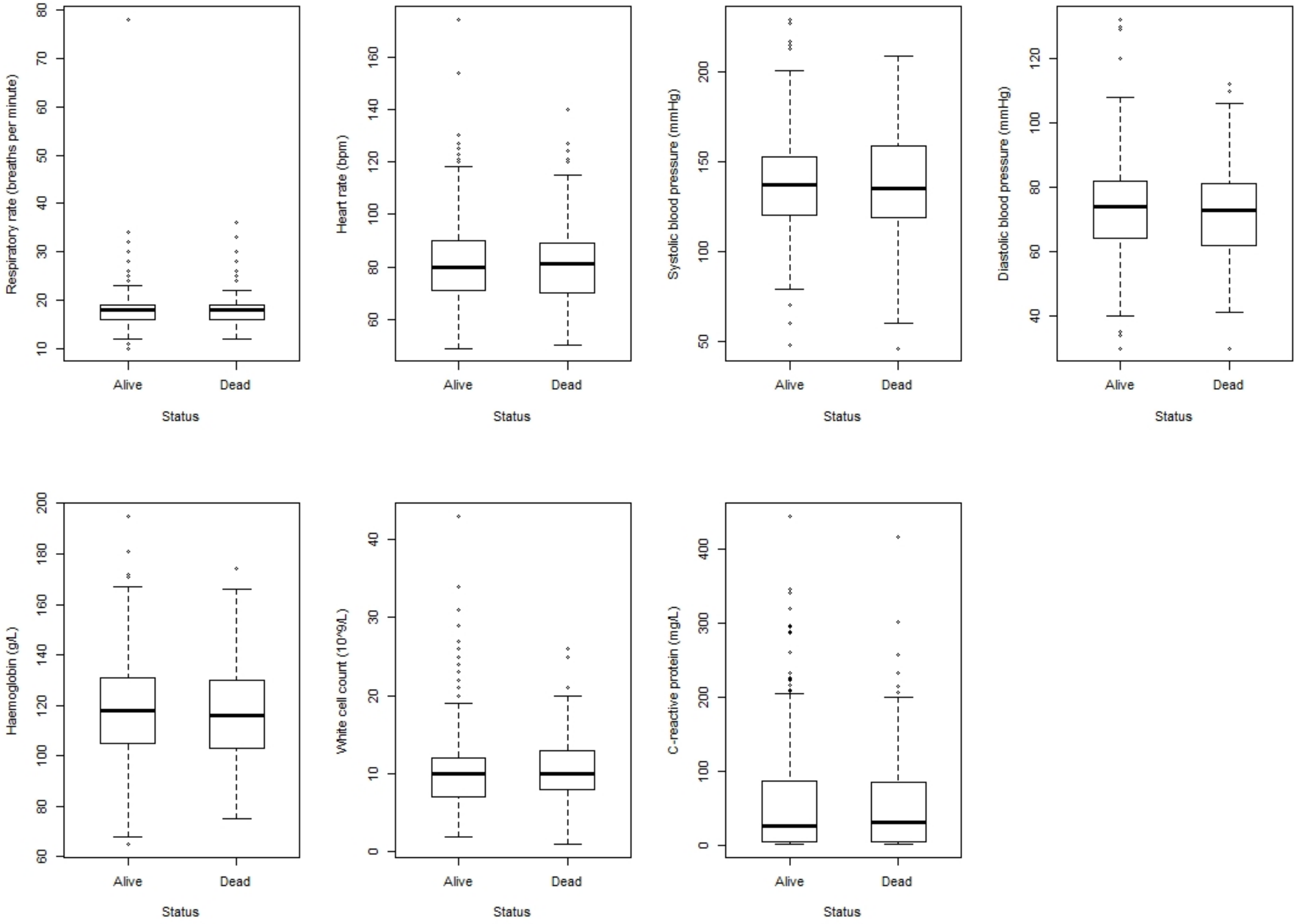
Respiratory rate (breaths per minute)
Heart rate (bpm)
Systolic blood pressure (mmHg)
Diastolic blood pressure (mmHg)
Haemoglobin (g/L)
White cell count (10^9/L)
C-reactive protein (mg/L)
Alive
Dead
Status

BMJ Publishing Group Limited (BMJ) disclaims all liability and responsibility arising from any reliance placed on this supplemental material which has been supplied by the author(s)
