# Supplementary material for: Outcomes after perioperative SARS-CoV-2 infection in patients with proximal femoral fractures: an international cohort study
Source: BMJ Open. 2021 Nov 30;11(11):e050830. doi: 10.1136/bmjopen-2021-050830 (PMC8634634; doi:10.1136/bmjopen-2021-050830)
Supplement: Author's manuscript [file bmjopen-2021-050830.draft_revisions.pdf]

BMJ Open is committed to open peer review. As part of this commitment we make the peer review history of every article we publish publicly available.

When an article is published we post the peer reviewers' comments and the authors' responses online. We also post the versions of the paper that were used during peer review. These are the versions that the peer review comments apply to.

The versions of the paper that follow are the versions that were submitted during the peer review process. They are not the versions of record or the final published versions. They should not be cited or distributed as the published version of this manuscript.

BMJ Open is an open access journal and the full, final, typeset and author-corrected version of record of the manuscript is available on our site with no access controls, subscription charges or pay-per-view fees (http://bmjopen.bmj.com).

If you have any questions on BMJ Open's open peer review process please email info.bmjopen@bmj.com

Outcomes after peri-operative SARS-CoV-2 infection in patients with proximal femoral fractures: an international cohort study

Journal:	*BMJ Open*
Manuscript ID	bmjopen-2021-050830
Article Type:	Original research
Date Submitted by the Author:	08-Mar-2021
Complete List of Authors:	Khatri, Chetan; University of Warwick,
Keywords:	COVID-19, Hip < ORTHOPAEDIC & TRAUMA SURGERY, TRAUMA MANAGEMENT

SCHOLARONE™
Manuscripts

I, the Submitting Author has the right to grant and does grant on behalf of all authors of the Work (as defined in the below author licence), an exclusive licence and/or a non-exclusive licence for contributions from authors who are: i) UK Crown employees; ii) where BMJ has agreed a CC-BY licence shall apply, and/or iii) in accordance with the terms applicable for US Federal Government officers or employees acting as part of their official duties; on a worldwide, perpetual, irrevocable, royalty-free basis to BMJ Publishing Group Ltd ("BMJ") its licensees and where the relevant Journal is co-owned by BMJ to the co-owners of the Journal, to publish the Work in this journal and any other BMJ products and to exploit all rights, as set out in our licence.

The Submitting Author accepts and understands that any supply made under these terms is made by BMJ to the Submitting Author unless you are acting as an employee on behalf of your employer or a postgraduate student of an affiliated institution which is paying any applicable article publishing charge ("APC") for Open Access articles. Where the Submitting Author wishes to make the Work available on an Open Access basis (and intends to pay the relevant APC), the terms of reuse of such Open Access shall be governed by a Creative Commons licence – details of these licences and which Creative Commons licence will apply to this Work are set out in our licence referred to above.

Other than as permitted in any relevant BMJ Author's Self Archiving Policies, I confirm this Work has not been accepted for publication elsewhere, is not being considered for publication elsewhere and does not duplicate material already published. I confirm all authors consent to publication of this Work and authorise the granting of this licence.

For peer review only

Outcomes after peri-operative SARS-CoV-2 infection in patients with proximal femoral fractures: an international cohort study

COVIDSurg Collaborative*

***Author contributions:** Full list of authors and roles within this research project are available in Supplementary files, titled Authorship (available to editors)

Correspondence: **Chetan Khatri**
Email: Chetan.khatri@gmail.com
Twitter: @ChetanKhatri2
Address: Warwick Clinical Trials Unit
Warwick Medical School, University of Warwick
Coventry, United Kingdom
CV4 7AL

Declarations

Competing interest: The collaborative group report no conflicts of interest. Funding is disclosed below.

Word count: 3361 (excluding tables and references)

Key words: Proximal fracture, SARS-CoV-2, COVID-19, Mortality

For peer review only

ABSTRACT

Objectives: Studies have demonstrated high rates of mortality in people with proximal femoral fracture and SARS-CoV-2, but there is limited published data on the factors that influence mortality for clinicians to make informed treatment decisions. This study aims to report the 30-day mortality associated with peri-operative infection of patients undergoing surgery for proximal femoral fractures and to examine the factors that influence mortality in a multi-variate analysis.

Setting: Prospective, international, multicentre, observational cohort study.

Participants: Patients undergoing any operation for a proximal femoral fracture from 1st February to 30th April 2020 and with perioperative SARS-CoV-2 infection (either 7-days prior, or 30-days post-operative).

Primary outcome: 30-day mortality. Multivariate modelling was performed to identify factors associated with 30-day mortality.

Results: This study reports included 1063 patients from 174 hospitals in 19 countries. Overall 30-day mortality was 29.4% (313/1063). In an adjusted model, 30-day mortality was associated with male gender (OR 2.29, 95% CI 1.68-3.13, p=0.000), age >80 years (OR 1.60, 95% CI 1.1-2.31, p=0.013), pre-operative diagnosis of dementia (OR 1.57, 95% CI 1.15-2.16, p=0.005), kidney disease (OR 1.73, 95% CI 1.18-2.55, p=0.005) and congestive heart failure (OR 1.62, 95% CI 1.06-2.48, p=0.025). 30-day mortality was lower in patients with a pre-operative diagnosis of SARS-CoV-2 (OR 0.6, 95% CI 0.6 (0.42-0.85), p=0.004). There was no difference in mortality in patients with an increase to delay in surgery (p=0.220), or type of anaesthetic given (p=0.787).

Conclusions: Patients undergoing surgery for a proximal femoral fracture with a peri-operative infection of SARS-CoV-2 have a high rate of mortality. This study would support the need for providing these patients with individualised medical and anaesthetic care, including medical optimisation before theatre. Careful pre-operative counselling is needed for those with a proximal femoral fracture and SARS-CoV-2, especially those in the highest risk groups.

For peer review only

ARTICLE SUMMARY

Strengths and limitations of this study

- This is a large, international, multicentre cohort study from which the results are generalisable across populations in other countries.
- This study described specific risk factors for mortality, which patients and those who care for them should use to make informed decisions regarding care.
- To our current knowledge, this is the largest cohort of patients undergoing surgery for a proximal femoral fractures with SARS-CoV-2 infection diagnosed peri-operatively
- There is not control arm to assess contemporaneous patients with undergoing an operation for proximal femoral fractures without SARS-CoV-2 infection during the height of the pandemic. However with high-quality data present pre-pandemic strongly suggests a substantial increase in mortality.

For peer review only

MAIN TEXT

Background

The rapid worldwide spread of Coronavirus Disease-2019 (COVID-19), caused by the Severe Acute Respiratory Syndrome CoronaVirus-2 (SARS-CoV-2) has had a severe effect on the elderly and frail population. A fracture of the proximal femur (neck of femur fracture) is a critical event in the elderly, frail population, with a high rate of death despite medical and surgical intervention [1]. Since 2007, there has been a steady improvement in mortality after a proximal femoral fracture with 6.1% of patients dying within 30 days of injury in the UK in 2018 [2]. However, the emergence of COVID-19 presents a new and unquantified risk to this particularly vulnerable group.

Proximal femur fractures represent a large international burden with incidence between 43 and 920 per 100,000 population [3]. As most fractures of the proximal femur happen as a result of trips or falls in the home, people have continued to present with this injury despite social restrictions [4, 5]. These patients typically have multiple co-morbidities and frailty is common [1]. Resultantly, they are particularly vulnerable to pulmonary complications [1, 6]. It is widely accepted that elderly patients with existing co-morbidities are at higher risks of critical illness and mortality due to COVID-19, potentially due to a higher preponderance to release pro-inflammatory cytokines that result in severe disease [7-9].

Clinicians have been swift to respond to this pandemic with large re-organisation of service provision [10, 11]. In response to this, the COVIDSurg collaborative

For peer review only

(www.globalsurg.org/covidsurg) has collected an international, large volume dataset to inform the global community of the safety of surgery in patients with peri-operative SARS-CoV-2 infection. The first report has demonstrated a 30-day mortality of 23.8% across patients undergoing any type of surgery [12]. Data published so far has reported a high mortality rate in a small cohort of patients with proximal femoral fractures positive for SARS-CoV-2 infection, with a maximum cohort size of 114 patients (range 10-114 patients) [13-20]. However few reports have the sample size sufficient to explore the factors that influence outcome. Further, large-scale data are required to explore pre-operative and operative variables that influence outcomes in order to inform the clinical decision-making processes.

Aims

The primary aim of this study is to determine the mortality rate observed in patients undergoing surgery for proximal femoral fracture with peri-operative SARS-CoV-2 infection. Secondarily, we aim to explore the patient and treatment factors associated with these outcomes.

For peer review only

Methods

Setting

This is an international, multicentre cohort study including consecutive patients who underwent surgery for proximal femoral fracture from 1st February 2020 to 30th April 2020. This study is a pre-planned sub-analysis of a larger, ongoing study designed to assess outcomes following all surgery for patients with perioperative SARS-CoV-2 infection [12].

The COVIDSurg collaborative is an international, multicentre, multidisciplinary team with individual collaborators collecting data locally, which is collated centrally. The collaborative methodology, which is well described and validated was used for this project [21]. The study protocol was registered online (ClinicalTrials.gov identifier: NCT04323644).

Ethics Review Board

This observational study collected anonymised routine clinical data, using an established international trainee collaborative model [22]. Within the United Kingdom this was registered as a clinical audit or service evaluation at each participating trust following individual hospital policies and procedures, prior to initiating data collection at that site. In other countries, the principal investigator was responsible for obtaining local approval in line with local and/or national guidelines. In some participating countries, informed patient consent was taken, whilst in others the requirement was waived by local research committees. Country-specific

For peer review only

guidelines for site set-up were published on a dedicated study website (www.globalsurg.org/covidsurg).

Inclusion Criteria

Participating hospitals included consecutive patients undergoing surgery for proximal femoral fractures that had SARS-CoV-2 infection diagnosed either 7 days pre-operatively, or up to 30-days post-operatively. For those patients who underwent multiple procedures, the procedure closest to the time of confirmation of SARS-CoV-2 infection was defined as the index procedure.

Patients received laboratory confirmation of SARS-CoV-2 using quantitative Reverse Transcription Polymerase Chain Reaction (qRT-PCR). As qRT-PCR is not available in all participating hospitals, patients were included if their diagnosis was made by clinical or radiological findings. Clinical diagnosis was made in patients presenting with symptoms and a clinical pattern of COVID-19. These included cough, fever and/or myalgia [23]. Radiological diagnosis was made through computed tomography (CT) scanning of the thorax according to local protocols. All patients who were included solely on clinical or radiological suspicion but had a subsequent negative test were excluded from the database by individual collaborators.

Diagnosis

This study includes all patients identified as having an operation for a proximal femoral fracture. The diagnosis was established pragmatically by the local site teams according to their assessment of the fracture. The reported data was screened by a

For peer review only

central dedicated data cleaning team, with only confirmed proximal femoral fractures included in the cohort.

Patient Identification

Researchers at participating centres screened consecutive patients undergoing surgery to ensure all patients were identified. The study was initiated in some countries after their peak of infection, and therefore retrospective identification and data collection was permitted, as long as the data collection was consecutive at that site.

To reduce selection bias, a variety of written materials were distributed to site leads to highlight possible methods of identifying patients ensuring all eligible patients were included. Investigators were invited to social media groups and online teleconferences to trouble-shoot recruitment issues, share learning and ensure consistent recruitment into the wider cohort.

Outcome measures

The primary outcome measure was 30-day all-cause mortality, with the day of surgery defined as day zero. The secondary outcome measure was rate of pulmonary complications, which is a composite outcome defined previously from the Prevention of Respiratory Insufficiency after Surgical Management (PRISM) randomised controlled trial [24, 25].

Pulmonary complications were defined as pneumonia, acute respiratory distress syndrome (ARDS), and/or unexpected post-operative ventilation; these have been identified as the most frequent COVID-19-related pulmonary complications in

For peer review only

medical patients [23]. Unexpected post-operative ventilation was defined as either (i) any episode of non-invasive ventilation, invasive ventilation, or extracorporeal membrane oxygenation after initial extubation following surgery, or (ii) unexpected failure to extubate following surgery [12].

Data collection and quality assurance

Data was collected online using the Research Electronic Data Capture (REDCap) web application [26]. Demographic variables recorded consisted of age, sex, and American Society of Anesthesiologists physical status classification (ASA). Age was collected as a categorical variable by deciles of age. ASA at the time of surgery was dichotomized to (i) grades 1-2 and (ii) grades 3-5 for the purpose of analysis, time to surgery to (i) under 24 hours, (ii) 24-48 hours, and (iii) over 48 hours, and surgery to (i) hemiarthroplasty, (ii) total hip replacement, (iii) dynamic hip screw, (iv) cannulated screws and (v) intramedullary nail. The timing of SARS-CoV-2 diagnosis was recorded as either preoperative or post-operative.

Before data was entered into analysis, site principle investigators were required to confirm all consecutive eligible cases had been completed and uploaded. Where diagnosis was unclear, authors were contacted for clarification.

Statistical analysis

The study was reported according to STROBE (Strengthening the Reporting of Observational Studies in Epidemiology) guidelines [27]. Proportions are expressed with 95% confidence intervals and the mean and 95% confidence intervals were used where data were assumed to be approximately normally distributed. Fishers exact test

For peer review only

was used for categorical data. Non-parametric data was summarised with the median and interquartile ranges. Statistical significance was assessed at the 5% level.

The risk of death at 30 days was chosen as the primary outcome for the study. Mixed-effects logistic regression analysis was used to assess the strength and significance of associations between a number of explanatory variables and death within 30-days. Random effects were included in the mixed-effects model to account for the hierarchical structure of the data (individual hospital effects are naturally nested within country effects) and fixed effects were included to adjust for a range of pre-operative variables that may influence mortality in this population, and relevant factors related to the injury or treatment (e.g. type of operation, time from admission to operation, type of anaesthetic). An additional analysis of the same factors was undertaken using the same model structure for the secondary outcome of pulmonary complications. All analyses were implemented in R (R Core Team (2020). R: A language and environment for statistical computing. R Foundation for Statistical Computing, Vienna, Austria. URL https://www.R-project.org/.).

Patient and Public Involvement

Patients were not involved in the design, conduct or reporting of this study.

For peer review only

Results

Population

This study returned 30-day follow up for 1063 patients with proximal femoral fractures. Data was collected in 174 hospitals from 19 countries (Supplementary Table S1). Of these, 65.5% were female (696/1063). 7.8% (83/1063) patients were <70 years old, 17.8% (189/1063) were between 70-79 years, 47.7% (507/1063) were between 80-89 and 26.7% (284/1063) were 90+ years old.

Mortality

Overall 30-day mortality was 29.4% (313/1063). With each decile of age, mortality significantly increased, being highest in those patients >90 years old (38.7% [110/284], p=0.001).

In an adjusted model (Figure 1), 30-day mortality was associated with male gender (OR 2.29, 95% CI 1.68-3.13, p=0.000), age >80 years (OR 1.60, 95% CI 1.1-2.31, p=0.013), diagnosis of dementia (OR 1.57, 95% CI 1.15-2.16, p=0.005), chronic kidney disease (OR 1.73, 95% CI 1.18-2.55, p=0.005) and congestive heart failure (OR 1.62, 95% CI 1.06-2.48, p=0.025). 30-day mortality was lower in patients with a pre-operative diagnosis of SARS-CoV-2 (OR 0.60, 95% CI 0.42-0.85, p=0.004). Non-adjusted values are presented in Table S2.

Pulmonary complications

In an adjusted model (Figure 2), respiratory complications were associated with male gender (OR 1.7, 95% CI 1.27-2.28, p=0.000), diagnosis of dementia (OR 1.34, 95%

For peer review only

CI 1.01-1.79, p=0.044) and congestive heart failure (OR 1.76, 95% CI 1.17-2.63, p=0.006). Chronic Obstructive Pulmonary Disorder (COPD) showed a signal to be a risk factor, however was not significantly associated, (OR 1.42, 95% CI 0.96-2.09, p=0.076)

Diagnosis

The majority of diagnosis of SARS-CoV-2 was made via PCR swab testing 93.3% (992/1063) (Table S1 & S3) and there was no difference in mortality between those diagnosed clinically (p=0.668). The majority of patients received a diagnosis post-operatively 69% (733/1063).

Pre-operative variables

Pre-operative symptoms (Table S4), including breathlessness, cough and fever (>38 ° Celsius) were not significantly different in patients who were alive or dead at 30 days post-operatively. On examination of pre-operative observations, a high respiratory rate was predictive of mortality (OR 1.73 95% CI 1.18-2.55, p=0.025) (Figure 1). However, there was no significant difference in patient's heart rate, systolic or diastolic blood pressure (Table S5, Figure S1) between those who were alive or dead at 30 days.

Those patients with ASA grade 3-5 had a significantly higher mortality of 31.4% (281/899) versus ASA of 1-2 of 18.5% (28/151), p=0.001.

Procedures

For peer review only

The operations were carried out under a general anaesthetic in 49.6% (527/1063) of patients (Table S6). 67.2% (714/1063) of patients did not require any pre-operative oxygen therapy. 31.8% (338/1063) of patients had their operation within 24 hours of presentation to hospital, 21.1% (224/1063) had their operation between 24-47 hours and 19.2% (205/1063) of patients had their operation after 48 hours of presentation to hospital.

45.1% (479/1063) of patients underwent hemiarthroplasty with a further 4.2% undergoing total hip replacement (45/1063). For patients who underwent fixation, 26% (276/1063) underwent Dynamic Hip Screw (DHS) fixation, 22.9% (243/1063) patients underwent intramedullary fixation, 0.5% (5/1063) underwent cannulated screw fixation whilst a further 1.4% (15/1053) underwent internal fixation.

There was no difference in mortality between patients undergoing general and regional anaesthesia (29.9% [157/527 versus 29.0% [152/524], p=0.787). However, there was an increased mortality in those patients requiring pre-operative oxygen therapy (34.3% [115/336] versus 27.2% [194/714], p=0.031)

There was no significant difference in mortality for patients with delayed operation. The highest mortality was for patients operated between 24-47 hours of admission (34.4%, [77/224]) but was not significantly higher than less than those operated after 48 hours (p=0.220).

Mortality was highest in March (33.7%, 159/474) compared to April (27.0%, 150/558), and February (11.5%, 3/26), p=0.007 (Table S1).

For peer review only

Data Sharing

No additional data available. Requests for raw data can be requested via the corresponding author.

For peer review only

Discussion

The 30-day mortality rate for patients with a peri-operative diagnosis of SARS-CoV-2 infection undergoing surgery for proximal femoral fracture is substantial. An overall rate of 29.4% compares to the reported 30-day mortality for fracture neck of femur in the UK National Hip Fracture Database of 6.1% in 2018 [2]. Further, elderly patients, and those with medical comorbidities such as dementia, chronic kidney disease and congestive heart failure were associated with higher risk of 30-day mortality. Notably, patients with a pre-operative diagnosis of SARS-CoV-2 infection had lower rates of 30-day mortality, likely reflecting early recognition and closer management of these patients. Findings from this study will be useful in guiding clinicians to identify high-risk patients that may warrant closer medical and surgical input during the COVID-19 pandemic.

Considering this high mortality, it is critical that patients with proximal femoral fractures are protected from contracting SARS-CoV-2 in the peri-operative period. A study by Kayani et al. has suggested that half of infections in patients with proximal femoral fractures occur in hospital, as denoted by having negative pre-operative samples [17]. Similarly, a study by Hall et al. has suggested nearly half of cases were due to nosocomial transmission [28]. Within this study, 733 (69%) of infections were diagnosed post-operatively. This may infer that infections have been transferred in hospital, although due to incubation period of the virus, it is hard to know the proportion that contracted the virus prior to presentation or in hospital. [7, 29] Higher mortality was observed in people who had a post-operative diagnosis, which emphasises the critical importance of avoiding in-hospital transmission. Hospitals

For peer review only

should consider implementation of careful infection control processes to minimise and prevent transmission of SARS-CoV-2 infection. Within the elective setting, the creation of COVID-19-free surgical pathways for elective patients has been shown to reduce infection and subsequent mortality [30-32] and whilst only some of the principles are transferrable to the emergency setting, it demonstrates the value of meticulous infection control processes throughout the hospital stay.

With the added risk associated with SARS-CoV-2 infection, and the need to continue managing this injury despite the ongoing pandemic, it is important for data to be used as part of the informed consent process. In patients with multiple high-risk factors such as those who are more elderly, have respiratory and cardiac co-morbidities, non-operative management may be considered following an appropriate discussion with the patient and/or their family. Every year in the UK, 2.5% of hip fractures are treated non-operatively [33]. A study performed before the pandemic reported that the mortality within thirty days for conservatively treated patients was 31.3% [34]. We do not know the mortality from non-operative management during the pandemic for patients with SARS-Cov-2, but the particularly high mortality associated with surgery in high-risk groups may change the balance of benefit and harm towards conservative treatment and this should be considered.

The 30-day mortality of 29.4% identified within this study is comparable to published literature, in the UK (range from 16.3%-35.6%) [15-17, 19, 28, 35], Italy (18.75%) [14], Spain (30.4%) [13] and the USA (range from 35.3%-56%) [18, 20]. From a study within the UK, the authors also found a correlation between male sex and increased mortality (OR 2.69), which is similar to that demonstrated in this study (OR

For peer review only

2.29) [16]. Additionally, another UK study reported having more than three co-morbidities as a risk factor for mortality [17]. This study has specifically delineated a diagnosis of dementia, chronic kidney disease and congestive heart failure as being independent risk factors for mortality. In a study from USA, the authors found those patients who died were older with multiple co-morbidities and this was reflected in statistically significant higher ASA scores in comparison to their negative counterparts [20].

This study found that there was no significant increase in mortality with delay to surgery. Current guidelines suggest early surgery should be undertaken [36] and this is associated with lower mortality [37]. This would suggest that those patients at the highest risk of mortality can have medical optimisation if appropriate and will not result in a higher mortality from SARS-CoV-2 infection. Similarly, previous studies have found a higher rate of mortality in patients undergoing general versus regional anaesthesia for proximal femoral fractures [38, 39]. This study reports no difference between general and regional anaesthetic (29.9% versus 29.0%, p=0.787). As a result, anaesthetic decisions in this population should not be influenced by a positive test for SARS-CoV-2. Out of all clinical features, respiratory rate at presentation was associated with higher mortality. Clinicians should focus on this as an important finding when counselling patients of their peri-operative mortality.

To our knowledge, this is the largest cohort of patients undergoing surgery for a proximal femoral fractures with SARS-Cov-2 infection diagnosed peri-operatively. This study was conducted in multiple centres, internationally, allowing it to be generalisable across populations in other countries.

For peer review only

Limitations

This study was conducted in hospitals in the early to mid-phase of the pandemic where routine testing was not available in all participating centres. As such, to be pragmatic, patients were included if a clinical diagnosis was made by the treating physician. Protocols were not standardised for clinical diagnosis and were left the senior treating physician. Laboratory diagnosis was made by qRT-PCR, from which false-negative results may have excluded patients from analysis. Indeed, the sensitivity of qRT-PCR testing for has shown to be as low as 32% for throat swabs [40]. However, in patients with negative results and high clinical suspicion of SARS-CoV-2 infection, multiple samples are often taken, including broncho-alveolar lavage. Thus, the number of patients excluded is expected to be low. Whilst this study reports a higher mortality from post-operative diagnosis of SARS-CoV-2 infection, it is unclear whether the infection was contracted pre-operatively or not, as has been discussed above.

This study does not have a control arm, assessing contemporaneous patients with undergoing an operation for proximal femoral fractures without SARS-CoV-2 infection during the height of the pandemic. However, comparison with high-quality pre-pandemic data strongly suggests a substantial increase in mortality. Patients and those who care for them should consider this carefully when making decisions in this common and challenging clinical scenario.

Conclusion

For peer review only

Patients undergoing surgery for a proximal femoral fracture with a peri-operative infection of SARS-CoV-2 have a high rate of mortality. The study would support the approach of providing these patients with individualised medical and anaesthetic care, including medical optimisation before theatre. It is imperative to prevent transmission of coronavirus in the hospital setting. Careful pre-operative counselling is needed for those with a proximal femoral fracture and SARS-CoV-2, especially those in the highest risk groups.

For peer review only

Tables & Figures

Fig. 1 Mixed-effects logistic regression model for 30 day mortality

Fig. 2. Mixed-effects logistic regression model for pulmonary complications

Funding

This work was supported by a National Institute for Health Research (NIHR) Global Health Research Unit Grant (NIHR 16.136.79), the Association of Coloproctology of Great Britain and Ireland, Association of Upper Gastrointestinal Surgeons, Bowel Disease Research Foundation, Yorkshire Cancer Research, Sarcoma UK, the British Association of Surgical Oncology, the Vascular Society for Great Britain and Ireland, and the European Society of Coloproctology. The funders had no role in study design, data collection, analysis and interpretation, or writing of this report. The views expressed are those of the authors and not necessarily those of the National Health Service, the NIHR, or the UK Department of Health and Social Care

For peer review only

References

1. Kistler, E.A., et al., *Frailty and short-term outcomes in patients with hip fracture.* Geriatric orthopaedic surgery & rehabilitation, 2015. **6**(3): p. 209-214.
2. Physicians, R.C.o., *National Hip Fracture Database (NHFD) Annual Report 2019.* 2019.
3. Dhanwal, D.K., et al., *Epidemiology of hip fracture: worldwide geographic variation.* Indian journal of orthopaedics, 2011. **45**: p. 15-22.
4. Hampton, M., et al., *The effects of a UK lockdown on orthopaedic trauma admissions and surgical cases: A multicentre comparative study.* Bone & Joint Open, 2020. **1**(5): p. 137-143.
5. Scott, C.E., et al., *Population mobility and adult orthopaedic trauma services during the COVID-19 pandemic: fragility fracture provision remains a priority.* Bone & Joint Open, 2020. **1**(6): p. 182-189.
6. Lawrence, V.A., et al., *Medical complications and outcomes after hip fracture repair.* Archives of internal medicine, 2002. **162**(18): p. 2053-2057.
7. Huang, C., et al., *Clinical features of patients infected with 2019 novel coronavirus in Wuhan, China.* Lancet, 2020. **395**(10223): p. 497-506.
8. Besnier, E., J.J. Tuech, and L. Schwarz, *We Asked the Experts: Covid-19 Outbreak: Is There Still a Place for Scheduled Surgery? "Reflection from Pathophysiological Data".* World J Surg, 2020. **44**(6): p. 1695-1698.
9. Meftahi, G.H., et al., *The possible pathophysiology mechanism of cytokine storm in elderly adults with COVID-19 infection: the contribution of "inflame-aging".* Inflammation Research, 2020: p. 1-15.
10. Chang Liang, Z., et al., *Novel Coronavirus and Orthopaedic Surgery: Early Experiences from Singapore.* J Bone Joint Surg Am, 2020.
11. Thornton, J., *Clinicians are leading service reconfiguration to cope with covid-19.* Bmj, 2020. **369**: p. m1444.
12. Collaborative, C., *Mortality and pulmonary complications in patients undergoing surgery with perioperative SARS-CoV-2 infection: an international cohort study.* The Lancet, 2020.
13. Munoz Vives, J.M., et al., *Mortality Rates of Patients with Proximal Femoral Fracture in a Worldwide Pandemic: Preliminary Results of the Spanish HIP-COVID Observational Study.* J Bone Joint Surg Am, 2020.
14. Catellani, F., et al., *Treatment of Proximal Femoral Fragility Fractures in Patients with COVID-19 During the SARS-CoV-2 Outbreak in Northern Italy.* J Bone Joint Surg Am, 2020.
15. Mamarelis, G., et al., *Mortality in Patients with Proximal Femoral Fracture During the COVID-19 Pandemic: A UK Hospital's Experience.* JBJS Open Access, 2020. **5**(4).
16. Rasidovic, D., et al., *Impact of COVID-19 on clinical outcomes for patients with fractured hip: a multicentre observational cohort study.* Bone & Joint Open, 2020. **1**(11): p. 697-705.
17. Kayani, B., et al., *The effects of COVID-19 on perioperative morbidity and mortality in patients with hip fractures.* Bone Joint J, 2020. **102-b**(9): p. 1136-1145.
18. Egol, K.A., et al., *Increased Mortality and Major Complications in Hip Fracture Care During the COVID-19 Pandemic: A New York City Perspective.* J Orthop Trauma, 2020. **34**(8): p. 395-402.
19. Thakrar, A., et al., *Thirty-Day Mortality Rate of Patients With Hip Fractures During the COVID-19 Pandemic: A Single Centre Prospective Study in the United Kingdom.* J Orthop Trauma, 2020. **34**(9): p. e325-e329.
20. LeBrun, D.G., et al., *Hip fracture outcomes during the COVID-19 pandemic: early results from New York.* Journal of orthopaedic trauma, 2020.

For peer review only

21. Bhangu, A., et al., *Surgical research collaboratives in the UK.* Lancet, 2013. **382**(9898): p. 1091-2.
22. Collaborative, C., *COVIDSurg Protocol*. 2020.
23. Zhou, F., et al., *Clinical course and risk factors for mortality of adult inpatients with COVID-19 in Wuhan, China: a retrospective cohort study.* Lancet, 2020. **395**(10229): p. 1054-1062.
24. Abbott, T.E.F., et al., *A systematic review and consensus definitions for standardised end-points in perioperative medicine: pulmonary complications.* Br J Anaesth, 2018. **120**(5): p. 1066-1079.
25. Pearse, R.M., et al., *The Prevention of Respiratory Insufficiency after Surgical Management (PRISM) Trial. Report of the protocol for a pragmatic randomized controlled trial of CPAP to prevent respiratory complications and improve survival following major abdominal surgery.* Minerva Anestesiol, 2017. **83**(2): p. 175-182.
26. Harris, P.A., et al., *Research electronic data capture (REDCap)—a metadata-driven methodology and workflow process for providing translational research informatics support.* Journal of biomedical informatics, 2009. **42**(2): p. 377-381.
27. Gharaibeh, A., S. Koppikar, and F.J. Bonilla-Escobar, *Strengthening the Reporting of Observational Studies in Epidemiology (STROBE) in the International Journal of Medical Students.* International Journal of Medical Students, 2014. **2**(2): p. 36-37.
28. Hall, A.J., et al., *IMPACT-Scot 2 report on COVID-19 in hip fracture patients: a nationwide study of mortality, risk factors for community and hospital acquired COVID-19, and suggested care pathways.* The Bone & Joint Journal, 2021. **103**: p. 1-10.
29. Phua, J., et al., *Intensive care management of coronavirus disease 2019 (COVID-19): challenges and recommendations.* Lancet Respir Med, 2020. **8**(5): p. 506-517.
30. Collaborative, C., *Preoperative nasopharyngeal swab testing and postoperative pulmonary complications in patients undergoing elective surgery during the SARS-CoV-2 pandemic.* The British Journal of Surgery, 2020.
31. Chui, K., A. Thakrar, and S. Shankar, *Evaluating the efficacy of a two-site ('COVID-19'and 'COVID-19-free') trauma and orthopaedic service for the management of hip fractures during the COVID-19 pandemic in the UK.* Bone & Joint Open, 2020. **1**(6): p. 190-197.
32. Glasbey, J.C., A. Bhangu, and C. Collaborative, *Elective cancer surgery in COVID-19–free surgical pathways during the SARS-CoV-2 pandemic: an international, multicenter, comparative cohort study.* Journal of Clinical Oncology, 2020: p. JCO. 20.01933.
33. Databse, N.H.F. *National Hip Fracture Report*. 2020; Available from: https://www.nhfd.co.uk/20/NHFDcharts.nsf/vwcharts/Surgery?opendocument .
34. Moulton, L., et al., *Outcome after conservatively managed intracapsular fractures of the femoral neck.* The Annals of The Royal College of Surgeons of England, 2015. **97**(4): p. 279-282.
35. Mahmood, A., et al., *Coronavirus infection in hip fractures (CHIP) study.* The Bone & Joint Journal, 2021: p. 1-5.
36. Hoffenberg, R., et al., *Fractured neck of femur: Prevention and management. Summary and recommendations of a report of the Royal College of Physicians.* Journal of the Royal College of Physicians of London, 1989. **23**(1): p. 8-14.
37. Rogers, F.B., S.R. Shackford, and M.S. Keller, *Early fixation reduces morbidity and mortality in elderly patients with hip fractures from low-impact falls.* Journal of Trauma and Acute Care Surgery, 1995. **39**(2): p. 261-265.
38. Ahn, E.J., et al., *Comparison of general anaesthesia and regional anaesthesia in terms of mortality and complications in elderly patients with hip fracture: a nationwide population-based study.* BMJ open, 2019. **9**(9): p. e029245.

For peer review only

39. Van Waesberghe, J., et al., *General vs. neuraxial anaesthesia in hip fracture patients: a systematic review and meta-analysis.* BMC anesthesiology, 2017. **17**(1): p. 87.

40. Wang, W., et al., *Detection of SARS-CoV-2 in Different Types of Clinical Specimens.* Jama, 2020. **323**(18): p. 1843-1844.

For peer review only

Authorship (all co-authors PubMed citable)

To be published under collaborative authorship under group:

COVIDSurg Collaborative

Authorship contribution is listed as below, according to role within collaborative:

Writing group: Chetan Khatri, Alex E Ward, Dmitri Nepogodiev, Imran Ahmed, Daoud Chaudhry, Fatima Dhaif, Brittany Bankhad-Kendall, Haytham Kaafarani, Chris Bretherton, Ansar Mahmood, Leonard Marais, Nicholas Parsons, Aneel Bhangu, Andy Metcalfe (senior author & overall guarantor).

Statistical analysis: Nicholas Parsons (lead statistician), Chetan Khatri.

Operations Committee: Kwabena Siaw-Acheampong, Daoud Chaudhry, Brett E Dawson, Jonathan P Evans, James C Glasbey, Rohan R Gujjuri, Emily Heritage, Conor S Jones, Sivesh K Kamarajah, Chetan Khatri, James M Keatley, Samuel Lawday, Elizabeth Li, Siobhan C Mckay, Dmitri Nepogodiev, Gianluca Pellino, Abhinav Tiwari, Joana FF Simoes, Isobel M Trout, Mary L Venn, Richard JW Wilkin, Aneel Bhangu.

Dissemination Committee: Adesoji O Ademuyiwa, Arnav Agarwal, Ehab Al Ameer, Derek Alderson, Osaid Alser, Alexis P Arnaud, Knut Magne Augestad, Brittany Bankhead-Kendall, Ruth A Benson, Sohini Chakrabortee, Ruth Blanco-Colino, Amanpreet Brar, Ana Minaya Bravo, Kerry A Breen, Igor de Lima Buarque, Edward Caruana, Miguel F Cunha, Giana H Davidson,

For peer review only

Anant Desai, Salomone Di Saverio, John Edwards, Muhammed Elhadi, Shebani Farik, Marco Fiore, J Edward Fitzgerald, Samuel Ford, Gaetano Gallo, Dhruv Ghosh, Gustavo Mendonça Ataíde Gomes, Ewen Griffiths, Constantine Halkias, Ewen M Harrison, Peter Hutchinson, Arda Isik, Haytham Kaafarani, Angelos Kolias, Ismail Lawani, Hans Lederhuber, Sezai Leventoglu, Markus W Loffler, Janet Martin, Hassan Mashbari, Dennis Mazingi, Helen Mohan, Rachel Moore, David Moszkowicz, Joshua S Ng-Kamstra, Symeon Metallidis, Susan Moug, Milagros Niquen, Faustin Ntirenganya, Oumaima Outani, Francesco Pata, Thomas D Pinkney, Peter Pockney, Dejan Radenkovic, Antonio Ramos-De la Medina, Keith Roberts, Irene Santos, Andrew Schache, Andreas Schnitzbauer, Grant D Stewart, Richard Shaw, Sebastian Shu, Kjetil Soreide, Antonino Spinelli, Sudha Sundar, Stephen Tabiri, Philip Townend, Georgios Tsoulfas, Gabrielle van Ramshorst, Raghavan Vidya, Dale Vimalachandran, Naomi Wright, EuroSurg, European Society of Coloproctology, GlobalSurg, GlobalPaedSurg, ITSurg, PTSurg, SpainSurg, S-ECCO, Joana FF Simoes (chair).

Local Principal Investigators: To be confirmed.

Collaborators: To be confirmed

Data Monitoring and Ethics Committee: Deborah S Keller, Neil J Smart

For peer review only

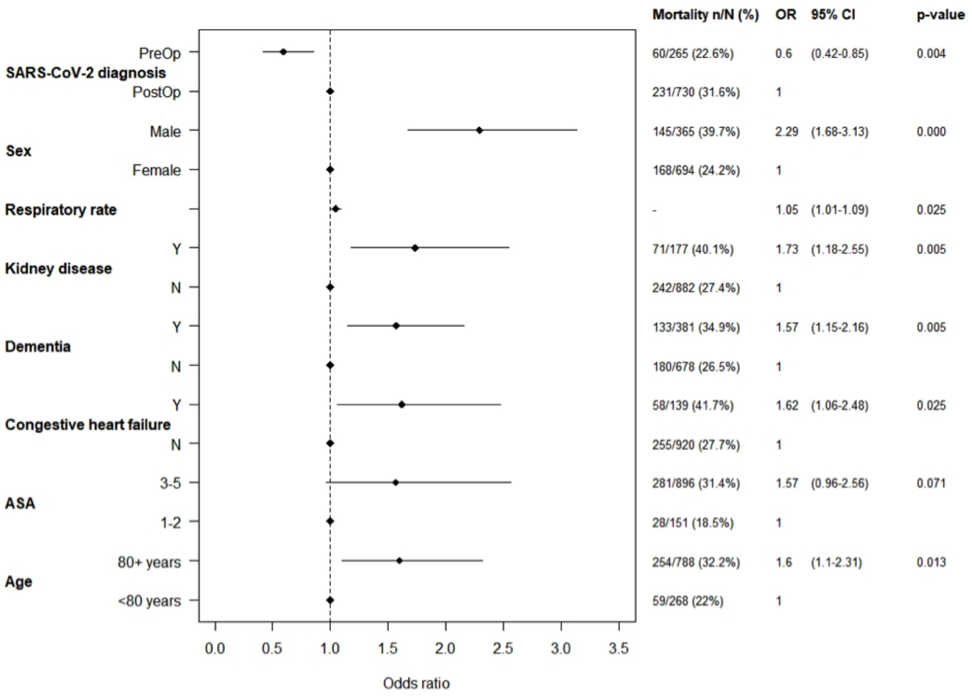

Fig. 1 Mixed-effects logistic regression model for 30 day mortality

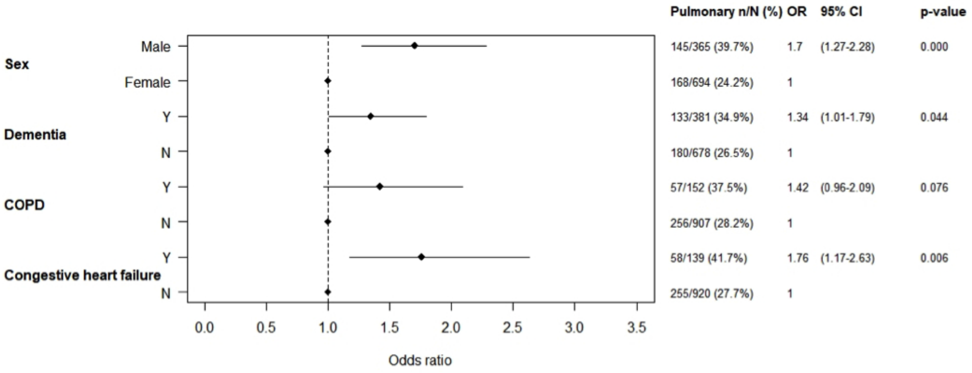

Fig. 2. Mixed-effects logistic regression model for pulmonary complications

Supplementary Material

Table S1. Baseline characteristics (sex, age, ASA grade, cardiac risk, time of diagnosis, method of diagnosis and month study participant recruited) of the full study population (n = 1063), and dead (n = 313) and alive (n =746) groups; p-values are for Fisher's exact tests comparing groups for each characteristic.

Characteristic	Full (n = 1063)	Alive (n = 746)	Dead (n = 313)	Dead (%)
*Sex (p-value = <0.001 ***)*				
Female	696 (65.5%)	526	168	24.2
Male	367 (34.5%)	220	145	39.7
Missing	0 (0.0%)	0	0	0.0
*Age (p-value = 0.001 **)*				
20-29 years	3 (0.3%)	3	0	0.0
30-39 years	2 (0.2%)	1	1	50.0
40-49 years	4 (0.4%)	3	1	25.0
50-59 years	24 (2.3%)	21	3	12.5
60-69 years	50 (4.7%)	39	11	22.0
70-79 years	189 (17.8%)	145	43	22.9
80-89 years	507 (47.7%)	360	144	28.6
90+ years	284 (26.7%)	174	110	38.7
Missing	0(0.0%)	0	0	0.0
*ASA (p-value = 0.001 **)*				
1-2	151 (14.2%)	123	28	18.5
3-5	899 (84.6%)	615	281	31.4
Missing	13(1.2%)	8	4	30.8

For peer review only

*Cardiac risk (p-value = <0.001 ***)*				
0	487 (45.8%)	372	114	23.5
1	349 (32.8%)	238	110	31.6
2	169 (15.9%)	106	61	36.5
3	44 (4.1%)	23	21	47.7
4	8 (0.8%)	5	3	37.5
5	1 (0.1%)	0	1	100.0
Missing	5 (0.47%)	2	3	60.0
*Time of diagnosis (p-value = 0.006 **)*				
Post-op	733 (69%)	499	231	31.6
Pre-op	266 (25%)	205	60	22.6
Missing	64 (6.0%)	42	22	34.4
Diagnosis (p-value = 0.668)				
Clinical	62 (5.8%)	42	20	32.3
Swab	992 (93.3%)	696	292	29.6
Missing				
*Month (p-value = 0.007 **)*				
February	26 (2.4%)	23	3	11.5
March	474 (44.6%)	313	159	33.7
April	558 (52.5%)	406	150	27.0
Missing	1(0.09%)	0	1	100.0

For peer review only

Table S2. Comorbidity data summaries by dead (n = 313) and alive (n =746) groups. Data tabulated are counts, with estimated odds ratios (OR), with 95% confidence intervals, and p-values from Fisher's exact tests for each comorbidity.

Comorbidity	Alive	Dead	OR	p-value

	(n = 746)	(n = 313)	(95% CI)		
	Y:N (%Y)	Y:N (%Y)			
Current smoker	34:712 (4.6%)	9:304 (2.9%)	0.62 (0.26, 1.34)	0.235	-
Asthma	53:693 (7.1%)	21:292 (6.7%)	0.94 (0.53, 1.62)	0.895	-
Current cancer diagnosis	57:689 (7.6%)	24:289 (7.7%)	1.00 (0.58, 1.68)	0.999	-
Chronic kidney disease (moderate/severe)	106:640 (14.2%)	71:242 (22.7%)	1.77 (1.25, 2.51)	0.001	**
Chronic obstructive pulmonary disease (COPD)	95:651 (12.7%)	57:256 (18.2%)	1.53 (1.05, 2.21)	0.027	*
Congenital abnormality - cardiac	4:742 (0.5%)	1:312 (0.3%)	0.60 (0.01, 6.04)	0.999	-
Congenital abnormality - non-cardiac	0:746 (0.0%)	4:309 (1.3%)	-	-	-
Congestive heart failure	81:665 (10.9%)	58:255 (18.5%)	1.87 (1.27, 2.73)	<0.001	***
Dementia	248:498 (33.2%)	133:180 (42.5%)	1.48 (1.12, 1.96)	0.005	**
Diabetes mellitus	142:604 (19.0%)	63:250 (20.1%)	1.07 (0.76, 1.51)	0.671	-
Hypertension	387:359 (51.9%)	186:127 (59.4%)	1.36 (1.03, 1.79)	0.026	*
Myocardial infarction or ischemic heart disease	103:643 (13.8%)	63:250 (20.1%)	1.57 (1.09, 2.25)	0.012	*
Peripheral vascular disease	34:712 (4.6%)	21:292 (6.7%)	1.51 (0.82, 2.72)	0.172	-
Stroke/ TIA	107:639 (14.3%)	57:256 (18.2%)	1.33 (0.92, 1.92)	0.114	-
Other (including other lung disease)	377:369 (50.5%)	158:155 (50.5%)	1.00 (0.76, 1.31)	0.999	-

Table S3. Diagnosis data summaries by dead (n = 313) and alive (n =746) groups. Data tabulated are counts, with estimated odds ratios (OR), with 95% confidence intervals, and p-values from Fisher's exact tests for each diagnosis method.

Diagnosis	Alive (n = 746)	Dead (n = 313)	OR (95% CI)	p-value	
	Y:N (%Y)	Y:N (%Y)			
Pre-op 4-7days					
CT thorax scan (negative for SARS-CoV-2)	10:736 (1.3%)	2:311 (0.6%)	0.47 (0.05, 2.24)	0.526	-

For peer review only

CT thorax scan (positive for SARS-CoV-2)	12:734 (1.6%)	0:313 (0%)	0.00 (0.00, 0.85)	0.023	*
Swab (negative for SARS-CoV-2)	17:729 (2.3%)	3:310 (1%)	0.42 (0.08, 1.45)	0.215	-
Swab (positive for SARS-CoV-2)	31:715 (4.2%)	8:305 (2.6%)	0.61 (0.24, 1.37)	0.283	-
Pre-op 1-3days					
CT thorax scan (negative for SARS-CoV-2)	8:738 (1.1%)	3:310 (1%)	0.89 (0.15, 3.75)	0.999	-
CT thorax scan (positive for SARS-CoV-2)	10:736 (1.3%)	3:310 (1%)	0.71 (0.13, 2.79)	0.765	-
Swab (negative for SARS-CoV-2)	41:705 (5.5%)	9:304 (2.9%)	0.51 (0.22, 1.08)	0.080	-
Swab (positive for SARS-CoV-2)	86:660 (11.5%)	15:298 (4.8%)	0.39 (0.20, 0.69)	<0.001	*
Pre-op surgery					
CT thorax scan (negative for SARS-CoV-2)	0:746 (0%)	0:313 (0%)	-	0.999	-
CT thorax scan (positive for SARS-CoV-2)	4:742 (0.5%)	0:313 (0%)	0.00 (0.00, 3.61)	0.326	-
Swab (negative for SARS-CoV-2)	13:733 (1.7%)	2:311 (0.6%)	0.36 (0.04, 1.62)	0.254	-
Swab (positive for SARS-CoV-2)	18:728 (2.4%)	7:306 (2.2%)	0.93 (0.32, 2.35)	1	-
Post-op Admission					
CT thorax scan (negative for SARS-CoV-2)	4:742 (0.5%)	1:312 (0.3%)	0.60 (0.01, 6.04)	0.999	-
CT thorax scan (positive for SARS-CoV-2)	8:738 (1.1%)	4:309 (1.3%)	1.19 (0.26, 4.50)	0.756	-
Swab (negative for SARS-CoV-2)	51:695 (6.8%)	12:301 (3.8%)	0.54 (0.26, 1.05)	0.064	-
Swab (positive for SARS-CoV-2)	317:429 (42.5%)	143:170 (45.7%)	1.14 (0.87, 1.50)	0.342	-
Discharge 30days					
CT thorax scan (negative for SARS-CoV-2)	4:742 (0.5%)	0:313 (0%)	0.00 (0.00, 3.61)	0.326	-
CT thorax scan (positive for SARS-CoV-2)	0:746 (0%)	1:312 (0.3%)	-	-	-
Swab (negative for SARS-CoV-2)	8:738 (1.1%)	0:313 (0%)	0.00 (0.00, 1.39)	0.114	-
Swab (positive for SARS-CoV-2)	75:671 (10.1%)	27:286 (8.6%)	0.85 (0.51, 1.36)	0.496	-
Diagnosis					
Positive SARS-CoV-2 swab - before surgery	122:624 (16.4%)	31:282 (9.9%)	0.56 (0.36, 0.86)	0.007	*
Positive SARS-CoV-2 swab - after surgery	409:337 (54.8%)	177:136 (56.5%)	1.07 (0.82, 1.41)	0.636	-

CT scan of the chest confirming SARS-CoV-2 - before surgery	20:726 (2.7%)	2:311 (0.6%)	0.23 (0.03, 0.97)	0.033	*
CT scan of the chest confirming SARS-CoV-2 - after surgery	9:737 (1.2%)	5:308 (1.6%)	1.33 (0.35, 4.46)	0.569	-
Clinical diagnosis or chest x-ray - suspected before time of surgery	39:707 (5.2%)	12:301 (3.8%)	0.72 (0.34, 1.43)	0.432	-
Clinical diagnosis or chest x-ray - suspected after time of surgery	67:679 (9%)	31:282 (9.9%)	1.11 (0.69, 1.77)	0.643	-

Table S4. SARS-CoV-2 symptoms data summaries by dead (n = 313) and alive (n =746) groups. Data tabulated are counts, with estimated odds ratios (OR), with 95% confidence intervals, and p-values from Fisher's exact tests for each symptom.

Symptom	Alive (n = 746)	Dead (n = 313)	OR (95% CI)	p-value	
	Y:N (%Y)	Y:N (%Y)			
Abdominal pain	11:735 (1.5%)	1:312 (0.3%)	0.21 (0.01, 1.49)	0.124	-
Breathlessness (dyspnoea)	54:692 (7.2%)	31:282 (9.9%)	1.41 (0.86, 2.28)	0.172	-
Cough	73:673 (9.8%)	35:278 (11.2%)	1.16 (0.73, 1.81)	0.505	-
Diarrhoea	8:738 (1.1%)	1:312 (0.3%)	0.30 (0.01, 2.22)	0.295	-
Fatigue	21:725 (2.8%)	10:303 (3.2%)	1.14 (0.47, 2.56)	0.695	-
Fever (>38 celsius)	61:685 (8.2%)	25:288 (8%)	0.98 (0.57, 1.61)	0.999	-
Haemoptysis	0:746 (0.0%)	0:313 (0.0%)	-	-	-
Myalgia	10:736 (1.3%)	3:310 (1%)	0.71 (0.13, 2.79)	0.765	-
Nausea/vomiting	13:733 (1.7%)	7:306 (2.2%)	1.29 (0.43, 3.52)	0.623	-
Sputum	8:738 (1.1%)	4:309 (1.3%)	1.19 (0.26, 4.50)	0.756	-
Other	311:435 (41.7%)	136:177 (43.5%)	1.08 (0.82, 1.42)	0.633	-

Table S5. Pre-surgery measures data (n, mean and sd) for the full study population (n = 1063), and dead (n = 313) and alive (n =746) groups, and the difference in means between groups, with 95% confidence interval, and p-values from unpaired t-tests.

For peer review only

Measure	Full (n = 1063)		Alive (n = 746)		Dead (n = 313)		Difference (95%CI)	p-value
	n	Mean (SD)	n	Mean (SD)	n	Mean (SD)		
Respiratory rate (breaths/minute)	996	17.75 (3.52)	706	17.65 (3.68)	289	18.02 (3.09)	-0.37 (-0.85, 0.11)	0.132
Heart rate (bpm)	1022	81.10 (14.63)	723	81.16 (14.59)	298	80.95 (14.77)	0.22 (-1.76, 2.20)	0.830
Systolic blood pressure (mmHg)	1023	138.15 (26.04)	724	138.33 (25.85)	298	137.61 (26.53)	0.72 (-2.80, 4.24)	0.687
Diastolic blood pressure (mmHg)	1021	72.97 (13.89)	723	73.07 (13.86)	297	72.70 (14.00)	0.37 (-1.52, 2.25)	0.703
Haemoglobin (g/L)	1062	117.94 (19.22)	745	118.35 (19.21)	313	116.93 (19.26)	1.41 (-1.13, 3.95)	0.276
White cell count (10^9/L)	1060	10.33 (4.26)	744	10.33 (4.36)	313	10.34 (4.04)	-0.01 (-0.57, 0.56)	0.976
C-reactive protein (mg/L)	738	54.70 (66.26)	514	54.84 (67.68)	221	54.63 (63.34)	0.21 (-10.28, 10.70)	0.969

Table S6. Operation details for the full study population (n = 1063), and dead (n = 313) and alive (n =746) groups; p-values are for Fisher's exact tests comparing groups for each characteristic.

Characteristic	Full (n = 1063)	Alive (n = 746)	Dead (n = 313)	Dead (%)
Anaesthesia (p-value = 0.787)				
General	527 (49.6%)	368	157	29.9
Regional	524 (49.3%)	372	152	29.0
Missing	12 (27.8%)	6	4	33.3
*Pre-op respiration (p-value = 0.031 *)*				
None	714 (67.2%)	520	194	27.2
Oxygen	336 (31.6%)	220	115	34.3
Ventilated	2 (0.2%)	2	0	0.0
Missing	11(1.03%)	4	4	36.4
Pre-op delay (p-value = 0.220)				

For peer review only

< 6 hours	16 (1.5%)	11	5	31.2
6-23 hours	322 (30.3%)	232	88	27.5
24-47 hours	224 (21.1%)	147	77	34.4
48-71 hours	82 (7.7%)	61	21	25.6
72+ hours	123 (11.6%)	94	29	23.6
Missing	296 (27.8%)	201	93	31.4
*Procedure (p-value = 0.015 *)*				
LIMB - lower limb - total hip replacement	45 (4.2%)	41	4	8.9
LIMB - lower limb fracture - Cannulated Screws	5 (0.5%)	5	0	0.0
LIMB - lower limb fracture - Reduction and Internal Fixation	15 (1.4%)	10	5	33.3
LIMB - lower limb fracture - Dynamic Hip Screw	276 (26%)	195	81	29.3
LIMB - lower limb fracture - Reduction and Intramedullary Fixation	243 (22.9%)	169	73	30.2
LIMB - lower limb fracture - Partial Hip Replacement (Hemiarthroplasty)	479 (45.1%)	326	150	31.5
Missing	0 (0.0%)	0	0	0.0

Figure S1. Boxplots showing distributions of pre-surgery measures by outcome status (dead or alive). Boxes show interquartile range (IQR), bars medians and whiskers are 1.5 times IQR.

For peer review only

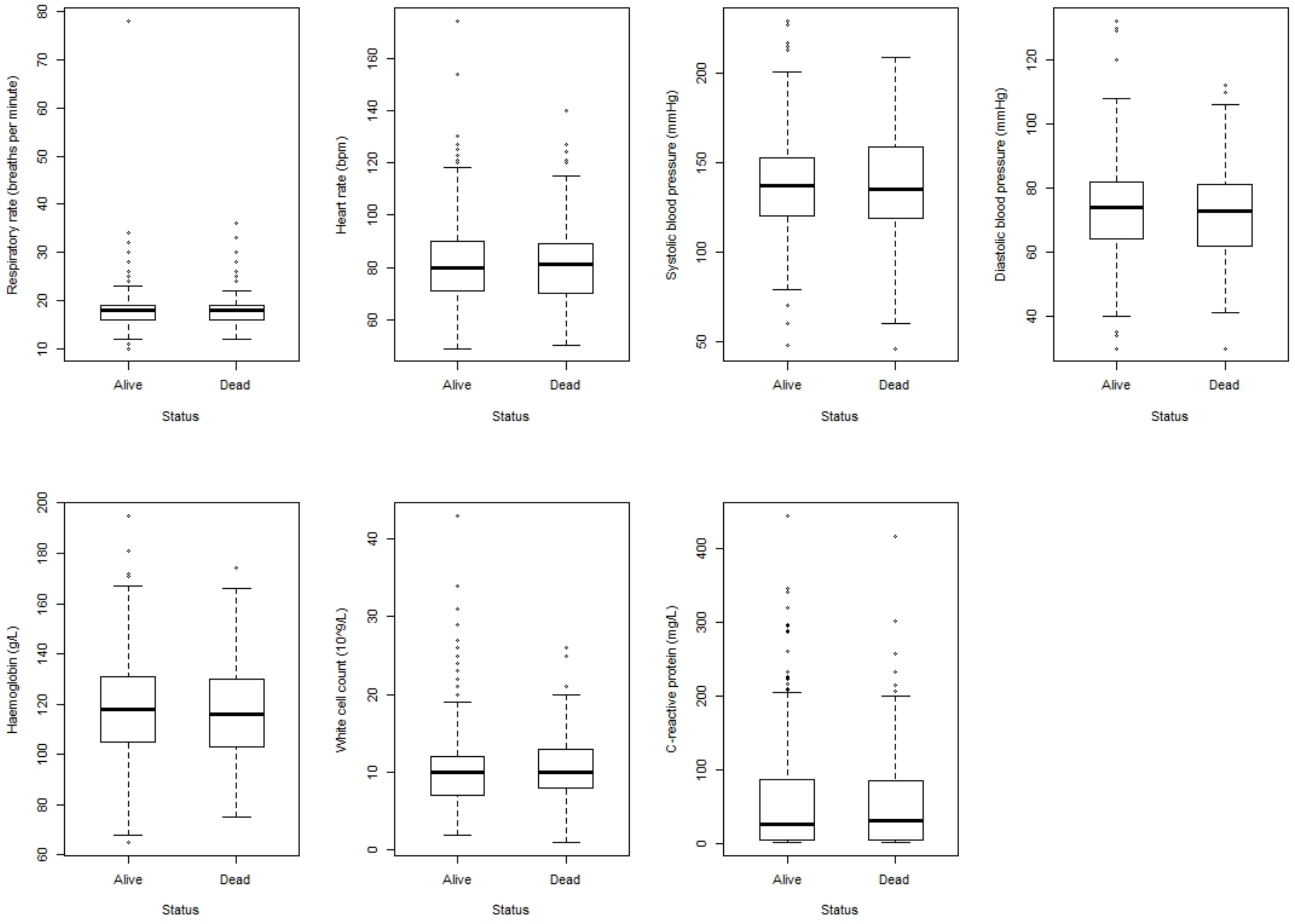
Respiratory rate (breaths per minute)
Heart rate (bpm)
Systolic blood pressure (mmHg)
Diastolic blood pressure (mmHg)
Haemoglobin (g/L)
White cell count (10^9/L)
C-reactive protein (mg/L)
Alive
Dead
Status

44

For peer review only

STROBE Statement—Checklist of items that should be included in reports of ***cohort studies***

	Item No	Recommendation	Page No
**Title and abstract**	1	(*a*) Indicate the study's design with a commonly used term in the title or the abstract<br>(*b*) Provide in the abstract an informative and balanced summary of what was done and what was found	1 & 2
**Introduction**			
Background/rationale	2	Explain the scientific background and rationale for the investigation being reported	3
Objectives	3	State specific objectives, including any prespecified hypotheses	4
**Methods**			
Study design	4	Present key elements of study design early in the paper	5
Setting	5	Describe the setting, locations, and relevant dates, including periods of recruitment, exposure, follow-up, and data collection	5
Participants	6	(*a*) Give the eligibility criteria, and the sources and methods of selection of participants. Describe methods of follow-up<br>(*b*) For matched studies, give matching criteria and number of exposed and unexposed	6
Variables	7	Clearly define all outcomes, exposures, predictors, potential confounders, and effect modifiers. Give diagnostic criteria, if applicable	7
Data sources/ measurement	8*	For each variable of interest, give sources of data and details of methods of assessment (measurement). Describe comparability of assessment methods if there is more than one group	6
Bias	9	Describe any efforts to address potential sources of bias	8
Study size	10	Explain how the study size was arrived at	N/A
Quantitative variables	11	Explain how quantitative variables were handled in the analyses. If applicable, describe which groupings were chosen and why	8
Statistical methods	12	(*a*) Describe all statistical methods, including those used to control for confounding<br>(*b*) Describe any methods used to examine subgroups and interactions<br>(*c*) Explain how missing data were addressed<br>(*d*) If applicable, explain how loss to follow-up was addressed<br>(*e*) Describe any sensitivity analyses	8
**Results**			
Participants	13*	(a) Report numbers of individuals at each stage of study—eg numbers potentially eligible, examined for eligibility, confirmed eligible, included in the study, completing follow-up, and analysed<br>(b) Give reasons for non-participation at each stage<br>(c) Consider use of a flow diagram	9
Descriptive data	14*	(a) Give characteristics of study participants (eg demographic, clinical, social) and information on exposures and potential confounders<br>(b) Indicate number of participants with missing data for each variable of interest<br>(c) Summarise follow-up time (eg, average and total amount)	9
Outcome data	15*	Report numbers of outcome events or summary measures over time	9

For peer review only - http://bmjopen.bmj.com/site/about/guidelines.xhtml

Main results	16	(*a*) Give unadjusted estimates and, if applicable, confounder-adjusted estimates and their precision (eg, 95% confidence interval). Make clear which confounders were adjusted for and why they were included<br>(*b*) Report category boundaries when continuous variables were categorized<br>(*c*) If relevant, consider translating estimates of relative risk into absolute risk for a meaningful time period	9-12
Other analyses	17	Report other analyses done—eg analyses of subgroups and interactions, and sensitivity analyses	9-12
**Discussion**			
Key results	18	Summarise key results with reference to study objectives	13
Limitations	19	Discuss limitations of the study, taking into account sources of potential bias or imprecision. Discuss both direction and magnitude of any potential bias	16
Interpretation	20	Give a cautious overall interpretation of results considering objectives, limitations, multiplicity of analyses, results from similar studies, and other relevant evidence	13-15
Generalisability	21	Discuss the generalisability (external validity) of the study results	13-15
**Other information**			
Funding	22	Give the source of funding and the role of the funders for the present study and, if applicable, for the original study on which the present article is based	1

*Give information separately for exposed and unexposed groups.

Note: An Explanation and Elaboration article discusses each checklist item and gives methodological background and published examples of transparent reporting. The STROBE checklist is best used in conjunction with this article (freely available on the Web sites of PLoS Medicine at http://www.plosmedicine.org/, Annals of Internal Medicine at http://www.annals.org/, and Epidemiology at http://www.epidem.com/). Information on the STROBE Initiative is available at http://www.strobe-statement.org.

For peer review only

For peer review only - http://bmjopen.bmj.com/site/about/guidelines.xhtml

BMJ Open

Outcomes after peri-operative SARS-CoV-2 infection in patients with proximal femoral fractures: an international cohort study

Journal:	*BMJ Open*
Manuscript ID	bmjopen-2021-050830.R1
Article Type:	Original research
Date Submitted by the Author:	24-Aug-2021
Complete List of Authors:	Khatri, Chetan; University of Warwick,
**Primary Subject Heading**:	Surgery
Secondary Subject Heading:	Anaesthesia, Evidence based practice
Keywords:	COVID-19, Hip < ORTHOPAEDIC & TRAUMA SURGERY, TRAUMA MANAGEMENT

SCHOLARONE™ Manuscripts

I, the Submitting Author has the right to grant and does grant on behalf of all authors of the Work (as defined in the below author licence), an exclusive licence and/or a non-exclusive licence for contributions from authors who are: i) UK Crown employees; ii) where BMJ has agreed a CC-BY licence shall apply, and/or iii) in accordance with the terms applicable for US Federal Government officers or employees acting as part of their official duties; on a worldwide, perpetual, irrevocable, royalty-free basis to BMJ Publishing Group Ltd ("BMJ") its licensees and where the relevant Journal is co-owned by BMJ to the co-owners of the Journal, to publish the Work in this journal and any other BMJ products and to exploit all rights, as set out in our licence.

The Submitting Author accepts and understands that any supply made under these terms is made by BMJ to the Submitting Author unless you are acting as an employee on behalf of your employer or a postgraduate student of an affiliated institution which is paying any applicable article publishing charge ("APC") for Open Access articles. Where the Submitting Author wishes to make the Work available on an Open Access basis (and intends to pay the relevant APC), the terms of reuse of such Open Access shall be governed by a Creative Commons licence – details of these licences and which Creative Commons licence will apply to this Work are set out in our licence referred to above.

Other than as permitted in any relevant BMJ Author's Self Archiving Policies, I confirm this Work has not been accepted for publication elsewhere, is not being considered for publication elsewhere and does not duplicate material already published. I confirm all authors consent to publication of this Work and authorise the granting of this licence.

For peer review only

Outcomes after peri-operative SARS-CoV-2 infection in patients with proximal femoral fractures: an international cohort study

COVIDSurg Collaborative*

***Author contributions:** Full list of authors and roles within this research project are available in Supplementary files, titled Authorship (available to editors)

Correspondence: **Chetan Khatri**
Email: Chetan.khatri@gmail.com
Twitter: @ChetanKhatri2
Address: Warwick Clinical Trials Unit
Warwick Medical School, University of Warwick
Coventry, United Kingdom
CV4 7AL

Declarations

Competing interest: The collaborative group report no conflicts of interest. Funding is disclosed below.
Word count: 3361 (excluding tables and references)
Key words: Proximal fracture, SARS-CoV-2, COVID-19, Mortality

For peer review only

ABSTRACT

Objectives: Studies have demonstrated high rates of mortality in people with proximal femoral fracture and SARS-CoV-2, but there is limited published data on the factors that influence mortality for clinicians to make informed treatment decisions. This study aims to report the 30-day mortality associated with peri-operative infection of patients undergoing surgery for proximal femoral fractures and to examine the factors that influence mortality in a multi-variate analysis.

Setting: Prospective, international, multicentre, observational cohort study.

Participants: Patients undergoing any operation for a proximal femoral fracture from 1st February to 30th April 2020 and with perioperative SARS-CoV-2 infection (either 7-days prior, or 30-days post-operative).

Primary outcome: 30-day mortality. Multivariate modelling was performed to identify factors associated with 30-day mortality.

Results: This study reports included 1063 patients from 174 hospitals in 19 countries. Overall 30-day mortality was 29.4% (313/1063). In an adjusted model, 30-day mortality was associated with male gender (OR 2.29, 95% CI 1.68-3.13, p<0.001), age >80 years (OR 1.60, 95% CI 1.1-2.31, p=0.013), pre-operative diagnosis of dementia (OR 1.57, 95% CI 1.15-2.16, p=0.005), kidney disease (OR 1.73, 95% CI 1.18-2.55, p=0.005) and congestive heart failure (OR 1.62, 95% CI 1.06-2.48, p=0.025). Mortality at 30-days was lower in patients with a pre-operative diagnosis of SARS-CoV-2 (OR 0.6, 95% CI 0.6 (0.42-0.85), p=0.004). There was no difference in mortality in patients with an increase to delay in surgery (p=0.220), or type of anaesthetic given (p=0.787).

Conclusions: Patients undergoing surgery for a proximal femoral fracture with a peri-operative infection of SARS-CoV-2 have a high rate of mortality. This study would support the need for providing these patients with individualised medical and anaesthetic care, including medical optimisation before theatre. Careful pre-operative counselling is needed for those with a proximal femoral fracture and SARS-CoV-2, especially those in the highest risk groups.

For peer review only

ARTICLE SUMMARY

Strengths and limitations of this study

- This is a large, international, multicentre cohort study from which the results are generalisable across populations in other countries.
- This study described specific risk factors for mortality, which patients and those who care for them should use to make informed decisions regarding care.
- There is not control arm to assess contemporaneous patients with undergoing an operation for proximal femoral fractures without SARS-CoV-2 infection during the height of the pandemic. However with high-quality data present pre-pandemic strongly suggests a substantial increase in mortality.

For peer review only

MAIN TEXT

Background

The rapid worldwide spread of Coronavirus Disease-2019 (COVID-19), caused by the Severe Acute Respiratory Syndrome CoronaVirus-2 (SARS-CoV-2) has had a severe effect on the elderly and frail population. A fracture of the proximal femur (neck of femur fracture) is a critical event in the elderly, frail population, with a high rate of death despite medical and surgical intervention [1]. Since 2007, there has been a steady improvement in mortality after a proximal femoral fracture with 6.1% of patients dying within 30 days of injury in the UK in 2018 [2]. However, the emergence of COVID-19 presents a new and unquantified risk to this particularly vulnerable group.

Proximal femur fractures represent a large international burden with incidence between 43 and 920 per 100,000 population [3]. As most fractures of the proximal femur happen as a result of trips or falls in the home, people have continued to present with this injury despite social restrictions [4, 5]. These patients typically have multiple co-morbidities and frailty is common [1]. Resultantly, they are particularly vulnerable to pulmonary complications [1, 6]. It is widely accepted that elderly patients with existing co-morbidities are at higher risks of critical illness and mortality due to COVID-19, potentially due to a higher preponderance to release pro-inflammatory cytokines that result in severe disease [7-9].

Clinicians have been swift to respond to this pandemic with large re-organisation of service provision [10, 11]. In response to this, the COVIDSurg collaborative

(www.globalsurg.org/covidsurg) has collected an international, large volume dataset to inform the global community of the safety of surgery in patients with peri-operative SARS-CoV-2 infection. The first report has demonstrated a 30-day mortality of 23.8% across patients undergoing any type of surgery [12]. Data published so far has reported a high mortality rate in a small cohort of patients with proximal femoral fractures positive for SARS-CoV-2 infection, with a maximum cohort size of 114 patients (range 10-114 patients) [13-20]. However few reports have the sample size sufficient to explore the factors that influence outcome. Further, large-scale data are required to explore pre-operative and operative variables that influence outcomes in order to inform the clinical decision-making processes.

Aims

The primary aim of this study is to determine the mortality rate observed in patients undergoing surgery for proximal femoral fracture with peri-operative SARS-CoV-2 infection. Secondarily, we aim to explore the patient and treatment factors associated with these outcomes.

For peer review only

Methods

Setting

This is an international, multicentre cohort study including consecutive patients who underwent surgery for proximal femoral fracture from 1st February 2020 to 30th April 2020. This study is a pre-planned sub-analysis of a larger, ongoing study designed to assess outcomes following all surgery for patients with perioperative SARS-CoV-2 infection [12].

The COVIDSurg collaborative is an international, multicentre, multidisciplinary team with individual collaborators collecting data locally, which is collated centrally. The collaborative methodology, which is well described and validated was used for this project [21]. The study protocol was registered online (ClinicalTrials.gov identifier: NCT04323644).

Inclusion Criteria

Participating hospitals included consecutive patients undergoing surgery for proximal femoral fractures that had SARS-CoV-2 infection diagnosed (laboratory, clinical or radiologically) either 7 days pre-operatively, or up to 30-days post-operatively. For those diagnosed pre-operative, this represents the timeframe where the majority of patients still active disease [22]. For those patients who underwent multiple procedures, the procedure closest to the time of confirmation of SARS-CoV-2 infection was defined as the index procedure.

For peer review only

Patients received laboratory confirmation of SARS-CoV-2 using quantitative Reverse Transcription Polymerase Chain Reaction (qRT-PCR). As qRT-PCR is not available in all participating hospitals, patients were included if their diagnosis was made by clinical or radiological findings. Clinical diagnosis was made in patients presenting with symptoms and a clinical pattern of COVID-19. These included cough, fever and/or myalgia [23]. Radiological diagnosis was made through computed tomography (CT) scanning of the thorax according to local protocols. All patients who were included solely on clinical or radiological suspicion but had a subsequent negative test were excluded from the database by individual collaborators.

Diagnosis

This study includes all patients identified as having an operation for a proximal femoral fracture. The diagnosis was established pragmatically by the local site teams according to their assessment of the fracture. The reported data was screened by a central dedicated data cleaning team, with only confirmed proximal femoral fractures included in the cohort.

Patient Identification

Researchers at participating centres screened consecutive patients undergoing surgery to ensure all patients were identified. The study was initiated in some countries after their peak of infection, and therefore retrospective identification and data collection was permitted, as long as the data collection was consecutive at that site.

To reduce selection bias, a variety of written materials were distributed to site leads to highlight possible methods of identifying patients ensuring all eligible patients were

For peer review only

included. Investigators were invited to social media groups and online teleconferences to trouble-shoot recruitment issues, share learning and ensure consistent recruitment into the wider cohort.

Outcome measures

The primary outcome measure was 30-day all-cause mortality, with the day of surgery defined as day zero. The secondary outcome measure was rate of pulmonary complications, which is a composite outcome defined previously from the Prevention of Respiratory Insufficiency after Surgical Management (PRISM) randomised controlled trial [24, 25].

Pulmonary complications were defined as pneumonia, acute respiratory distress syndrome (ARDS), and/or unexpected post-operative ventilation; these have been identified as the most frequent COVID-19-related pulmonary complications in medical patients [23]. Unexpected post-operative ventilation was defined as either (i) any episode of non-invasive ventilation, invasive ventilation, or extracorporeal membrane oxygenation after initial extubation following surgery, or (ii) unexpected failure to extubate following surgery [12].

Data collection and quality assurance

Data was collected online using the Research Electronic Data Capture (REDCap) web application [26]. Demographic variables recorded consisted of age, sex, and American Society of Anesthesiologists physical status classification (ASA). Age was collected as a categorical variable by deciles of age. ASA at the time of surgery was dichotomized to (i) grades 1-2 and (ii) grades 3-5 for the purpose of analysis, time to

For peer review only

surgery to (i) under 24 hours, (ii) 24-48 hours, and (iii) over 48 hours, and surgery to (i) hemiarthroplasty, (ii) total hip replacement, (iii) dynamic hip screw, (iv) cannulated screws and (v) intramedullary nail. The timing of SARS-CoV-2 diagnosis was recorded as either preoperative or post-operative.

Before data was entered into analysis, site principle investigators were required to confirm all consecutive eligible cases had been completed and uploaded. Where diagnosis was unclear, authors were contacted for clarification.

Statistical analysis

The study was reported according to STROBE (Strengthening the Reporting of Observational Studies in Epidemiology) guidelines [27]. Proportions are expressed with 95% confidence intervals and the mean and 95% confidence intervals were used where data were assumed to be approximately normally distributed. Fishers exact test was used for categorical data. Non-parametric data was summarised with the median and interquartile ranges. Statistical significance was assessed at the 5% level.

The risk of death at 30 days was chosen as the primary outcome for the study. Mixed-effects logistic regression analysis was used to assess the strength and significance of associations between a number of explanatory variables and death within 30-days. Random effects were included in the mixed-effects model to account for the hierarchical structure of the data (individual hospital effects are naturally nested within country effects) and fixed effects were included to adjust for a range of pre-operative variables that may influence mortality in this population, and relevant factors related to the injury or treatment (e.g. type of operation, time from admission

For peer review only

to operation, type of anaesthetic). An additional analysis of the same factors was undertaken using the same model structure for the secondary outcome of pulmonary complications. This was an exploratory analysis with the significance level set at 5%, with no specific adjustments made for model testing. All analyses were implemented in R (R Core Team (2020). R: A language and environment for statistical computing. R Foundation for Statistical Computing, Vienna, Austria. URL https://www.R-project.org/.).

Patient and Public Involvement

Patients were not involved in the design, conduct or reporting of this study.

For peer review only

Results

Population

This study returned 30-day follow up for 1063 patients with proximal femoral fractures. Data was collected in 174 hospitals from 19 countries (Supplementary Table S1). Of these, 65.5% were female (696/1063). 7.8% (83/1063) patients were <70 years old, 17.8% (189/1063) were between 70-79 years, 47.7% (507/1063) were between 80-89 and 26.7% (284/1063) were 90+ years old.

Mortality

Overall 30-day mortality was 29.4% (313/1063). With each decile of age, mortality significantly increased, being highest in those patients >90 years old (38.7% [110/284], p=0.001).

In an adjusted model (Figure 1), 30-day mortality was associated with male gender (OR 2.29, 95% CI 1.68-3.13, p<0.001), age >80 years (OR 1.60, 95% CI 1.1-2.31, p=0.013), diagnosis of dementia (OR 1.57, 95% CI 1.15-2.16, p=0.005), chronic kidney disease (OR 1.73, 95% CI 1.18-2.55, p=0.005) and congestive heart failure (OR 1.62, 95% CI 1.06-2.48, p=0.025). 30-day mortality was lower in patients with a pre-operative diagnosis of SARS-CoV-2 (OR 0.60, 95% CI 0.42-0.85, p=0.004). Non-adjusted values are presented in Table S2.

Pulmonary complications

In an adjusted model (Figure 2), respiratory complications were associated with male gender (OR 1.7, 95% CI 1.27-2.28, p<0.001), diagnosis of dementia (OR 1.34, 95%

For peer review only

CI 1.01-1.79, p=0.044) and congestive heart failure (OR 1.76, 95% CI 1.17-2.63, p=0.006). The presence of Chronic Obstructive Pulmonary Disorder (COPD) showed no significant association (OR 1.42, 95% CI 0.96-2.09, p=0.076).

Diagnosis

The majority of diagnosis of SARS-CoV-2 was made via PCR swab testing 93.3% (992/1063) (Table S1 & S3) and there was no difference in mortality between those diagnosed clinically (p=0.668). The majority of patients received a diagnosis post-operatively 69% (733/1063).

Pre-operative variables

Pre-operative symptoms (Table S4), including breathlessness, cough and fever (>38 ° Celsius) were not significantly different in patients who were alive or dead at 30 days post-operatively. On examination of pre-operative observations, a high respiratory rate was predictive of mortality (OR 1.73 95% CI 1.18-2.55, p=0.025) (Figure 1). However, there was no significant difference in patient's heart rate, systolic or diastolic blood pressure (Table S5, Figure S1) between those who were alive or dead at 30 days.

Those patients with ASA grade 3-5 had a significantly higher mortality of 31.4% (281/899) versus ASA of 1-2 of 18.5% (28/151), p=0.001.

Procedures

The operations were carried out under a general anaesthetic in 49.6% (527/1063) of patients (Table S6). 67.2% (714/1063) of patients did not require any pre-operative

For peer review only

oxygen therapy. In this cohort, 31.8% (338/1063) of patients had their operation within 24 hours of presentation to hospital, 21.1% (224/1063) had their operation between 24-47 hours and 19.2% (205/1063) of patients had their operation after 48 hours of presentation to hospital.

In this cohort, 45.1% (479/1063) of patients underwent hemiarthroplasty with a further 4.2% undergoing total hip replacement (45/1063). For patients who underwent fixation, 26% (276/1063) underwent Dynamic Hip Screw (DHS) fixation, 22.9% (243/1063) patients underwent intramedullary fixation, 0.5% (5/1063) underwent cannulated screw fixation whilst a further 1.4% (15/1053) underwent internal fixation.

There was no difference in mortality between patients undergoing general and regional anaesthesia (29.9% [157/527 versus 29.0% [152/524], p=0.787). However, there was an increased mortality in those patients requiring pre-operative oxygen therapy (34.3% [115/336] versus 27.2% [194/714], p=0.031).

There was no significant difference in mortality for patients with delayed operation. The highest mortality was for patients operated between 24-47 hours of admission (34.4%, [77/224]) but was not significantly higher than less than those operated after 48 hours (p=0.220).

Mortality was highest in March (33.7%, 159/474) compared to April (27.0%, 150/558), and February (11.5%, 3/26), p=0.007 (Table S1).

For peer review only

For peer review only - http://bmjopen.bmj.com/site/about/guidelines.xhtml

Discussion

The 30-day mortality rate for patients with a peri-operative diagnosis of SARS-CoV-2 infection undergoing surgery for proximal femoral fracture is substantial. An overall rate of 29.4% compares to the reported 30-day mortality in the literature for proximal femoral fractures ranging between 3.5-6.8% [2, 28-32]. This rate is higher than found at the one year time point [33]. Further, elderly patients, and those with medical comorbidities such as dementia, chronic kidney disease and congestive heart failure were associated with higher risk of 30-day mortality. Notably, patients with a pre-operative diagnosis of SARS-CoV-2 infection had lower rates of 30-day mortality, likely reflecting early recognition and closer management of these patients. Findings from this study will be useful in guiding clinicians to identify high-risk patients that may warrant closer medical and surgical input during the COVID-19 pandemic.

Considering this high mortality, it is critical that patients who present without a diagnosis SARS-CoV-2 with proximal femoral fractures are protected from contracting SARS-CoV-2 in the peri-operative period. A study by Kayani et al. has suggested that half of infections in patients with proximal femoral fractures occur in hospital, as denoted by having negative pre-operative samples [17]. Similarly, a study by Hall et al. has suggested nearly half of cases were due to nosocomial transmission [34]. Within this study, 733 (69%) of infections were diagnosed post-operatively. This may infer that infections have been transferred in hospital, although due to incubation period of the virus, it is hard to know the proportion that contracted the virus prior to presentation or in hospital. [7, 35] Higher mortality was observed in people who had a post-operative diagnosis, which emphasises the critical importance

For peer review only

of avoiding in-hospital transmission. Hospitals should consider implementation of careful infection control processes to minimise and prevent transmission of SARS-CoV-2 infection. Within the elective setting, the creation of COVID-19-free surgical pathways for elective patients has been shown to reduce infection and subsequent mortality [36-38] and whilst only some of the principles are transferrable to the emergency setting, it demonstrates the value of meticulous infection control processes throughout the hospital stay. Furthermore, patients should be reinforced of methods to reduce risk of transmission in the community after discharge, including (but not limited to) social distancing, isolation and hygiene.

For those patients presenting with SARS-CoV-2 (either existing diagnosis or clinical findings suggestive of) and a proximal femoral fracture, it is important for data to be used as part of the informed consent process. In patients with multiple high-risk factors such as those who are more elderly, have respiratory and cardiac co-morbidities, non-operative management may be considered following an appropriate discussion with the patient and/or their family. Every year in the UK, 2.5% of hip fractures are treated non-operatively [39]. A study performed before the pandemic reported that the mortality within thirty days for conservatively treated patients was 31.3% [40]. We do not know the mortality from non-operative management during the pandemic for patients with SARS-Cov-2, but the particularly high mortality associated with surgery in high-risk groups may change the balance of benefit and harm towards conservative treatment and this should be considered.

The 30-day mortality of 29.4% identified within this study is comparable to published literature, in the UK (range from 16.3%-35.6%) [15-17, 19, 34, 41], Italy (18.75%)

For peer review only

[14], Spain (30.4%) [13] and the USA (range from 35.3%-56%) [18, 20]. From a study within the UK, the authors also found a correlation between male sex and increased mortality (OR 2.69), which is similar to that demonstrated in this study (OR 2.29) [16]. Additionally, another UK study reported having more than three co-morbidities as a risk factor for mortality [17]. This study has specifically delineated a diagnosis of dementia, chronic kidney disease and congestive heart failure as being independent risk factors for mortality. In a study from USA, the authors found those patients who died were older with multiple co-morbidities and this was reflected in statistically significant higher ASA scores in comparison to their negative counterparts [20].

This study found that there was no significant increase in mortality with delay to surgery. Current guidelines suggest early surgery should be undertaken [42] and this is associated with lower mortality [43]. This would suggest that those patients at the highest risk of mortality can have medical optimisation, if appropriate, and will not result in a higher mortality from SARS-CoV-2 infection. This includes correction of concurrent medical issues often found in this population, examples of which include correction of acute renal failure, electrolyte disturbances and/or anticoagulation related issues. With regards to recovery from SARS-CoV-2 infection, it is important to consider that an increased risk of mortality for those undergoing surgery persists until seven weeks after diagnosis [44]. This risk reduces gradually after two weeks after diagnosis and should be considered.

Similarly, previous studies have found a higher rate of mortality in patients undergoing general versus regional anaesthesia for proximal femoral fractures [45,

For peer review only

46]. This study reports no difference between general and regional anaesthetic (29.9% versus 29.0%, p=0.787). Whilst this was not the primary outcome of this study, this suggests that a positive test for SARS-CoV-2 should not have a large influence on anaesthetic decisions. This should be interpreted with caution in the light of this being an exploratory study. Out of all clinical features, respiratory rate at presentation was associated with higher mortality. Clinicians should focus on this as an important finding when counselling patients of their peri-operative mortality.

This study has also found an increased mortality during the month of March 2020. This corresponds to the peak of caseload of infections internationally [47, 48]. Increased circulation of SARS-CoV-2 within countries has shown to increase mortality through higher viral loads [47, 49]. This study validates that surgical patients are particularly susceptible during surge of cases.

This is a large, varied cohort of patients undergoing surgery for a proximal femoral fractures with SARS-Cov-2 infection diagnosed peri-operatively. This study was conducted in multiple centres, internationally, allowing it to be generalisable across populations in other countries.

Limitations

This study was conducted in hospitals in the early to mid-phase of the pandemic where routine testing was not available in all participating centres. As such, to be pragmatic, patients were included if a clinical diagnosis was made by the treating physician. Protocols were not standardised for clinical diagnosis and were left the senior treating physician. Laboratory diagnosis was made by qRT-PCR, from which

For peer review only

false-negative results may have excluded patients from analysis. Indeed, the sensitivity of qRT-PCR testing for has shown to be as low as 32% for throat swabs [50]. However, in patients with negative results and high clinical suspicion of SARS-CoV-2 infection, multiple samples are often taken, including broncho-alveolar lavage. Thus, the number of patients excluded is expected to be low. Whilst this study reports a higher mortality from post-operative diagnosis of SARS-CoV-2 infection, it is unclear whether the infection was contracted pre-operatively or not, as has been discussed above.

This study does not have a control arm, assessing contemporaneous patients with undergoing an operation for proximal femoral fractures without SARS-CoV-2 infection during the height of the pandemic. However, comparison with high-quality pre-pandemic data strongly suggests a substantial increase in mortality. Patients and those who care for them should consider this carefully when making decisions in this common and challenging clinical scenario.

Conclusion

Patients undergoing surgery for a proximal femoral fracture with a peri-operative infection of SARS-CoV-2 have a high rate of mortality. The study would support the approach of providing these patients with individualised medical and anaesthetic care, including medical optimisation before theatre. It is imperative to prevent transmission of coronavirus in the hospital setting. Careful pre-operative counselling is needed for those with a proximal femoral fracture and SARS-CoV-2, especially those in the highest risk groups.

For peer review only

Tables & Figures

Fig. 1 Mixed-effects logistic regression model for 30 day mortality

Fig. 2. Mixed-effects logistic regression model for pulmonary complications

Competing interests

The collaborative group report no conflicts of interest

Contributor statement

CK is the lead author for this manuscript and was responsible for inception, analysis and writing of this manuscript alongside the COVIDSurg Collaborative.

Full list of authors and roles within this research project are available in Supplementary files, titled Authorship (available to editors)

Funding

This work was supported by a National Institute for Health Research (NIHR) Global Health Research Unit Grant (NIHR 16.136.79), the Association of Coloproctology of Great Britain and Ireland, Association of Upper Gastrointestinal Surgeons, Bowel Disease Research Foundation, Yorkshire Cancer Research, Sarcoma UK, the British Association of Surgical Oncology, the Vascular Society for Great Britain and Ireland, and the European Society of Coloproctology. The funders had no role in study design, data collection, analysis and interpretation, or writing of this report. The views expressed are those of the authors and not necessarily those of the National Health Service, the NIHR, or the UK Department of Health and Social Care

Data Sharing

No additional data available. Requests for raw data can be requested via the corresponding author.

Ethics Statement

This observational study collected anonymised routine clinical data, using an established international trainee collaborative model [51]. Within the United Kingdom this was registered as a clinical audit or service evaluation at each participating trust following individual hospital policies and procedures, prior to initiating data collection at that site. In other countries, the principal investigator was responsible for obtaining local approval in line with local and/or national guidelines. In some participating countries, informed patient consent was taken, whilst in others the requirement was waived by local research committees. The lead centre for this manuscript was University Hospitals Coventry & Warwickshire (approval number SE0233). Country-specific guidelines for site set-up were published on a dedicated study website (www.globalsurg.org/covidsurg).

For peer review only

References

1. Kistler, E.A., et al., *Frailty and short-term outcomes in patients with hip fracture.* Geriatric orthopaedic surgery & rehabilitation, 2015. **6**(3): p. 209-214.
2. National Hip Fracture Database., *National Hip Fracture Database (NHFD) Annual Report 2019.* 2019.
3. Dhanwal, D.K., et al., *Epidemiology of hip fracture: worldwide geographic variation.* Indian journal of orthopaedics, 2011. **45**: p. 15-22.
4. Hampton, M., et al., *The effects of a UK lockdown on orthopaedic trauma admissions and surgical cases: A multicentre comparative study.* Bone & Joint Open, 2020. **1**(5): p. 137-143.
5. Scott, C.E., et al., *Population mobility and adult orthopaedic trauma services during the COVID-19 pandemic: fragility fracture provision remains a priority.* Bone & Joint Open, 2020. **1**(6): p. 182-189.
6. Lawrence, V.A., et al., *Medical complications and outcomes after hip fracture repair.* Archives of internal medicine, 2002. **162**(18): p. 2053-2057.
7. Huang, C., et al., *Clinical features of patients infected with 2019 novel coronavirus in Wuhan, China.* Lancet, 2020. **395**(10223): p. 497-506.
8. Besnier, E., J.J. Tuech, and L. Schwarz, *We Asked the Experts: Covid-19 Outbreak: Is There Still a Place for Scheduled Surgery? "Reflection from Pathophysiological Data".* World J Surg, 2020. **44**(6): p. 1695-1698.
9. Meftahi, G.H., et al., *The possible pathophysiology mechanism of cytokine storm in elderly adults with COVID-19 infection: the contribution of "inflame-aging".* Inflammation Research, 2020: p. 1-15.
10. Chang Liang, Z., et al., *Novel Coronavirus and Orthopaedic Surgery: Early Experiences from Singapore.* J Bone Joint Surg Am, 2020.
11. Thornton, J., *Clinicians are leading service reconfiguration to cope with covid-19.* Bmj, 2020. **369**: p. m1444.
12. COVIDSurg Collaborative., *Mortality and pulmonary complications in patients undergoing surgery with perioperative SARS-CoV-2 infection: an international cohort study.* The Lancet, 2020.
13. Munoz Vives, J.M., et al., *Mortality Rates of Patients with Proximal Femoral Fracture in a Worldwide Pandemic: Preliminary Results of the Spanish HIP-COVID Observational Study.* J Bone Joint Surg Am, 2020.
14. Catellani, F., et al., *Treatment of Proximal Femoral Fragility Fractures in Patients with COVID-19 During the SARS-CoV-2 Outbreak in Northern Italy.* J Bone Joint Surg Am, 2020.
15. Mamarelis, G., et al., *Mortality in Patients with Proximal Femoral Fracture During the COVID-19 Pandemic: A UK Hospital's Experience.* JBJS Open Access, 2020. **5**(4).
16. Rasidovic, D., et al., *Impact of COVID-19 on clinical outcomes for patients with fractured hip: a multicentre observational cohort study.* Bone & Joint Open, 2020. **1**(11): p. 697-705.
17. Kayani, B., et al., *The effects of COVID-19 on perioperative morbidity and mortality in patients with hip fractures.* Bone Joint J, 2020. **102-b**(9): p. 1136-1145.
18. Egol, K.A., et al., *Increased Mortality and Major Complications in Hip Fracture Care During the COVID-19 Pandemic: A New York City Perspective.* J Orthop Trauma, 2020. **34**(8): p. 395-402.
19. Thakrar, A., et al., *Thirty-Day Mortality Rate of Patients With Hip Fractures During the COVID-19 Pandemic: A Single Centre Prospective Study in the United Kingdom.* J Orthop Trauma, 2020. **34**(9): p. e325-e329.
20. LeBrun, D.G., et al., *Hip fracture outcomes during the COVID-19 pandemic: early results from New York.* Journal of orthopaedic trauma, 2020.

For peer review only

21. Bhangu, A., et al., *Surgical research collaboratives in the UK.* Lancet, 2013. **382**(9898): p. 1091-2.
22. Cevik, M., et al., *Virology, transmission, and pathogenesis of SARS-CoV-2.* BMJ, 2020. **371**.
23. Zhou, F., et al., *Clinical course and risk factors for mortality of adult inpatients with COVID-19 in Wuhan, China: a retrospective cohort study.* Lancet, 2020. **395**(10229): p. 1054-1062.
24. Abbott, T.E.F., et al., *A systematic review and consensus definitions for standardised end-points in perioperative medicine: pulmonary complications.* Br J Anaesth, 2018. **120**(5): p. 1066-1079.
25. Pearse, R.M., et al., *The Prevention of Respiratory Insufficiency after Surgical Management (PRISM) Trial. Report of the protocol for a pragmatic randomized controlled trial of CPAP to prevent respiratory complications and improve survival following major abdominal surgery.* Minerva Anestesiol, 2017. **83**(2): p. 175-182.
26. Harris, P.A., et al., *Research electronic data capture (REDCap)—a metadata-driven methodology and workflow process for providing translational research informatics support.* Journal of biomedical informatics, 2009. **42**(2): p. 377-381.
27. Gharaibeh, A., S. Koppikar, and F.J. Bonilla-Escobar, *Strengthening the Reporting of Observational Studies in Epidemiology (STROBE) in the International Journal of Medical Students.* International Journal of Medical Students, 2014. **2**(2): p. 36-37.
28. Blanco, J.F., et al., *30-day mortality after hip fracture surgery: Influence of postoperative factors.* PloS One, 2021. **16**(2): p. e0246963.
29. Carretta, E., et al., *Hip fracture: effectiveness of early surgery to prevent 30-day mortality.* International orthopaedics, 2011. **35**(3): p. 419-424.
30. Giannoulis, D., G.M. Calori, and P.V. Giannoudis, *Thirty-day mortality after hip fractures: has anything changed?* European Journal of Orthopaedic Surgery & Traumatology, 2016. **26**(4): p. 365-370.
31. Brauer, C.A., et al., *Incidence and mortality of hip fractures in the United States.* Jama, 2009. **302**(14): p. 1573-1579.
32. Miller, B.J., X. Cai, and P. Cram, *Mortality rates are similar after hip fractures for rural and urban patients.* Clinical Orthopaedics and Related Research, 2012. **470**(6): p. 1763-1770.
33. Mundi, S., et al., *Similar mortality rates in hip fracture patients over the past 31 years: A systematic review of RCTs.* Acta orthopaedica, 2014. **85**(1): p. 54-59.
34. Hall, A.J., et al., *IMPACT-Scot 2 report on COVID-19 in hip fracture patients: a nationwide study of mortality, risk factors for community and hospital acquired COVID-19, and suggested care pathways.* The Bone & Joint Journal, 2021. **103**: p. 1-10.
35. Phua, J., et al., *Intensive care management of coronavirus disease 2019 (COVID-19): challenges and recommendations.* Lancet Respir Med, 2020. **8**(5): p. 506-517.
36. COVIDSurg Collaborative., *Preoperative nasopharyngeal swab testing and postoperative pulmonary complications in patients undergoing elective surgery during the SARS-CoV-2 pandemic.* The British Journal of Surgery, 2020.
37. Chui, K., A. Thakrar, and S. Shankar, *Evaluating the efficacy of a two-site ('COVID-19'and 'COVID-19-free') trauma and orthopaedic service for the management of hip fractures during the COVID-19 pandemic in the UK.* Bone & Joint Open, 2020. **1**(6): p. 190-197.
38. COVIDSurg Collaborative., *Elective cancer surgery in COVID-19–free surgical pathways during the SARS-CoV-2 pandemic: an international, multicenter, comparative cohort study.* Journal of Clinical Oncology, 2020: p. JCO. 20.01933.
39. National Hip Fracture Database. *National Hip Fracture Report.* 2020; Available from: https://www.nhfd.co.uk/20/NHFDcharts.nsf/vwcharts/Surgery?opendocument .

For peer review only - http://bmjopen.bmj.com/site/about/guidelines.xhtml

40. Moulton, L., et al., *Outcome after conservatively managed intracapsular fractures of the femoral neck.* The Annals of The Royal College of Surgeons of England, 2015. **97**(4): p. 279-282.

41. Mahmood, A., et al., *Coronavirus infection in hip fractures (CHIP) study.* The Bone & Joint Journal, 2021: p. 1-5.

42. Hoffenberg, R., et al., *Fractured neck of femur: Prevention and management. Summary and recommendations of a report of the Royal College of Physicians.* Journal of the Royal College of Physicians of London, 1989. **23**(1): p. 8-14.

43. Rogers, F.B., S.R. Shackford, and M.S. Keller, *Early fixation reduces morbidity and mortality in elderly patients with hip fractures from low-impact falls.* Journal of Trauma and Acute Care Surgery, 1995. **39**(2): p. 261-265.

44. COVIDSurg. and Globalsurg Collaborative, *Timing of surgery following SARS-CoV-2 infection: an international prospective cohort study.* Anaesthesia, 2021. **76**(6): p. 748-758.

45. Ahn, E.J., et al., *Comparison of general anaesthesia and regional anaesthesia in terms of mortality and complications in elderly patients with hip fracture: a nationwide population-based study.* BMJ open, 2019. **9**(9): p. e029245.

46. Van Waesberghe, J., et al., *General vs. neuraxial anaesthesia in hip fracture patients: a systematic review and meta-analysis.* BMC anesthesiology, 2017. **17**(1): p. 87.

47. Ferguson, N., et al., *Report 9: Impact of non-pharmaceutical interventions (NPIs) to reduce COVID19 mortality and healthcare demand.* 2020.

48. University, J.H. *Coronavirus Resource Centre*. 2021; Available from: https://coronavirus.jhu.edu/data.

49. Fajnzylber, J., et al., *SARS-CoV-2 viral load is associated with increased disease severity and mortality.* Nature communications, 2020. **11**(1): p. 1-9.

50. Wang, W., et al., *Detection of SARS-CoV-2 in Different Types of Clinical Specimens.* Jama, 2020. **323**(18): p. 1843-1844.

51. COVIDSurg Collaborative, *COVIDSurg Protocol*. 2020.

For peer review only

COVIDsurg authorship list

Operations Committee: Kwabena Siaw-Acheampong, Leah Argus, Daoud Chaudhry, Brett E Dawson, James C Glasbey, Rohan R Gujjuri, Conor S Jones, Sivesh K Kamarajah, Chetan Khatri, James M Keatley, Samuel Lawday, Elizabeth Li, Harvinder Mann, Ella J Marson, Kenneth A Mclean, Maria Picciochi, Elliott H Taylor, Abhinav Tiwari, Joana FF Simoes, Isobel M Trout, Mary L Venn, Richard JW Wilkin, Aneel Bhangu*, Dmitri Nepogodiev*
**Co-chairs*

Dissemination Committee: Irida Dajti (Albania), Arben Gjata (Albania), Luis Boccalatte (Argentina), Maria Marta Modolo (Argentina), Daniel Cox (Australia), Peter Pockney (Australia), Philip Townend (Australia), Felix Aigner (Austria), Irmgard Kronberger (Austria), Kamral Hossain (Bangladesh), Gabrielle VanRamshorst (Belgium), Ismail Lawani (Benin), Gustavo Ataide (Brazil), Glauco Baiocchi (Brazil), Igor Buarque (Brazil), Muhammad Gohar (Bulgaria), Mihail Slavchev (Bulgaria), Arnav Agarwal (Canada), Amanpreet Brar (Canada), Janet Martin (Canada), Maria Marta Modolo (Chile), Maricarmen Olivos (Chile), Jose Calvache (Colombia), Carlos Jose Perez Rivera (Colombia), Ana Danic Hadzibegovic (Croatia), Tomislav Kopjar (Croatia), Jakov Mihanovic (Croatia), Jaroslav Klat (Czech Republic), René Novysedlak (Czech Republic), Peter Christensen (Denmark), Alaa El-Hussuna (Denmark), Sylvia Batista (Dominican Republic), Eddy Lincango (Ecuador), Sameh H Emile (Egypt), Mengistu Gebreyohanes Mengesha (Ethiopia), Dr.Samuel Hailu (Ethiopia), Hailu Tamiru (Ethiopia), Joonas Kauppila (Finland), Alexis Arnaud (France), Markus Albertsmeiers (Germany), Hans Lederhuber (Germany), Markus Loffler (Germany), Stephen Tabiri (Ghana), Symeon Metallidis (Greece), Georgios Tsoulfas (Greece), Maria Aguilera Lorena (Guatemala), Gustavo Grecinos (Guatemala), Tamas Mersich (Hungary), Daniel Wettstein (Hungary), Dhruv Ghosh (India), Gabriele Kembuan (Indonesia), Peiman Brouk (Iran), Mohammad Khosravi (Iran), Masoud Mozafari (Iran), Ahmed Adil (Iraq), Helen M Mohan (Ireland), Oded Zmora (Israel), Marco Fiore (Italy), Gaetano Gallo (Italy), Francesco Pata (Italy), Gianluca Pellino (Italy), Sohei Satoi (Japan), Faris Ayasra (Jordan), Mohammad Chaar (Jordan), Ildar R Fakhradiyev (Kazakhstan), Mohammad Jamal (Kuwait), Muhammed Elhadi (Libya), Aiste Gulla (Lithuania), April Roslani (Malaysia), Laura Martinez (Mexico), Antonio Ramos De La Medina (Mexico), Oumaima Outani (Morocco), Pascal Jonker (Netherlands), Schelto Kruijff (Netherlands), Milou Noltes (Netherlands), Pieter Steinkamp (Netherlands), Willemijn van der Plas (Netherlands), Adesoji Ademuyiwa (Nigeria), Babatunde Osinaike (Nigeria), Justina Seyi-olajide (Nigeria), Emmanuel Williams (Nigeria), Sofija Pejkova (North Macedonia), Knut Magne Augestad (Norway), Kjetil Soreide (Norway), Zainab Al Balushi (Oman), Ahmad Qureshi (Pakistan), Raza Sayyed (Pakistan), Mustafa Abu Mohsen Daraghmeh (Palestine), Sadi Abukhalaf (Palestine), Moises Cukier (Panama), Hugo Gomez (Paraguay), Sebastian Shu (Peru), Ximena Vasquez (Peru), Marie Dione Parreno-Sacdalan (Philippines), Piotr Major (Poland), José Azevedo (Portugal), Miguel Cunha (Portugal), Irene Santos (Portugal), Ahmad Zarour (Qatar), Eduard-Alexandru Bonci (Romania), Ionut Negoi (Romania), Sergey Efetov (Russia), Andrey Litvin (Russia), Faustin Ntirenganya (Rwanda), Ehab AlAmeer (Saudi Arabia), Dejan Radenkovic (Serbia), Frederick Koh Hong Xiang (Singapore), Chew Min Hoe (Singapore), James Ngu Chi Yong (Singapore), Rachel Moore (South Africa), Ncamsile Nhlabathi (South Africa), Ruth Blanco Colino (Spain), Ana Minaya Bravo (Spain), Ana Minaya-Bravo (Spain), Umesh Jayarajah (Sri Lanka), Dakshitha Wickramasinghe (Sri Lanka), Mohammed Elmujtaba (Sudan), William Jebril (Sweden), Martin Rutegård (Sweden), Malin Sund (Sweden), Arda Isik (Turkey), Sezai Leventoğlu (Turkey), Tom EF Abbott (UK), Ruth Benson (UK), Ed Caruna (UK), Sohini Chakrabortee (UK), Andreas Demetriades (UK), Anant Desai (UK), Thomas D Drake (UK), John G Edwards (UK), Jonathan P Evans (UK), Samuel Ford (UK), Christina Fotopoulou (UK), Ewen Griffiths (UK), Peter Hutchinson (UK), Michael D Jenkinson (UK), Tabassum Khan (UK), Stephen Knight (UK), Angelos Kolias (UK), Elaine Leung (UK), Siobhan McKay (UK), Lisa Norman (UK), Riinu Ots (UK), Vidya Raghavan (UK), Keith Roberts (UK), Andrew Schache (UK), Richard Shaw (UK), Katie Shaw (UK), Neil Smart (UK), Grant Stewart (UK), Sudha Sundar (UK), Dale Vimalchandran (UK), Naomi Wright (UK), Sattar Alshryda (United Arab Emirates), Osaid Alser (United States), Kerry Breen (United States), Ian Ganly (United States), Haytham Kaafarani (United States), Brittany Kendall (United States), Hassan Mashbari (United States), Hamza Al Naggar (Yemen), Dennis Mazingi (Zimbabwe), EuroSurg, European Society of Coloproctology (ESCP), GlobalSurg, GlobalPaedSurg, ItSURG, PTSurg, SpainSurg, Italian Society of Colorectal Surgery (SICCR), Association of Surgeons in Training (ASiT), Irish Surgical Research Collaborative (ISRC), Joana FF Simoes (Chair).

Collaborators (*asterix indicates principle investigator): Dajti I (University Hospital Center Nene Tereza, Albania); Valenzuela JI* (Hospital Velez Sarsfield, Argentina); Boccalatte LA*, Gemelli NA, Smith

DE (Hospital Italiano De Buenos Aires, Argentina); Dudi-Venkata NN, Kroon HM*, Sammour T (Royal Adelaide Hospital, Australia); Roberts M*, Mitchell D*, Lah K*, Pearce A, Morton A (Royal Brisbane and Women's Hospital, Australia); Dawson AC*, Drane A (Gosford Hospital, Australia); Sharpin C*, Nataraja RM*, Pacilli M (Monash Childrens Hospital, Australia); Cox DRA, Muralidharan V*, Riddiough GE, Clarke EM, Jamel W, Qin KR (Austin Hospital, Australia); Pockney P*, Cope D, Egoroff N, Lott N (John Hunter Hospital, Australia); Putnis S, De Robles S, Ang Z (Wollongong Public Hospital, Wollongong, Australia); Mitteregger M, Uranitsch S, Stiegler M, Seitinger G, Aigner F* (Krankenhaus der Barmherzigen Brüder Graz, Austria); Lumenta DB*, Nischwitz SP, Richtig E, Pau M, Srekl-Filzmaier P, Eibinger N, Michelitsch B, Fediuk M, Papinutti A, Seidel G, Kahn J, Cohnert TU (Medical University of Graz, Austria); Messner F, Öfner D* (Medical University of Innsbruck, Austria); Presl J*, Varga M, Weitzendorfer M, Emmanuel K (Paracelsus Medical University, Salzburg, Austria); Binder AD (Universitätsklinikum Tulln, Tulln, Austria); Zimmermann M, Holawe S, Nkenke E, Grimm C, Kranawetter M (Medical University of Vienna, Vienna, Austria); Rahman Mitul A*, Islam N, Karim S (Dhaka Shishu (Children) Hospital, Bangladesh); Komen N*, Putnis S, De Robles S, Ang E (University Hospital Antwerp (UZA), Edegem, Belgium); De Praetere H*, Tollens T, Schols G , Smets C, Haenen L (Imelda Hospital, Bonheiden, Belgium); Quintens J, Van Belle K* (Europe Hospitals, Belgium); Van Ramshorst GH*, Pattyn P, Desender L, Martens T, Van de putte D (Ghent University Hospital, Belgium); Lerut P*, Grimonprez A, Janssen M, De Smul G, Wallaert P (AZ Groeninge, Belgium); Van den Eynde J, Oosterlinck W*, Van den Eynde R, Sermon A, Boeckxstaens A, Cordonnier A, De Coster J, Jaekers J, Politis C, Miserez M (UZ Leuven, Belgium); Duchateau N, De Gheldere C* (Heilig Hart Ziekenhuis Lier, Belgium); Flamey N, Pattyn P* (AZ Delta, Belgium); Christiano A*, Guidi B, Minussi AL, Castro S, Okoba W, Maldonado FHR, Oliveira P, Baldasso T, Santos L (Fundação Centro Médico De Campinas, Brazil); Gomes GMA, Buarque IL, Pol-Fachin L, Bezerra TS, Barros AV*, da Silva AMR, Leite ALS, Silvestre DWA, Ferro CC (Hospital Santa Casa De Misericórdia De Maceió, Brazil); Araujo MS*, Lopes LM, Damasceno PD, Araujo DHS (Hospital Universitário Antonio Pedro, Brazil); Laporte G*, Salem MC (Irmandade Da Santa Casa De Misericórdia De Porto Alegre, Brazil); Guimaraes-Filho, MAC (Pedro Ernesto University Hospital, Brazil); Nacif L* (Hospital Nove De Julho, Brazil); Flumignan RLG*, Nakano LCU, Kuramoto DAB, Aidar ALS, Pereda MR, Correia RM, Santos BC, Carvalho AA, Amorim JE, Guedes Neto HJ, Areias LL, Sousa AF, Flumignan CDQ, Lustre WG, Moreno DH, Barros-Jr N, Baptista-Silva JCC (Hospital São Paulo, Brazil); Matos LL*, Kowaski LP, Kulcsar MAV, Nunes KS, Teixeira MF (Instituto do Câncer do Estado de São Paulo, São Paulo, Brazil); Nunes RL*, Ijichi TR, Kim NJ, Marreiro A, Muller B, Barakat Awada J (Notre Dame Intermédica - Hospital Salvalus, Brazil); Baiocchi G*, Kowalski LP*, Vartanian JG, Makdissi FB, Aguiar Jr S*, Marques N, Carvalho GB, Marques TMDM, Abdallah EA, Zurstrassen CE*, Gross JL*, Zequi SC*, Gonçalves BT, Santos SS, Duprat JP, Coimbra FJF* (A.C. Camargo Cancer Center, Brazil); Cicco R* (Instituto de Câncer Dr Arnaldo Vieira de Carvalho, São Paulo, Brazil); Takeda F, Cecconello I, Ribeiro Junior U (Instituto Do Câncer De Estado De São Paulo, Brazil); Gatti A*, Oliva R, Nardi C (Hospital Geral De Pirajussara, Brazil); Slavchev M*, Atanasov B, Belev N (University Hospital Eurohospital, Plovdiv, Bulgaria); Dell A, Bigam D*, Dajani K, Al Riyami S (University of Alberta Hospital, Canada); Martin J*, Cheng D, Yang H, Fayad A (London Health Sciences Centre and St Josephs Health Care London, Canada); Carrier FM*, Amzallag E, Desroches J, Ruel M (Centre hospitalier de l'Université de Montréal, Canada); Caminsky NG, Boutros M*, Moon J, Wong EG, Vanounou T, Pelletier J, Wong S, Girsowicz E, Bayne J, Obrand D, Gill H, Steinmetz O, MacKenzie K, Lukaszewski M, Jamjoum G (Jewish General Hospital, Canada); Richebé P, Verdonck O, Discepola S, Godin N, Idrissi M (Hôpital Maisonneuve-Rosemont, Canada); Briatico D, Sharma S, Talwar G, Bailey K (McMaster Children's Hospital, Hamilton, Ontario, Canada); Lecluyse V* (Hôpital du Sacré-Cœur-de-Montréal, Canada); Côté G (Centre hospitalier universitaire Sainte-Justine, Canada); Demyttenaere S*, Garfinkle R, Boutros M* (St. Mary's Hospital, Canada); Kouyoumdjian A, Boutros M*, Dumitra S, Moon J, Wong EG, Khwaja K, Luo L, Lukaszewski M, Gill H, Berry G, Liberman AS, Girsowicz E, Bayne J, Obrand D, Steinmetz O, MacKenzie K, Schmid S, Spicer J, Al Farsi M* (McGill University Health Center, Canada); Abou-Khalil J (The Ottawa Hospital, Canada); Couture E*, Mohammadi S, Tremblay H, Gagné N, Bergeron A (Institut universitaire de cardiologie et de pneumologie de Québec , Canada); Turgeon Af*, Costerousse O, Bellemare D, Babin C, Blier C (Centre hospitalier universitaire de Québec, Canada); Wood ML, Persad A, Groot G*, Pham H (Saskatoon City Hospital/Royal University Hospital/St. Paul's Hospital, Canada); D'Aragon F*, Carbonneau E, Bouchard M, Masse M, Pesant F, Héroux J (Centre hospitalier universitaire de Sherbrooke, Canada); Karanicolas P*, Hallet J, Nadler A, Nathens A (Sunnybrook Hospital, Canada); Ko M*, Brar A (St. Joseph's Health Centre, Canada); Mayson K* (Vancouver General Hospital, Canada); Kidane B*, Srinathan S (Health Sciences Centre, Canada); Escudero MI*, Reyes JT (Hospital Clinico Universidad De Chile/ Hospital San Jose, Chile); Modolo MM*, Ramirez Nieto P, Sepulveda R, Bolbarán A, Molero A, Ruiz I (Barros Luco Trudeau, Chile); Reyes GP*, Salas R, Suazo C (Hospital del Salvador, Chile); Muñoz R*, Grasset E*, Inzunza M, Besser N, Irarrázaval MJ, Jarry C, Bellolio F, Romero Manqui

For peer review only - http://bmjopen.bmj.com/site/about/guidelines.xhtml

CA, Ruiz Esquide M (Hospital Clínico Universidad Católica, Chile); Fuentes T*, Campos J (Hospital Sotero Del Rio, Chile); Perez Rivera CJ*, Cabrera PA (Fundacion Cardioinfantil-Instituto de Cardiología, Colombia); Pinilla RE*, Guevara O, Jimenez Ramirez LJ, Velasquez Cuasquen BG, Herrera Mora DR, Bonilla A, Diaz S*, Manrique E, Facundo H, Velez Bernal JL, Ángel J, García M, Guzmán L, Lehmann C, Cervera S, Trujillo Sanchez LM (Instituto Nacional De Cancerologia, Colombia); Guevara R*, Valbuena D, Suarez L, Jimenez G, Velandia A, Vargas J, Espinosa J, Rey S (Clínica Universitaria Colombia, Bogotá., Colombia); Mendoza Quevedo Jairo (Subred Sur Occidente de Kennedy (Hospital de Kennedy), Bogota, Colombia); Calvache JA*, Orozco-Chamorro CM, Sánchez-Gómez TA, Rojas-Tejada DA (Hospital Universitario San José, Popayán, Colombia); Mihanovic J*, Bakmaz B, Rakvin I, Sulen N, Andabaka T (Zadar General Hospital, Croatia); Luksic I*, Mamic M (University Hospital Dubrava, Zagreb, Croatia); Martinek L, Skrovina M* (Hospital & Oncological Centre Novy Jicin, Czechia); Žatecký J*, Peteja M (Slezská nemocnice v Opavě, p.o., Czechia); Kristensen HØ, Mekhael M, Christensen P*, Westh L (Aarhus University Hospital, Denmark); Smith H*, Haugstvedt AF, Jönsson ML (Bispebjerg Hospital, Denmark); Crespo A, Batista S*, Rodriguez-Abreu J*, Tactuk N, Diaz-Delgado PJ, Rivas R (Cedimat - Centro De Diagnóstico, Medicina Avanzada, Laboratorio Y Telemedicina, Dominican Republic); Sarmiento-Bobadilla JA (Hospital General Norte de Guayaquil, Guayaquil, Ecuador); Ashoush F, Samir Abdelaal A, Qatora MS, Elsayed Hewalla ME, Metwalli M, Atta R, Abdelmajeed A, Abosamak NE, Sabry A*, Shehata S* (Alexandria Main University Hospital, Egypt); Sallam I*, Amira G, Sherief M, Sherif A (Misr Cancer Center, Egypt); Salem H*, Hamdy R, Aboulkassem H, Ghaly G*, Sherif G, Morsi A, Abdelrahman A (National Cancer Institute, Egypt); Omnia Ahmed (Coptic Hospital, Cairo, Egypt); Tawheed A, El Kassas M*, Omar W (Endemic Medicine Department, Faculty of Medicine, Helwan University, Egypt); Abdelsamed A, Seleim A, Salem H*, Azzam AY* (Al Azhar University Hospitals, Egypt); ElFiky M*, Nabil A (Kasr Alainy Faculty of Medicine, Cairo University, Egypt); Ibraheem M*, Ghaly G, ELDeeb M, Fawzy M (Baheya Center for Early detection and Treatment of Breast Cancer , Egypt); Hamed H* (Elkheir Hospital, Egypt); Emile S*, Elfallal A, Elfeki H, Shalaby M, Sakr A (Mansoura University Hospital, Egypt); Alrahawy M*, Atif H, Sakr A, Soltan H (Menofiya University Hospital, Egypt); Sayed AK, Salah A, Atiya A* (Minia University Hospital, Egypt); Wassim K* (Quwaysna Central Hospital, Egypt); Abbas AM, Abd Elazeem HAS, Abd-Elkariem AY, Abd-Elkarem MM, Alaa S, Ali AK, Ashraf M, Ayman A, Azizeldine MG, Elkhayat H, Emad Mashhour A, Gaber M, Hamza HM, Hawal I, Hetta HF, Elghazaly SM, Mohammed MM, Monib FA, Nageh MA, Saad A, Saad MM, Shahine M, Yousof EA, Youssef A (Assiut University Hospital, Assiut, Egypt); Esmail E*, Khalaf M (Kafr Elzyat Hospital, Egypt); Eldaly A* (El-Menshawy Hospital, Egypt); Ghoneim A*, Hawila A, Badr H, Elhalaby I, Abdel-bari M, Elbahnasawy M, Hamada MK, Morsy MS, Hammad M, Hammad M, Essa M, Fayed MT, Elzoghby M, Rady M, Hamad O, Salman S, Sarsik S, Abd-elsalam S, Gamal Badr S, El-Masry Y (Tanta University Hospital, Egypt); Moahmmed MMH (Zagazig University Hospitals, Zagazig, Egypt); Hailu S*, Wolde A, Mengesha M, Nida S, Workneh M, Y Ahmed M, Fisseha T, Kassa D, Zeleke H, Admasu A, Laeke T, Tirsit A, Gessesse M, Addissie A (Tikur Anbessa (Black Lion) Hospital-Addis Ababa University, Ethiopia); Bekele K* (Maddawalabu University Goba Referral Hospital, Ethiopia); Kauppila JH*, Sarjanoja E (Länsi-Pohja Central Hospital, Finland); Testelin S*, Dakpé S, Devauchelle B, Bettoni J, Lavagen N (CHU Amiens, France); Schmitt F*, Lemée JM, Boucher S, Breheret R, Kün-Darbois JD, Kahn A, Gueutier A, Bigot P (CHU Angers, France); Borraccino B (Centre Hospitalier Auxerre, Auxerre, France); Lakkis Z*, Doussot A, Heyd B, Manfredelli S, Mathieu P, Paquette B, Turco C, Barrabe A, Louvrier A (CHU Besançon, France); Moszkowicz D*, Giovinazzo D, Bretagnol F (Hôpital Louis-Mourier - Ap-Hp, France); Police A*, Charre L, Volpin E, Braham H, El Arbi N, Villefranque V, Bendjemar L (Hôpital Simone Veil, France); Girard E*, Abba J, Trilling B (CHU Grenoble-Alpes, Hôpital Michallon, France); Chebaro A*, Lecolle K, Truant S, El Amrani M, Zerbib P, Pruvot FR, Mathieu D, Surmei E, Mattei L, Marin H (CHU Lille, France); Christou N, Ballouhey Q*, Ferrero P, Coste Mazeau P, Tricard J, Barrat B, Taibi A, Usseglio J, Laloze J, Salle H, Fourcade L (Chu Limoges, France); Duchalais E*, Regenet N, Rigaud J, Waast D, Denis W, Malard O, Buffenoir K, Espitalier F, Ferron C, Varenne Y, Crenn V, De Vergie S, Cristini J, Samarut E (Nantes University Hospital, France); Tzedakis S*, Bouche PA*, Gaujoux S (Hôpital Cochin - Aphp, France); Kantor E (AP-HP Hopital Bichat Claude Bernard, Paris, France); Gossot D, Seguin-Givelet A*, Fuks D, Grigoroiu M, Sanchez Salas R, Cathelineau X, Macek P, Barbé Y, Rozet F, Barret E, Mombet A, Cathala N, Brian E, Zadegan F, Conso C (Institut Mutualiste Montsouris, France); Blanc T*, Broch A, Sarnacki S, (Necker Enfants Malades University Children Hospital - APHP, Paris ; Université de Paris, Paris, France); Ali L, Bonnard A, Peycelon M* (Robert-Debré Children University Hospital - APHP, Paris ; Université de Paris, Paris, France); Hervieux E*, Clermidi P, Maisonneuve E, Aubry E, Thomin A, Langlais T (Hôpital Trousseau - Aphp, France); Passot G*,Glehen O, Cotte E, Lifante JC (Hopital Lyon Sud, Pierre Bénite, France); De Simone B*, Chouillard E* (Centre Hospitalier Intercommunal Poissy Saint Germain En Laye, France); Arnaud AP*, Violas P (CHU Rennes - Hôpital Sud, France); Bergeat D, Merdrignac A, Arnaud AP (Chu Rennes - Hopital Pontchaillou, France);

Scalabre A*, Perotto LO, Le Roy B, Haddad E, Vermersch S (Chu Saint Etienne, France); Ezanno AC*, Barbier O, Vigouroux F, Malgras B, Aime A (Hia Begin, France); Seeliger B*, Mutter D, Philouze G, Pessaux P (IHU-Strasbourg / Strasbourg University Hospitals, France); Germain A*, Chanty H, Ayav A (CHRU Nancy, France); Kassir R*, Von theobald P, Sauvat F (CHU Reunion, France); O'Connor J*, Mayombo Idiata M, O'Connor Z, Tchoba S (Bongolo Hospital, Gabon); Modabber A*, Winnand P, Hölzle F (University Hospital Aachen, Germany); Sommer B, Shiban E, Wolf S, Anthuber M*, Sommer F (University Hospital Augsburg, Germany); Kaemmerer D*, Schreiber T (Zentralklinik Bad Berka, Germany); Kamphues C*, Lauscher JC, Schineis C, Loch FN, Beyer K (Charité University Medicine, Campus Benjamin Franklin, Germany); Nasser S, Sehouli J* (Charité Comprehensive Cancer Center, Germany); Höhn P, Braumann C*, Reinkemeier F, Uhl W (St Josef-Hospital, Bochum, Germany); Weitz J, Bork U*, Welsch T, Praetorius C, Korn S, Distler M (University Hospital Carl Gustav Carus, Technical University Dresden, Germany); Fluegen G, Knoefel WT, Vay C* (University Hospital Duesseldorf, Germany); Golcher H, Grützmann R*, Binder J (Universitätsklinikum Erlangen, Germany); Meister P*, Gallinat A, Paul A (University Hospital Essen, Essen, Germany); Schnitzbauer AA*, Thoenissen P, El Youzouri H, Schreckenbach T, Nguyen TA (Frankfurt University Hospital, Goethe University, Frankfurt, Germany); Eberbach H*, Bayer J*, Erdle B, Sandkamp R (Medical Center – Albert-Ludwigs-University of Freiburg, Faculty of Medicine, Germany); Nitschke C, Izbicki J, Uzunoglu FG*, Koenig D, Gosau M, Böttcher A, Heuer A, Klatte TO, Priemel M, Betz CS, Burg S, Möckelmann N, Busch CJ, Bewarder J, Zeller N, Smeets R, Thole S, Vollkommer T, Speth U, Stangenberg M (University Medical Center Hamburg-Eppendorf, Germany); Hakami I*, Boeker C, Mall J* (KRH Nordstadt-Siloah Hospitals, Hannover, Germany); Schardey HM , von Ahnen T*, von Ahnen M, Brunner U (Agatharied Hospital, Hausham, Germany); Tapking C*, Kneser U, Hirche C, Jung M, Kowalewski KF (BG Trauma Center Ludwigshafen, Germany); Kienle P* (Theresienkrankenhaus, Mannheim, Germany); Reissfelder C, Seyfried S*, Herrle F, Hardt J, Galata C, Birgin E, Rahbari N, Vassos N (Mannheim University Medical Center (Universitätsmedizin Mannheim), Germany); Stoleriu MG*, Hatz R (Asklepios Pulmonary Hospital, Munich Gauting, Germany); Albertsmeier M*, Börner N, Lampert C, Werner J (Department of General, Visceral and Transplantation Surgery, Ludwig-Maximilians-Universität Munich, Munich, Germany); Kuehlmann B, Prantl L (University Hospital Regensburg, Regensburg, Germany); Brunner SM*, Schlitt HJ, Brennfleck F, Pfister K*, Oikonomou K (University Medical Center Regensburg, Department of Surgery, Germany); Reinhard T, Nowak K* (RoMed Klinikum Rosenheim, Germany); Ronellenfitsch U*, Kleeff J, Delank KS, Michalski CW, Szabo G (University Hospital Halle (Saale), Germany); Widyaningsih R, Stavrou GA (Klinikum Saarbrücken, Germany); Bschorer R*, Mielke J, Peschel T (Helios Kliniken Schwerin, Germany); Königsrainer A*, Quante M, Löffler MW, Yurttas C (University Hospital Tübingen, Department of General, Visceral and Transplant Surgery, Germany); Doerner J*, Seiberth R (Helios Universitätsklinikum Wuppertal (Universität Witten/Herdecke), Germany); Bouchagier K*, Klimopoulos S, Paspaliari D, Stylianidis G (Evaggelismos General Hospital, Greece); Syllaios A, Baili E, Schizas D, Liakakos T, Charalabopoulos A*, Zografos C, Spartalis E (Laiko University Hospital, Greece); Manatakis DK*, Tasis N, Antonopoulou MI (Athens Naval and Veterans Hospital, Greece); Xenaki S*, Xynos E*, Chrysos E, Athanasakis E, Tsiaousis J (University Hospital of Heraklion Crete and Interclinic Hospital of Crete, Greece); Lostoridis E*, Tourountzi P (Kavala General Hospital, Greece); Tzovaras G*, Tepetes K, Zacharoulis D, Baloyiannis I, Perivoliotis K, Hajiioannou J*, Korais C, Gkrinia E, Skoulakis CE, Saratziotis A, Koukoura O, Symeonidis D, Diamantis A (General University Hospital of Larissa, Greece); Tsoulfas G, Christou CD, Tooulias A, Papadopoulos V*, Anthoulakis C, Grimbizis G, Zouzoulas D, Tsolakidis D (Papageorgiou General Hospital, Greece); Tatsis D, Christidis P, Loutzidou L, Ioannidis O*, Astreidis I, Antoniou A, Antoniadis K*, Vachtsevanos K, Paraskevopoulos K, Kalaitsidou I, Alexoudi V, Stavroglou A, Mantevas A, Michailidou D, Grivas T, Deligiannidis D, Politis S, (George Papanikolaou General Hospital of Thessaloniki, Greece); Barrios Duarte A*, Portilla AL, Lowey MJ, Recinos G, Lopez Muralles I (Hospital General De Enfermedades, Guatemala); Siguantay MA* (Hospital Roosevelt, Guatemala City, Guatemala); Estrada EE*, Aguilera-Arévalo ML*, Cojulun JM, Echeverría-Dávila G (Hospital General San Juan De Dios, Guatemala City, Guatemala); Marín C*, Icaza de Marín GC* (Hospital Regional De Zacapa, Guatemala); Kok SY, Joeng HKM, Chan LL, Lim D (United Christian Hospital, Hong Kong); Novak Z*, Echim T (National Institute of Oncology, Hungary); Suszták N, Banky B* (Szent Borbála Kórház, Hungary); Kembuan G, Pajan H, Islam AA* (Rsud Wahidin Sudirohusodo, Indonesia); Rahim F (Baghaei Hospital, Iran); Safari H (Golestan Hospital & Ahvaz Jundishapur University of Medical Sciences, Iran); Mozafari M, Brouki Milan P*, Tizmaghz A, Rezaei Tavirani M (Firoozabadi Hospital, Iran); Ahmed A (Baghdad Medical City, Iraq); Hussein R* (Zafaraniyah General Hospital, Iraq); Fleming C, O'Brien S, Kayyal MY, Daly A, Killeen S, Corrigan M* (Cork University Hospital, Ireland); De Marchi J, Hill A*, Farrell T, Davis NF, Kearney D, Nelson T (Beaumont Hospital, Ireland); Maguire PJ*, Barry C*, Farrell R, Smith LA, Mohan HM, Mehigan BJ, Mccormick P, Larkin JO*, Fahey BA, Rogers A, Donlon N, O'Sullivan H, Nugent T, Reynolds JV*, Donohue C, Shokuhi P, Ravi N,

Fitzgerald C, Lennon P, Timon C, Kinsella J, Smith J, Boyle T, Alazawi D, Connolly E, Butt W, Croghan SM, Manecksha RP (St. James's Hospital, Ireland); Fearon N*, Winter D*, Heneghan H*, Maguire D, Gallagher T, Conlon K, Kennedy N, Martin S, Kennelly R, Hanly A, Ng KC, Fagan J, Geary E, Cullinane C, Hanly A, Carrington E, Geraghty J, McDermott E, Pritchard R, McPartland D, Boland M, Stafford A, Maguire D, Geoghegan J (St Vincent's University Hospital, Ireland); Elliott JA*, Ridgway PF, Gillis AE, Bass GA, Neary PC, O'Riordan JM, Kavanagh DO, Reynolds IS, Conlon K, Joyce DP, Boyle E, Egan B, Whelan M, Elkady R, Tierney S, Connelly TM, Earley H, Umair M, O'Connell C, Manecksha RP, Thomas AZ, Rice D, Madden A, Bashir Y, Creavin B (Tallaght Hospital, Ireland); Cullivan O, Owens P, Canas-Martinez A, Murphy C, Pickett L, Murphy B, Mastrosimone A, Beddy D, Arumugasamy M, Allen M, Aremu M* (Connolly Hospital Blanchardstown, Ireland); McCarthy C, O'Connor C, O'Connor DB*, Kent E, Malone F, Geary M (Rotunda Hospital, Ireland); McKevitt KL*, Lowery AJ*, Ryan ÉJ, Aherne TM, Fowler A, Hassanin A, Hogan AM, Collins CG, Finnegan L, Carroll PA, Kerin MJ, Walsh SR (University Hospital Galway, Ireland); Nally D*, Peirce C, Coffey JC, Cunningham RM, Tormey S (University Hospital Limerick, Ireland); Hardy NP*, Neary PM (University Hospital Waterford/University College Cork, Ireland); Muallem-Kalmovich L, Kugler N, Lavy R, Zmora O* (Shamir Medical Center, Israel); Horesh N* (Sheba Medical Center, Israel); Vergari R* (Ospedali Riuniti Di Ancona, Italy); Mochet S*, Barmasse R, Usai A, Morelli L (Ospedale Regionale Umberto Parini, Italy); Picciariello A*, Papagni V, Altomare DF (Azienda Ospedaliero Universitaria Consorziale Policlinico Di Bari, Italy); Colledan M*, Zambelli MF, Tornese S, Camillo A, Rausa E*, Bianco F, Lucianetti A (Asst-Papa Giovanni Xxiii- Bergamo, Italy); Prucher GM*, Baietti AM, Ruggiero F, Maremonti P, Neri F, Ricci S, Biasini M, Zarabini AG (Ospedale Maggiore/Bellaria Carlo Alberto Pizzardi Ausl Bologna, Italy); Belvedere A, Bernante P, Bertoglio P, Boussedra S, Brunocilla E, Cipriani R, Cisternino G, De Crescenzo E, De Iaco P, Della Gatta AN, Dondi G, Frio F, Jovine E, Mineo Bianchi F, Neri J, Parlanti D, Perrone AM, Pezzuto AP, Pignatti M, Pilu G, Pinto V, Poggioli G, Ravaioli M, Rottoli M*, Schiavina R, Serenari M, Serra M, Solli P*, Taffurelli M, Tanzanu M, Tesei M, Violante T, Zanotti S (Sant'orsola Hospital, Alma Mater Studiorum University of Bologna, Italy, Italy); Tonini V*, Sartarelli L, Cervellera M, Gori A (S.Orsola-Malpighi Hospital, Italy); Armatura G*, Scotton G, Patauner S, Frena A (St. Moritz Hospital, Italy); Podda M*, Pisanu A, Esposito G, Frongia F (Cagliari University Hospital, Italy); Abate E, Laface L, Casati M*, Schiavo M, Casiraghi T (Ospedale Vittorio Emanuele III - Carate Brianza, Italy); Sammarco G, Gallo G*, Vescio G, Fulginiti S, Scorcia V, Giannaccare G, Carnevali A (University 'Magna Graecia' of Catanzaro, Italy); Giuffrida MC*, Marano A, Palagi S, Di Maria Grimaldi S, Testa V, Peluso C, Borghi F, Simonato A, Puppo A, D'Agruma M, Chiarpenello R, Pellegrino L, Maione F, Cianflocca D, Pruiti Ciarello V, Giraudo G, Gelarda E, Dalmasso E, Abrate A, Daniele A, Ciriello V, Rosato F, Garnero A, Leotta L (Santa Croce E Carle Hospital, Cuneo, Italy); Giacometti M*, Zonta S (San Biagio Hospital, Domodossola - ASL VCO, Italy); Lomiento D, Taglietti L, Dester S, Compagnoni B, Viotti F, Cazzaniga R, Del Giudice R (Asst Valcamonica Ospedale Di Esine, Italy); Mazzotti F*, Pasini F, Ugolini G (Ospedale per gli Infermi di Faenza, Italy); Fabbri N, Feo CV*, Righini E, Gennari S (Azienda Unità Sanitaria Locale di Ferrara, Università di Ferrara, Italy); Chiozza M, Anania G*, Urbani A, Koleva Radica M, Carcoforo P*, Portinari M, Sibilla M (Azienda Ospedaliero Universitaria Sant'Anna, Italy); Anastasi A, Bartalucci B, Bellacci A, Canonico G*, Capezzuoli L, Di Martino C, Ipponi P, Linari C, Montelatici M, Nelli T, Spagni G, Tirloni L, Vitali A (Ospedale San Giovanni Di Dio, Italy); Agostini C, Alemanno G, Bartolini I, Bergamini C, Bruscino A, Checcucci C, De Vincenti R, Di Bella A, Fambrini M, Fortuna L, Maltinti G, Muiesan P, Petraglia F, Prosperi P*, Ringressi MN, Risaliti M, Sorbi F*, Taddei A* (Azienda Ospedaliera Universitaria Careggi, Italy); Lizzi V*, Vovola F, Arminio A, Cotoia A, Sarni AL (Ospedali Riuniti Azienda Ospedaliera Universitaria Foggia, Italy); Familiari P*, D'Andrea G, Picotti V, Bàmbina F (Fabrizio Spaziani, Italy); Fontana T* (Vittorio Emanuele, Italy); Barra F*, Ferrero S, Gustavino C, Kratochwila C, Ferraiolo A, Costantini S, Batistotti P, Aprile A, Almondo C, Ball L, Robba C, Scabini S, Pertile D, Massobrio A, Soriero D (IRCCS Ospedale Policlinico San Martino, Italy); D'Ugo S*, Depalma N, Spampinato MG ("Vito Fazzi" Hospital, Italy); Lippa L, Gambacciani C, Santonocito OS*, Aquila F, Pieri F (Spedali Riuniti Di Livorno, Italy); Ballabio M*, Bisagni P, Longhi M, Armao T, Madonini M, Gagliano A, Pizzini P (Ospedale Maggiore Di Lodi, Italy); Costanzi A*, Confalonieri M, Monteleone M, Colletti G, Frattaruolo C, Mari G (San Leopoldo Mandic, Italy); Spinelli A*, Mercante G, Spriano G, Gaino F, Ferreli F, De Virgilio A, Rossi V, Carvello MM, Di Candido F, Kurihara H, Marrano E, Torzilli G, Castoro C, Carrano FM (Humanitas Clinical and Research Center - IRCCS, Rozzano (MI), Italy); Martinelli F, Macchi A, Fiore M*, Pasquali S, Cioffi SPB, Baia M, Abatini C, Sarre C, Mosca A, Biasoni D, Gronchi A, Citterio D, Mazzaferro V, Cadenelli P, Gennaro M, Capizzi V, Guaglio M, Sorrentino L, Bogani G, Sarpietro G, Giannini L, Comini LV, Rolli L, Folli S, Raspagliesi F, Piazza C, Cosimelli M, Salvioni R (Fondazione IRCCS Istituto Nazionale dei Tumori, Milano, Italy); Antonelli B, Baldari L, Boni L, Cassinotti E*, Pignataro L, Rossi G, Torretta S, Beltramini GA, Gianni' A (Fondazione IRCSS Ca' Granda - Ospedale Maggiore Policlinico, Italy); Tagliabue M, De Berardinis R, Pietrobon G, Chu F, Cenciarelli S, Adamoli L, Ansarin M*,

For peer review only

Fumagalli Romario U*, Mastrilli F (Istituto Europeo Di Oncologia - IRCCS, Milano, Italy); Mariani NM*, Nicastro V (Asst Santi Paolo E Carlo, Italy); Cellerino P* (Ospedale Fatebenefratelli E Oftalmico, Italy); Colombo F*, Frontali A, Bondurri A, Guerci C, Maffioli A, Ferrario L (Ospedale Luigi Sacco Milano, Italy); Candiani M*, Bonavina G, Ottolina J, Valsecchi L, Mortini P, Gagliardi F, Piloni M, Medone M, Negri G, Bandiera A, De Nardi P, Sileri P, Carlucci M, Pelaggi D, Rosati R, Vignali A, Parise P, Elmore U (San Raffaele Scientific Institute, Milan, Italy); Tamini N*, Nespoli LC, Rennis M, Pitoni L, Chiappetta MF, Vico E, Fruscio R, Grassi T (Ospedale San Gerardo, Università degli Studi di Milano Bicocca, Italy); Sasia D*, Migliore M, Gattolin A, Rimonda R, Travaglio E, Olearo E (Regina Montis Regalis Hospital, Mondovì, Italy); Tufo A*, Marra E, Maida P, Marte G, Tammaro P (Ospedale Del Mare, Italy); Bianco F*, Incollingo P (Ospedale S. Leonardo - Asl Napoli 3 Sud, Castellammare Di Stabia, Italy); Izzo F, Belli A, Patrone R, Albino V, Leongito M, Granata V, Piccirillo M, Palaia R (Istituto Nazionale Tumori Fondazione, G. Pascale-IRCCS, Naples, Italy); Francone E*, Gentilli S, Nikaj H (Azienda Ospedaliero Universitaria Maggiore Della Carità, Novara, Italy); Fiorini A, Norcini C, Chessa A* (San Giovanni Di Dio, Italy); Marino MV*, Mirabella A, Vaccarella G (Azienda Ospedaliera, Ospedali Riuniti Villa Sofia-Cervello, Palermo, Italy); Musini L, Ampollini L*, Bergonzani M, Varazzani A*, Bellanti L, Domenichini M, Rossi G, Cabrini E, Fornasari A, Freyrie A*, Dejana DO, D'Angelo G*, Bertoli G, Di Lella F, Bocchialini G, Falcioni M, Lanfranco D, Poli T (Azienda Ospedaliero-Universitaria di Parma, Italy); Giuffrida M, Annicchiarico A, Perrone G, Catena F* (Parma University Hospital, Italy); Raffaele A* (Policlinico San Matteo, Italy); De Manzoni Garberini A* (Ospedale Civile Spirito Santo, Italy); Baldini E*, Conti L, Ribolla M, Capelli P, Isolani SM, Maniscalco P, Cauteruccio M, Ciatti C, Puma Pagliarello C, Gattoni S (Ospedale "Guglielmo Da Saliceto" - Dipartimento di Chirurgia - Piacenza, Italy); Galleano R*, Malerba M, Ciciliot M (Ospedale Santa Corona, Pietra Ligure (SV), Pietra Ligure, Italy); Farnesi F, Calabrò M*, Pipitone Federico NS, Lunghi EG, Muratore A (Edoardo Agnelli, Italy); Morelli L*, Di Franco G, Palmeri M, Tartaglia D*, Coccolini F, Chiarugi M, Simoncini T*, Gadducci A, Caretto M, Giannini A, Perutelli A, Domenici L*, Garibaldi S, Capanna R, Andreani L, Furbetta N, Guadagni S, Bianchini M, Gianardi D (Azienda Ospedaliero Universitaria Pisana, Italy); Pinotti E*, Montuori M, Carissimi F, Baronio G (Policlinico San Pietro, Italy); Zizzo M*, Castro Ruiz C, Annessi V, Montella MT, Falco G*, Mele S, Ferrari G, Mastrofilippo V, Mandato VD*, Aguzzoli L (Azienda Unità Sanitaria Locale - IRCCS Di Reggio Emilia, Italy); Corbellini C, Baldi C*, Sampietro GM (Ospedale Di Rho - Asst Rhodense, Italy); Palini GM, Zanini N*, Garulli G (Ospedale Infermi di Rimini, Rimini, Italy); Barone R*, Murgese A, Mungo S, Grasso M, Marafante C, Birolo SL, Moggia E, Caccetta M, Masciandaro A, Deirino A, Garino M (Ospedale Degli Infermi Di Rivoli, Italy); Perinotti R, Maiello F (Ospedale degli Infermi, Biella, Italy); Gordini L, Lombardi CP, Marzi F, Marra AA, Ratto C, Di Muro M, Litta F, De Simone V, Cozza V*, Rosa F, Agnes A, Parello A, Alfieri S, Sganga G (Fondazione Policlinico Universitario Agostino Gemelli IRCCS, Italy); Lapolla P*, Mingoli A, De Toma G, Fiori E, La Torre F, Sapienza P, Brachini G, Cirillo B, Iannone I, Zambon M, Chiappini A, Meneghini S, Fonsi GB, Cicerchia PM, Bruzzaniti P, Santoro A, Frati A, Marruzzo G, Ribuffo D (Policlinico Umberto I Sapienza University of Rome, Italy); Sagnotta A*, Marino Cosentino L, Mancini S (Ospedale San Filippo Neri, Italy); Lisi G*, Spoletini D (Sant'Eugenio Hospital, Italy); Bellato V Campanelli M, Sica G*, Siragusa L, (Policlinico Tor Vergata Hospital, Rome, Italy); Bonavina L*, Asti E, Bernardi D, Lovece A (IRCSS Policlinico San Donato, University of Milan, Italy); Perra T, Porcu A*, Fancellu A, Feo CF, Scanu AM (Cliniche San Pietro, A.O.U. Sassari, Italy); Tuminello F, Galleano R*, Franceschi A, Langone A (San Paolo, Italy); Fleres F*, Spolini A, Bordoni P, Franzini M, Clarizia G, Grechi A, Longhini A (Ospedale Di Sondrio (Asst Valtellina E Alto Lario), Italy); Guaitoli E, Manca G (Perrino Hospital Brindisi, Italy); Grossi U, Novello S, Zanus G*, Romano M, Rossi S (Ca' Foncello Treviso - DISCOG, Università di Padova, Italy); Ferrara F (San Carlo Borromeo Hospital, ASST Santi Paolo e Carlo, Italy); La Torre M (Fabia Mater Hospital, Rome, Italy); Pirozzolo G*, Recordare A (Dell'Angelo Hospital, ULSS3 Serenissima, Venezia, Italy); Paiella S, Turri G, Rattizzato S, Campagnaro T*, Guglielmi A, Pedrazzani C, Ruzzenente A, Poletto E, Conci S, Casetti L, Fontana M, Salvia R*, Malleo G, Esposito A, Landoni L, De Pastena M, Bassi C, Tuveri M, Nobile S , Marchegiani G, Bortolasi L* (Azienda Ospedaliera Universitaria Integrata Di Verona, Italy); Sambugaro E*, Malavolta M*, Moretto G, Impellizzeri H, Inama M* (Ospedale Pederzoli, Italy); Barugola G, Ascari F, Ruffo G (IRCCS Ospedale Sacro Cuore Don Calabria, Negrar di Valpolicella (Verona), Italy); Granieri S*, Cotsoglou C (Asst Vimercate, Italy); Berselli M*, Desio M, Marchionini V, Cocozza E (ASST Settelaghi, Varese, Italy); Di Saverio S*, Ietto G, Iovino D, Carcano G (University of Insubria, Ospedale di Circolo e Fondazione Macchi (Varese), Varese Lombardy, Italy); Ayasra F*, Qasem A, Ayasra Y (Al-Basheer Hospital, Jordan); Al-Masri M*, Abou Chaar MK, Al-Najjar H, Ghandour K, Alawneh F, Abdel Jalil R, Abdel Al S, Elayyan M, Ghanem R, Lataifeh I, Alsaraireh O (King Hussein Cancer Center, Jordan); Abu Za'nouneh FJ, Fahmawee T, Ibrahim A, Obeidat K* (King Abdullah University Hospital, Jordan); Lee KJ (Keimyung University School of Medicine, Daegu, Korea); Shin SJ, Chung H (Keimyung University, Daegu, Korea); Albader I, Alabbad J, Albader MAS (Mubarak Al-Kabeer Hospital, Jabriya, Kuwait); Bouhuwaish A*,

Taher AS, Omar MSM (Tobruk Medical Center, Libya); Abdulwahed E*, Biala M, Morgom M (Tripoli Central Hospital, Libya); Elhadi A, Alarabi A, Msherghi A*, Elhajdawe F, Alsoufi A (Tripoli University Hospital, Libya); Salamah A*, Salama H, Bulugma M*, Almabrouk H (Zawia Teaching Hospital, Libya); Venskutonis D*, Dainius E, Kubiliute E, Bradulskis S, Parseliunas A, Kutkevicius J, Subocius A (Lithuanian University of Health Sciences Kaunas Clinical Hospital, Lithuania); Cheong YJ*, Masood MS* (Hospital Raja Permaisuri Bainun, Ipoh, Malaysia); Ngo CW*, Saravanan R, Abdul Maei N (Hospital Enche' Besar Hajjah Khalsom, Malaysia); Hayati F, Amin Sahid N (Queen Elizabeth Hospital & Universiti Malaysia Sabah, Kota Kinabalu, Sabah, Malaysia); Yanowsky Reyes G, Orozco Perez J, Damian R, Santana Ortiz R, Colunga Tinajero CA (Antiguo Hospital Civil De Guadalajara, Mexico); Cordera F*, Gómez-Pedraza A, Maffuz-Aziz A, Posada JA, De la Rosa Abaroa MA, Alvarez MR, Arrangoiz R, Hernández R (ABC Medical Center, Mexico); Bozada Gutierrez K, Trejo-Avila M*, Valenzuela-Salazar C, Herrera-Esquivel J, Moreno-Portillo M (Hospital General Dr. Manuel Gea González, Mexico); Pinto-Angulo VM*, Sosa-Duran EE, Ziad-Aboharp H, Jimenez Villanueva X (Hospital Juárez de México, Ciudad de México, Mexico); Soulé Martínez CE*, Lupián-Angulo AI (Hospital Central Norte Pemex, Mexico); Martínez Zarate J Jacobo*, Reyes Rodriguez E, Montalvo Dominguez G (Hospital Médica Sur, Ciudad de México, Mexico); Becerra García FC* (Hospital San Ángel Inn Chapultepec, Mexico); Melchor-Ruan J, Vilar-Compte D*, Romero-Bañuelos E, Herrera-Gomez A, Meneses-Garcia A, Isla-Ortiz D, Salcedo-Hernández RA, Hernández-Nava JM, Morales-Castelan JE (Instituto Nacional De Cancerologia, Mexico); Sarre C, Posadas-Trujillo OE, Buerba GA, Alfaro-Goldaracena A, Pena Gomez-Portugal E, Lopez-Pena G; Hinojosa CA*, Mercado MA* (Universidad Nacional Autonoma de Mexico, Instituto Nacional de Ciencias Medicas y Nutricion Salvador Zubiran, Mexico City, Mexico); Ramos-De la Medina A*, Martinez L, Duran I, Gonzalez DS, Martinez MJ, Nayen Sainz de la Fuente A (Hospital Español Veracruz, Mexico); Miguelena L, Hernández Miguelena L (Hospital Regional Veracruz, Mexico); Louraoui SM*, El Azhari A, Rghioui M (Cheikh Khalifa International University Hospital, Morocco); Khya E (Moulay Youssef, Morocco); Ghannam A*, Souadka A*, El Ahmadi B, Belkhadir ZH, Majbar MA, Benkabbou A, Mohsine R (Institut National D'oncologie, Morocco); Oudrhiri MY*, Bechri H, Arkha Y, El Ouahabi A (Centre Hospitalier Universitaire Ibn Sina Rabat, Morocco); Frima H*, Bachiri S, Groen LC (Noordwest Ziekenhuisgroep, Alkmaar, Netherlands); Verhagen T*, ter Brugge FM (Ziekenhuisgroep Twente, Almelo, Netherlands); Scheijmans JCG, Boermeester MA*, Hompes R*, Meima-van Praag EM, Sharabiany S (Amsterdam UMC, University of Amsterdam, Netherlands); Borgstein ABJ, Gisbertz SS*, van Berge Henegouwen MI* (Amsterdam UMC VUMV, Netherlands); Gans S*, Van Duijvendijk P, Herklots T, De Hoop T, De Graaff MR, Sloothaak D, Bolster-van Eenennaam M, Baaij J, Klinkenbijl JHG (Gelre ziekenhuis, Apeldoorn, Netherlands); Van Eekeren R, Spillenaar Bilgen EJ (Rijnstate, Arnhem, Netherlands); Van der Burg SW, Harlaar NJ*, Jonker FHW (Rode Kruis Ziekenhuis, Beverwijk, Netherlands); Vermaas M, Voigt KR (Ijsselland Ziekenhuis, Capelle aan den Ijssel, Netherlands); Nellensteijn D*, Bensi EAB, (Curacao Medical Center, Willemstad, Curaçao, Curaçao, Netherlands); Posma-Bouman L* (Slingeland Ziekenhuis, Netherlands); Van Sambeek M*, Holscher M (Catharina Ziekenhuis, Eindhoven, Netherlands); Van den den Broek WT (St. Anna Ziekenhuis, Geldrop , Netherlands); HKruijff S*, De Vries JPPM, Steinkamp PJ, Jonker PKC, Van der Plas WY, Bierman W, Janssen Y (University Medical Center Groningen, Netherlands); Franken J, Oosterling S* (Spaarne Gasthuis, Netherlands); Boerma EG*, Schweitzer D, Keulen MHF, Ketting S (Zuyderland MC, Sittard/Heerlen, Netherlands); Wegdam JA, Vries Reilingh TS de, Schipper E, Teeuwen PHE (Elkerliek Ziekenhuis, Helmond, Netherlands); Hendriks ER, Van Geloven AAW (Tergooi Hospital, Hilversum, Netherlands); Emous M*, Poelstra R, Teunissen M (Medisch Centrum Leeuwarden, Leeuwarden, Netherlands); Gerritsen SL, Boerma D, (st. Antonius Ziekenhuis, Nieuwegein, Netherlands); De Reuver PR*, Thunnissen F, Vermeulen BAM, Groen A, (Radboud Universitair Medisch Centrum, Nijmegen, Netherlands); Van Ginhoven TM*, Viëtor CL, van der Oest MJW (Erasmus Medisch Centrum, Rotterdam, Netherlands); Vriens PWHE*, Houwen T, J Heisterkamp (Elisabeth TweeSteden Ziekenhuis, Tilburg, Netherlands); van Petersen AS, van der Meij W, Stevens CT (Bernhoven, Uden, Netherlands); Pronk A, Bakker WJ (Diakonessenhuis, Utrecht, Netherlands); Richir MC*, Vriens MR, Filipe MD, (University Medical Center Utrecht, Utrecht, Netherlands); Uittenbogaart M, Leclercq WKG*, Sijmons JML, Vancoillie PJ (Maxima Medical Center, Netherlands); Konsten J*, Van Heinsbergen M (VieCuri Medisch Centrum, Netherlands); Dekker NAM, den Boer FC (Zaans Medisch Centrum, Zaandam, Netherlands); Akinmade A*, Adeyeye A, Enoch E, Fayose S (Afe Babalola University Multi-System Hospital, Nigeria); Okunlola AI*, Adeniyi AA, Adeyemo OT, Adebara IO, Bakare A, Babalola OF, Abiyere OH, Banjo OO (Federal Teaching Hospital, Ido Ekiti, Nigeria); Olori S*, Akaba OG, Agida ET, Abdullahi IH (University of Abuja Teaching Hospital, Nigeria); Egbuchulem IK, Olulana D, Lawal TA*, Ogundoyin O, Oyelakin OA*, Nwaorgu OG, Sule SO (University College Hospital, Nigeria); Makwe CC, Afolabi BB, Seyi-Olajide JO*, Ademuyiwa AO, Bode CO, Atoyebi O, Elebute OA, Okunowo AA (Lagos University Teaching Hospital, Nigeria); Williams OM*, Eke NG, Oshodi OA, Faboya OM, Adeniran AS, Omisanjo OA, Oshodi YA,

Ogunyemi AA, Atobatele KM (Lagos State University Teaching Hospital, Nigeria); Adeyeye A, Aremu I, Olasehinde O, Abdur-Rahman L*, Bello J, Popoola A, Sayomi TO, Raji HO, Adeleke N, Lawal B, Habeeb O, Agodirin O (University of Ilorin Teaching Hospital, Nigeria); Tolani MA*, Sholadoye TT*, Nwabuoku SE, Abubakar M (Ahmadu Bello University Teaching Hospital, Nigeria); Risteski T*, Cvetanovska Naunova V, Jovcheski L, Lazova E (University Clinic for Pediatric Surgery, North Macedonia); Agledahl I* (Hammerfest Hospital, Norway); Breuer RG* (Soerlandet Hospital Kristiansand, Kristiansand, Norway); Massoud J* (Khoula Hospital, Oman); Waqar SH*, Rashid I, Ayubi A (Pakistan Institute of Medical Sciences, Pakistan); Bhatti ABH* (Shifa International Hospital, Pakistan); Younis MU (Jinnah post graduate medical center, Karachi, Pakistan); Ghouri A, Ayub B, Sayyed RH* (Patel Hospital, Pakistan); Saleem A, Turk K*, Alvi A, Abassy J*, Khan S, Arshad M, Ahmed K, Siddiqui T, Pirzada A (Aga Khan University, Pakistan); Kerawala AA*, Jamal A (Cancer Foundation Hospital, Pakistan); Rai L*, Nafees Ahmed R, Memon AS* (Dr Ruth K.m. Pfau Civil Hospital, Pakistan); Qureshi AU*, Ayyaz M*, Umar M, Butt U*, Kashif M, Khan WH*, Waris Farooka M*, Wasim T* (Services Hospital Lahore, Pakistan); Talat N,Tahir W, Naseem J (The Children's Hospital & The Institute of Child Health Lahore, Lahore, Pakistan); Akbar A*, Afroze S, Ali L,Sultan A, Ali HB (Doctors Hospital, Pakistan); Janjua MH*, Janjua A, Asghar S, Farooq MS, Sarwar MZ, Naqi SA, Gondal KM (King Edward Medical University, Mayo Hospital, Lahore, Pakistan); Bukhari SI* (Lady Reading Hospital, Pakistan); Tariq M (North West General Hospital and Research Centre, Pakistan); Javed S*, Yaqoob E, Ashraf M, Mahmood U, Raja Shabbir K (Holy Family Hospital, Pakistan); Abukhalaf SA, Amro A (Palestine Medical Complex, Palestine); Cabada Lee JM (Complejo Hospitalario Caja Seguro Social, Panama); Aguilar A (Hospital De Especialidades Pediatricas Css, Panama); Rodriguez E (Hospital Santo Tomas, Panama); Castillo K (Hospital Irma De Lourdes Tzanetatos CSS, Panama); Cukier M*, Rodriguez-Zentner H, Arrue E (Pacifica Salud Hospital, Panama); Isaacs Beron R (Hospital Regional Rafael Hernandez Css, Panama); Rodríguez Gonzalez A (Hospital de Clínicas, II Cátedra de Clínica Quirúrgica, Universidad Nacional de Asunción, Asuncion, Paraguay); Panduro-Correa V*, Cornelio DK (Hospital Hermilio Valdizan Medrano, Peru); Otiniano Alvarado CE*, Caballero Sarabia VD (Arzobispo Loayza National Hospital, Peru); Vasquez-Ojeda XP, Lizzetti-Mendoza G, Niquen-Jimenez M, Shu-Yip SB, León Palacios JL, Borda-Luque G* (Cayetano Heredia National Hospital, Peru); Zegarra SA*, Huamán Egoávil E, Suazo Carmelo C (Guillermo Almenara National Hospital, Peru); Castro de la Mata R*, Rivas D, Targarona J (Clínica Delgado Auna, Peru); Trujillo Y*, Olivera Villanueva M (Hospital Nacional Daniel Alcides Carrión, Peru); Lahoud-Velaochaga A*, Cabillas K, Castañeda W, Colina Casas J (Hospital Nacional Sergio E. Bernales, Lima, Peru); Betalleluz Pallardel J*, Camacho Zacarías F, Vélez Segura E, Cruz Condori DL (Hospital De Emergencias Jose Casimiro Ulloa, Peru); Huamán E* (Hospital Guillermo Kaelin de la Fuente, Lima, Peru); Ugarte Oscco R, Vergel Cabrera C, Huamán Egoávil E (Hospital Emergencia Ate Vitarte, Lima, Peru); Carpio Colmenares YT*, García Barrionuevo LA, Cárdenas Ruiz de Castilla D, Mansilla Doria P, Li Valencia MR (Clínica El Golf - Sanna, Peru); Salazar A, Sarmiento A, Díaz C, Morales E, Ore E, Zegarra H, Siccha J, Guardia M, Sandoval M, Mendiola GC*, Mimbela M (Hospital Santa Rosa De Lima, Peru); Diaz-Ruiz R*, Zeta LA, Cordova-Calle E, (Hospital Regional de Piura, Jose Cayetano Heredia , Peru); Nuñez HM (Hospital II-2 Tarapoto, Tarapoto, Peru); Ortiz-Argomedo MR* (Hospital Belén de Trujillo, Trujillo, La Libertad, Peru); Caballero-Alvarado J, Salazar-Tantaleán A, Espinoza-Llerena R (Hospital Regional Docente de Trujillo, Trujillo, Peru); Aliaga-Ramos M* (Hospital Alberto Hurtado Abadia, La Oroya - Yauli, Peru); Asodisen O*, Jabagat E, Tedoy CM (Cebu City Medical Center, Cebu City, Philippines); Ramos RA, Lopez MPJ*, Violago KLE, Aram R (Ospital Ng Makati, Philippines); Carlos Santos P, Filarca RF*Carlos A*, Santos P, Filarca RL* (Medical Center Manila-ManilaMed, Manila, Philippines); Domingo EJ, Khu KJO, Lapitan MC, Sacdalan MDP*, Kho MJN, Baticulon RE, Bravo SLR (Philippine General Hospital, University of The Philippines Manila, Philippines); Cueto, MAC*, Ramos, CL*, Fuentes, JR (José R. Reyes Memorial Medical Center, Manila, Philippines); Sadian H, Gumarao A, Barraquio A, Cruz EM, Gonzales AD (Pasig City General Hospital, Philippines); Reyes JAS*, Salud JA, Tancinco EG, Rivera RD, Lim JA (The Medical City, Philippines); Barcelon JC*, Chiu JA, Carballo MI (Cardinal Santos Medical Center, Philippines); Major P*, Gawron I*, Jach R* (Jagiellonian University Medical College, Poland); Borges F*, Matos Costa P, Henriques S, Rodrigues SC (Hospital Garcia de Orta, Portugal); Gonçalves N* (Hospital De Braga, Portugal); Curvas JM (Hospital de Bragança, Bragança, Portugal); Cabeleira A, Branco C, Serralheiro P*, Alves R, Teles T (Hospital De Cascais - Dr. José De Almeida, Portugal); Lázaro A*, Canhoto C, Simões J, Costa M, Almeida AC, Nogueira O, Oliveira A, Athayde Nemésio R, Silva M, Lopes C, Amaral MJ, Valente da Costa A, Andrade R, Martins R,Guimarães A, Guerreiro P, Ruivo A, Camacho C, Duque M, Santos E, Breda D, Oliveira JM, De Oliveira Lopez AL, Garrido S, Colino M, De Barros J, Correia S, Rodrigues M (Centro Hospitalar E Universitário De Coimbra, Portugal); Cardoso P*, Martins R, Teixeira J, Soares AP, Morais H*, Pereira R, Revez T, Manso MI, Domingues JC, Henriques P, Ribeiro R, Ribeiro VI, Cardoso N, Sousa S, Martins dos Santos G (Centro Hospitalar Universitário do Algarve - Unidade De Faro, Portugal); Carvalho L, Osório C, Antunes J, Lourenço S, Balau P, Godinho

M, Pereira A (Centro Hospitalar Entre o Douro e Vouga, Santa Maria da Feira, Portugal); Silva N*, Kam da Silva Andrade A, Pereira Rodrigues A, Borges N*, Correia J, Vieira I, Ribeiro T, Catarino J, Correia R, Pais F, Carreira Garcia R, Bento R, Cardoso J, Luis M, Santos E, Henriques J, Patena Forte J, Maciel J, Pinheiro Santos J, Silva M, Silva TP, Branquinho A (Centro Hospitalar Universitário Lisboa Central, Portugal); Caiado A* (Instituto Português De Oncologia De Lisboa Francisco Gentil, Portugal); Miranda P, Garrido R*, Peralta Ferreira M, Ascensão J, Costeira B, Cunha C, Rio Rodrigues L, Sousa Fernandes M, Azevedo P, Ribeiro J, Lourenço I, Gomes H, Mendinhos G*, Nobre Pinto A (Hospital Beatriz Angelo, Portugal); Ribeiro A, Gil CG, Lima-da-Silva C, Pereira C, Tavares F, Ferraz I, Almeida JI, Marialva J, Lopes L, Costa MJMA, Nunes-Coelho M, Teixeira MJ, Machado N, Alfonso JP, Saraiva P, Silva RL, Santos R, Almeida-Reis R*, Correia-de-Sá T, Fernandes V, Almeida-Pinto J, Gonçalves JP (Centro Hospitalar do Tâmega e Sousa, Portugal); Santos-Sousa H*, Cavaleiro S, Leite-Moreira AM, Pereira A, Pereira-Neves A, Faria CS, Monteiro JM, Nogueiro J, Sampaio-Alves M, Magalhães Maia M, Vieira P, Pina-Vaz T, Jácome F, Devezas V, Almeida A, Silveira H, Vaz S, Castanheira Rodrigues S (Centro Hospitalar E Universitário De São João, Portugal); Costa Santos D, Grilo JV, Abreu da Silva A*, Claro M, Deus AC (Hospital Do Litoral Alentejano, Portugal); Branquinho R* (Centro Hospitalar Medio Tejo, Portugal); Santos PMDD*, Patrício B, Vieira Paiva Lopes AC (Hospital De Torres Vedras - Centro Hospitalar Do Oeste, Portugal); Mendes JM, Carvalho MF, Oliveira CM* (Centro Hospitalar do Médio Ave, Portugal); Tojal A*, Pinto J (Centro Hospitalar Tondela-Viseu, Portugal); Abutaka A, Zarour A*, Abdelkareem M, Ali SM, Al Tarakji M, Alfkey R, Mukhtar K, Wani IR, Singh R, Ahmed K, Bouchiba N, Mahdi H, Abdelaziem Mustafa S, Al Ansari A (Hamad General Hospital, Qatar); Drasovean R, Caziuc A (Clinica Chirurgie I, Spitalul Clinic Judetean de Urgenta, Cluj-Napoca, Romania); Galliamov E, Agapov M*, Kakotkin V, Semina E, Kubyshkin V, Kamalov A (Moscow Research and Educational Center, Lomonosov Moscow State University, Russian Federation); Efetov SK, Kochetkov VS, Garmanova T, Tsarkov P*, Tulina I, Rodimov S, Markaryan D, Kazachenko E (Clinic of Coloproctology and Minimally Invasive Surgery, Sechenov Medical State University, Moscow, Russian Federation); Yanishev A*, Abelevich A, Bazaev A, Kokobelyan A, Zarubenko P (Privolzhsky Research Medical University, Nizhny Novgorod Regional Clinical Hospital, Russian Federation); Zakharenko A*, Novikova A (Pavlov First State Medical University of St. Petersburg, Russian Federation); Kim G*, Shmatov D, Stoliarov M, Kamenskikh M (Saint Petersburg State University Hospital, Saint Petersburg, Russian Federation); Nambi G (University Hospital, Saudi Arabia); Almulhim AS, Madkhali T, Alzouhir A, Alissa A (King Fahad Hospital Hofuf, Saudi Arabia); Alameer E*, Badedi M, Alnami AQ, Darraj H (Jazan University - King Fahd Central Hospital - Sabia General Hospital - Baish General Hospital, Saudi Arabia); Alkhuzaie A*, Khadwardi F, Abualjadayel M, Tashkandi W (King Abdulaziz Hospital and Oncology Center, Saudi Arabia); Farsi A, Malibary N*, Trabulsi N, Farsi S (King Abdulaziz University Hospital, Saudi Arabia); Said bayazeed A*, Nasser M*, Siddiqui MS*, Al Awwad S* (King Fahad General Hospital, Saudi Arabia); Alshahrani M, Alsharif F, Fahmi MW (Aseer Central Hospital, Saudi Arabia); Gudal A*, Alasmari A, Alqahtani S (King Abdullah Medical Complex - Jeddah, Saudi Arabia); Majrashi S*, Mashat A, Al Raddadi R (East Jeddah General Hospital, Saudi Arabia); Alharbi A*, Nasser Y, Hamayel H, Alhojaili A, Aljohani R (King Fahad General Hospital, Saudi Arabia); Sogair O* (Ohud Hospital, Saudi Arabia); Alfarhan O, Alzahrani A, Alzomaili B*, Tashkandi W (Hera General Hospital, Saudi Arabia); A Azab M* (King Abdullah Medical City Makkah, Saudi Arabia); Alotaibi M*, Maashi A, Zowgar A, Alsakkaf M (King Faisal Hospital, Saudi Arabia); Alnemary M*, Khayat S, Felmban S, Almhmadi A (Alnoor Specialist Hospital, Saudi Arabia); Alqannas M, Cortés Guiral D, Alyami M*, Elawad A (King Khalid Hospital, Najran, Saudi Arabia); Alhefdhi A*, Alresaini F, Kurdi W, Tulbah M, Aldakheel M, Alsahan N, Koussayer S, Elsheikh H, Al-qattan M, Alshanafey S, Rafique A, Mahabbat N, Saeed B (King Faisal Specialist Hospital, Saudi Arabia); Al-Kharashi E*, Alsowaina K, Arab N, Aljaber F, Al Hasan I, Alghamdi A, Badahdah F, Alghuliga A, Abdulfattah F, Alanazi F, Albaqami F, Alsuhaibani A (Prince Sultan Military Medical City, Saudi Arabia); AlFakhri A*, Alqasem S, Alajaji N (King Fahad Medical City, Saudi Arabia); Nouh T*, Bin Nasser A, Alowais J, Alburakan A, Alamri O, Albdah A, Alawi K, Alshalhoub M (King Saud University, Saudi Arabia); ElSanhoury K*, Almofarreh A, Ibrahim S, Elshafie H, Osman I, Guzman T, Mutair H, Siddiqui A, Chowdhury S*, Alghamdi R, Almutrafi S, Alfaifi J, D'Souza J, Alshitwi A, Alkreedees N, Alramadhan M, Alshehri M (King Saud Medical City, Saudi Arabia); Alzahrani A*, Alobaysi S, Badr H, Alshahrani A (Security Forces Hospital, Saudi Arabia); Alshehri A*, Alrashed M, Altahan T, Alsabahi T, Alhossaini R, Sbaih M (Prince Mohammed Bin Abdulaziz Hospital, Saudi Arabia); Alalawi Y, Alnwijy K, Al Ayed A (King Salman Armed Forces Hospital, Tabouk, Saudi Arabia); Ghedan S, Alharthi R, Awad S*, I Sharara M, Abdelrhman S, Althobaiti W (King Faisal Medical Complex, Saudi Arabia); Srbinovic L, Perovic M, Mikovic Z, Nikolic B, Vasiljevic M, Pazin V, Mandic Markovic V, Dimitrijevic D, Zecevic N (Clinic for Gynecology and Obstetrics Narodni Front, Serbia); Gregoric P*, Micic D, Loncar Z, Doklestic K, Ivancevic N (Clinic for Emergency Surgery, Emergency Center, Clinical Center of Serbia, Serbia); Djukic V*, Stojakov D, Ilic R, Savic P, Pijanovic N, Milanovic M, Radosavljevic M, Dejanovic T, Kostic M,

Paskas J, Bojic S, Stevanovic P, Djuric M (KBC Dr Dragisa Misovic-Dedinje, Serbia); Kadija M*, Tulic G, Glisovic Jovanovic I (Clinic for Orthopaedic Surgery and Traumatology, Clinical Center of Serbia, Belgrade, Serbia); Lieske B* (National University Hospital, Singapore, Singapore); Kayombo E, Kruger I, De Kock M, Malan A, Ferreira C, Du Preez H, Mulder W, Noel C*, Le Grange S, Lusawana O (Universitas Academic Hospital Complex, South Africa); Kies C, Steyn E, Janson J, Buitendag JJP, Chu K*, Mihalik M, Nel R, Naidoo S (Tygerberg Hospital, South Africa); Kloppers C*, Nel D, Jonas E, Pickard H, Bernon M, Almgla N*, Rayamajhi S, Mugla W* (Groote Schuur Hospital, South Africa); Carapinha C (Netcare Clinton Hospital, Johannesburg, South Africa); Hyman GY, Fourtounas M, Moore R* (Chris Hani Baragwanath Academic Hospital, South Africa); Sánchez Mozo A (Complejo Hospitalario Universitario De Albacete, Spain); Aguado Lopez H* (Hellin Hospital, Spain); Zárate Pinedo A (Hospital Germans Trias i Pujol, Badalona, Spain); Jimenez Toscano M*, Alonso de la Fuente N, Mancebo G, Cecchini L, Munarriz M, Cazador Labat M, López Campillo A, Martorell P, Espinosa CA (Hospital Del Mar, Spain); Caja Vivancos P*, Villalabeitia Ateca I*, Prieto Calvo M, Marín H, Martin Playa P*, Gainza A, Aragon Achig EJ, Rodriguez Fraga A, Melchor Corcóstegui I, Mallabiabarrena Ormaechea G*, Garcia Gutierrez JJ, Barbier L, Pesántez Peralta MA*, Jiménez Jiménez M, Municio Martín JA, Gómez Suárez J, García Operé G, Pascua Gómez LA, Oñate Aguirre M (Hospital Universitario Cruces, Spain); Fernandez-Colorado A, De la Rosa-Estadella M*, Gasulla-Rodriguez A, Serrano-Martin M, Peig-Font A, Junca-Marti S, Juarez-Pomes M, Garrido-Ondono S, Blasco-Torres L, Molina-Corbacho M, Maldonado-Sotoca Y, Gasset-Teixidor A, Blasco-Moreu J (Corporació Sanitària Parc Taulí, Spain); Gomez Fernandez L*, Cayetano Paniagua L (Consorci Sanitari De Terrassa, Spain); Izquierdo O, Ventura D, Castellanos J (Parc Sanitari Sant Joan de Déu, Sant Boi de Llobregat, Barcelona, Spain); Ballester Vazquez E, Sanchez Lopez A*, Balague Ponz C, Targarona Soler EM, Sanchez Cabús S, Molina Santos V, Gonzalez Lopez JA, Medrano Caviedes R, Moral Duarte A (Hospital De La Santa Creu I Sant Pau, Spain); Espin-Basany E*, Pellino G, Blanco-Colino R (Vall D'hebron University Hospital, Spain); Turrado-Rodriguez V*, Lacy AM, de Lacy FB, Morales X, Carreras-Castañer A*, Torner P, Jornet-Gibert M, Balaguer-Castro M, Renau-Cerrillo M, Camacho-Carrasco P, Vives-Barquiel M, Campuzano-Bitterling B, Gracia I, Pujol-Muncunill R (Hospital Clínic de Barcelona, Spain); Martin-Sole O*, Rubio-Palau J, Tarrado X, Garcia-Aparicio L, De Haro Jorge I, Martin A, Rojas-Ticona J, Perez-Bertolez S, Cuesta Argos M, Capdevila Vilaro B, Coronas Soucheiron M, Riba Martinez M, Saura Garcia L, Prat Ortells J, Bejarano Serrano M, Parri P, Massaguer C, Vicario F, Palazon Bellver P, Moraleda Gudayol I (Hospital Sant Joan de Deu de Barcelona, Spain); Lara A, Escobar D, Arrieta M, Garcia de cortazar U*, Villamor Garcia I (Hospital Universitario De Basurto, Spain); Landaluce-olavarria A*, Gonzalez De miguel M, Fernández Gómez Cruzado L, Begoña E, Lecumberri D (Hospital Urduliz, Spain); Acosta Mérida MA*, Yepes Cano AF* (Hospital Universitario De Gran Canaria Doctor Negrín, Spain); Estaire Gómez M*, Padilla-Valverde D*, Sánchez-García S, Sanchez-Pelaez D, Jimenez Higuera E, Picón Rodríguez R, Fernández Camuñas À, Martínez-Pinedo C, Garcia Santos EP, Muñoz-Atienza V, Moreno Pérez A, López de la Manzanara Cano CA (Hospital General Universitario De Ciudad Real, Spain); Ugarte-Sierra B*, Ibáñez-Aguirre FJ, De Andres Olabarria U, Fernández Pablos FJ, Durán Ballesteros M, Sanz Larrainzar A (Hospital Universitario De Galdakao, Spain); Jiménez Carneros V*, Valle Rubio A, Alonso-Lamberti L, Salazar A, García-Quijada J, Leon R, Rodriguez JL, Jimenez Miramón J, Jover JM, Martín Salamanca MB*, Assaf M, Pérez Simón V, Landeo Agüero SA, Baeza Pintado N, Huertas Fernandez MA, Carabias A (Getafe University Hospital, Spain); Sosa MV*, Lora-Cumplido P, Lanuza L (Hospital de Cabueñes, Spain); Galipienso Eri M, Garcia Montesino JD, Dellonder Frigolé J, Noriego Muñoz D* (Hospital Universitari De Girona Dr. Josep Trueta, Spain); Navarro-Sánchez A* (Complejo Hospitalario Universitario Insular-Materno Infantil de Gran Canaria, Spain); Enjuto D*, Perez Gonzalez M, Díaz Peña P, Gonzalez J, Marqueta De Salas M, Martinez Pascual P, Rodríguez Gómez L, Garcés García R, Ramos Bonilla A, Herrera-Merino N, Fernández Bernabé P, Cagigal Ortega EP, Hernández I, García de Castro Rubio E, Cervera I (Severo Ochoa University Hospital, Spain); Sánchez-Guillén L, Fernández-Candela A, Curtis-Martínez C, Soler-Silva Á, Oller Á, Triguero-Cánovas D, Bosch-Ramírez M, Lillo C, Lario S, Arroyo A (Universidad Miguel Hernández, Elche, Alicante, Spain); Espino Segura-Illa M, Sánchez Aniceto G*, Castaño-Leon AM*, Jimenez-Roldan L, Delgado Fernandez J, Pérez Núñez A, Lagares A, Garcia Perez D, Santas M, Paredes I, Esteban Sinovas O, Moreno-Gomez L, Rubio E*, Vega V, Vivas Lopez A, Labalde Martinez M, García Villar O, Pelaéz Torres PM, Garcia - Borda J, Ferrero Herrero E, Gomez P, Eiriz Fernandez C, Ojeda-Thies C*, Pardo Garcia JM (12 De Octubre University Hospital, Spain); Di Martino M*, De la Hoz Rodriguez A, García Septiem J, Maqueda González R, Delgado Búrdalo L, Correa Bonito A, Martin-Perez E (Hospital Universitario De La Princesa, Spain); García Villayzán JE*, Albi Martin B (Fundación Jimenez Diaz University Hospital, Spain); Lozano Lominchar P, Martin L, Fernadez M, Rey-Valcarcel C*, Tousidonis M, Martin-Albo Caballero L, Lowy A, Alonso Ortuño P, Ayuso Herrera E, Caño Velasco J, Aragon-Chamizo J, Perez Diaz MD, Mateo-Sierra O, Quintana-Villamandos B, Barrio JM, Fanjul M, Sanchez- Perez C, Fernandez ML, Hernandez-

Kakauridze S, Rio J (Hospital General Universitario Gregorio Marañón, Spain); Díaz Pérez D, Serrano González J*, Colao García L, Gutierrez Samaniego M, Hernandez Bartolome MA, Galindo Jara P, Esteban Agustí E (Hospital Universitario De Torrejón De Ardoz, Spain); Ripollés-Melchor J*, Abad-Motos A, Abad-Gurumeta A, Martínez-Hurtado E, Ruiz-Escobar A (Infanta Leonor University Hospital, Spain); Brogly N, Guasch E, Hernandez Gutierrez A, Bartha Rasero JL, Perez Y, Garcia-Pineda V, Gracia M, Siegrist Ridruejo J, Diestro MD, Sanchez-Mendez JI, Marti C, Melendez M, Moreno-Palacios E, Loayza A, Frias L, Zapardiel I*, Rubio-Perez I*, Prieto Nieto MI, Guevara J, Gegundez Simon A, GortazarS, Chavarrias N, Alvarez E, Saavedra J, Ramos-Martin P, Urbieta A, Gomez Rivas J, Toribio-Vázquez C, Yebes A (Hospital Universitario La Paz, Spain); Hernández-García M, Losada M*, Diéguez B, García-Conde M, Alonso Poza A (Hospital Universitario Del Sureste, Spain); Marquez L*, Becerra R, Martin M, Jorgensen T (Hospital Central De La Cruz Roja San Jose Y Santa Adela, Spain); Mugerza JM*, Dziakova J, Sánchez del Pueblo C, Saez Carlin P, Camarero E, Picazo S, Pizarro MJ, Avellana R, Catalán V, Lopez Antoñanzas L, Cano O, Anula R, Sanz Lopez R, Sanz Ortega G, Garcia Alonso M, Torres AJ, Martin Antona E,Garcia Botella S (Hospital Clínico San Carlos De Madrid, Spain); Ramos D*, Barranquero AG, Ocaña J, Núñez J, Cerro Zaballos C (Hospital Universitario Ramón Y Cajal, Spain); Crego-Vita D*, Huecas-Martinez M (Hospital Central De La Defensa Gomez Ulla, Madrid, Spain); Diez Alonso M*, Mendoza-Moreno F, Vera Mansilla C, Ovejero Merino E, Hernandez P, Blazquez Martin A, Ruiz Grande F, Morales Palacios N*, Garcia-Loarte Gomez E, Garca Rico E (Hospital Universitario Principe De Asturias, Spain); Minaya-Bravo AM*, San Miguel Méndez C, Galvan Pérez A, Gonzalez-Gonzalez E, Robin Valle de Lersundi A, Calcerrada Alises E, García-Ureña MA, Cruz Cidoncha A (Hospital Del Henares. Universidad Francisco de Vitoria. , Spain); Troncoso Pereira P*, Alcaide Matas F, García Pérez JM (Hospital Mateu Orfila, Spain); Muñoz Vives JM, Osorio A, Gómez Díaz CJ, Guariglia CA, Soto Montesinos C*, Sanchon L, Xicola Martínez M, Guàrdia N, Collera P, Diaz Del Gobbo R, Sanchez Jimenez R, Farre Font R, Flores Clotet R (Fundació Althaia - Xarxa Assistencial Universitària de Manresa, Spain); Calvo Espino P*, Guillamot Ruano P (Hospital Universitario De Móstoles, Spain); Rey-Biel J*, Pingarrón-Martin L, Ruiz Martin I and Moliner Sanchez C (Rey Juan Carlos University Hospital, Spain); Carrasco-Prats M*, Giménez-Francés C, Ruiz-Marín M, Fernández-López AJ, García-Escudero D, García-Porcel V, Lax-Pérez R, Sánchez-Robles M, Valero-Soriano M, Medina-Manuel E, García-Soria V, Gurrea-Almela E, Marco-Garrido A, Martínez-Alonso JA, González-Valverde FM, Fernández-Fernández PV, Sánchez- Rodríguez C (Hospital General Reina Sofía, Spain); Aguilar-Jimenez J*, Baeza-Murcia M, Aguayo-Albasini JL (Morales Meseguer University Hospital, Murcia, Spain); Nicolás-López T, Alconchel F* (Hospital Clínico Universitario Virgen de la Arrixaca (IMIB-Arrixaca), Spain); Fernández Martínez D, Solar-Garcia L, García Flórez LJ* (Hospital Universitario Central De Asturias (Huca), Spain); Llaquet Bayo H* (Hospital de Palamós-SSIBE, Spain); Pujol-Cano N, Segura-Sampedro JJ*, Soldevila-Verdeguer C, Jeri-McFarlane S, Gil-Catalan A, Craus-Miguel A, Cruz L, Valente P, Afonso-Garcia M, Ferrer-Inaebnit E, Oseira-Reigosa A, Fernandez-Vega L, Villalonga-Ramirez B, Gonzalez Argente FX (Son Espases University Hospital, Spain); Mora-Guzmán I* (Hospital Santa Bárbara, Spain); Landete Molina FJ*, Morera Ocón FJ, Canelles Corell E (Hospital General Asociado Universitario de Requena, Spain); Gavaldà Pellicé MT*, Salinas Peña JR, Cavallé Busquets P (Hospital Universitari Sant Joan, Reus, Spain); Trebol J*, Sánchez-Casado AB, Munoz-Bellvis L (Complejo Asistencial Universitario De Salamanca, Spain); Pérez-Sánchez LE*, Concepción Martín V, Díaz García A, Vallve-Bernal M (Hospital Universitario Nuestra Señora De Candelaria, Spain); Calvo Rey A, Prada hervella GM*, Dos Santos Carregal L, Rodriguez Fernandez MI, Freijeiro M, El Drubi Vega S (Hospital Clinico Universitario De Santiago De Compostela (Chus), Spain); Picardo AL, Cuadrado-Garcia A, Serralta de Colsa D, Rojo Lopez JA, Sanchez Cabezudo Noguera F, Ortega Vazquez I*, Garcia-Sancho Tellez L, Mato P, Heras Aznar J (Infanta Sofia University Hospital, Spain); Jimeno Fraile J*, Morales-Garcia D, Carrillo-Rivas M, Toledo Martínez E, Pascual À (Marqués De Valdecilla University Hospital, Spain); Senent-Boza A*, Sánchez-Arteaga A, Benítez-Linero I, Manresa-Manresa F, Tallón-Aguilar L, Melero-Cortés L, Fernández-Marín MR, Durán-Muñoz-Cruzado VM, Ramallo-Solís I, Beltrán-Miranda P, Pareja-Ciuró F, Antón-Eguía BT (Hospital Universitario Virgen Del Rocío, Spain); Oliva Mompean F, Gomez-Rosado J*, Reguera-Rosal J, Valdes-Hernandez J, Capitan-Morales L, del Toro Lopez MD (Hospital Universitario Virgen Macarena, Spain); Achalandabaso Boira M*, Memba Ikuga R, Abellán M, Sales R, Olona C, Jorba R (Hospital Universitari De Tarragona Joan XXIII, Spain); Hernandez Gutierrez J, Tébar Zamora A* (Complejo Hospitalario De Toledo, Spain); Sancho-Muriel J*, Cholewa H, Frasson M (Hospital Universitario Y Politécnico La Fe, Spain); Domenech J, Roselló Añón A*, Sangüesa MJ (Hospital Arnau De Vilanova, Spain); Moro-Valdezate D*, Garcés-Albir M*, Lopez F* (Hospital Clínico Universitario de Valencia, Spain); Bernal-Sprekelsen JC*, Catalá Bauset JC, Renovell Ferrer P, Martínez Pérez C, Gil-Albarova O, Gilabert Estellés J, Aghababyan K (Consorcio Hospital General Universitario, Spain); De Andrés-Asenjo B*, Beltrán de Heredia J, Vázquez- Fernández A, Ortiz de Solorzano-Aurusa FJ, Trujillo-Díaz J, Ruiz-Soriano M, Jezieniecki C, Gómez-Sanz T, Núñez-Del

For peer review only

Barrio H, Romero-De Diego A, García-Virto V, Aguado HJ (Hospital Clínico Universitario De Valladolid, Spain); Fernández Martín MT* (Hospital Medina Del Campo, Spain); Tejero-Pintor FJ*, Pérez-Saborido B, Choolani Bhojwani E, Acebes García F, Marcos-Santos P, Bueno Cañones AD, Sanchez Gonzalez J, Toledano M, Bailón M, Pacheco Sánchez D (Hospital Universitario Río Hortega, Spain); Paniagua Garcia Senorans M*, Sanchez-Santos R (Álvaro Cunqueiro Hospital, Spain); Vazquez Melero A*, Garcia D, Díez E, Herrero I, Soeda IM, Camuera M, Balluerca M, Sánchez-Rubio M, Paunero Vazquez P (Hospital Universitario Araba, Spain); Martinez-German A, Gracia-Roche C, Gascon-Ferrer I, Duque-Mallen V*, De Miguel-Ardevines MDC, Sanchez-Fuentes N, Santero-Ramirez MS, Matute-Najarro M, Herrero-Lopez M, Sanchez-Rubio M, Cantalejo-Diaz M, Gonzalez-Nicolas-Trebol MT, Saudi-Moro S, Jariod-Ferrer UM (Hospital Universitario Miguel Servet, Spain); Rivas R, Rivas F (Hospital Clinico Universitario Zaragoza, Zaragoza, Spain); Escartin J*, Blas Laina JL, Nogués A, Cros B, Talal El-Abur I, Garcia Egea J, Yanez C (Hospital Royo Villanova, Spain); Jayarajah U*, Ravindrakumar S, Rodrigo VSD, Arulanantham A, Bandara GBKD (District General Hospital Chilaw, Sri Lanka); Hamid HKS, Ali EE, Widatalla ABH, Bakheit I*, Awadelkarim M (Ibrahim Malik Teaching Hospital, Sudan); Ali karar AA (Al-Rajhi, Sudan); Saleh M (University of Gezira Hospital, Sudan); Taflin H* (Sahlgrenska University Hospital, Sweden); Myrelid P*, Amorim Braz L (Linköping University Hospital, Sweden); Hagander L*, Hambraeus M, Omling E, Salö M (Lund University, Skåne University Hospital,, Sweden); Arkani S*, Freedman J* (Danderyds Hospital, Sweden); Montan C, Lindqvist EK*, Elbe P, Hultgren R (Karolinska University Hospital, Sweden); Älgå A*, Nordberg M, Sandblom G (South General Hospital, Sweden); Rutegård M*, Holmner F, Sund M*, Löfgren N (Umeå University Hospital, Sweden); Tampakis A, Kollmar O, von Fluee M (Clarunis, University Hospital Basel, Basel, Switzerland); Balaphas A*, Toso C, Colucci N, Popeskou SG (Geneva University Hospitals, Switzerland); Gass M*, Scheiwiller A, Metzger J (Luzerner Kantonsspital, Switzerland); Gialamas E, Chevallay M, Sauvain M*, Dwidar O (Pourtales Neuchatel Hospital, Switzerland); Kiessling SY, Stoeckli SJ* (Kantonsspital St. Gallen, Switzerland); Mongelli F, Bernasconi M, Di Giuseppe M, Christoforidis D*, La Regina D, Arigoni M (Ente Ospedaliero Cantonale, Switzerland); Adamina M*, Peros G, Guglielmetti L, Solimene F, Giardini M, Bächler T, Crugnale AS (Kantonsspital Winterthur, Switzerland); Gutschow CA*, Turina M (Universitätsspital Zürich, Switzerland); Ersen O (Ankara University Medical School, Ankara, Turkey); Onan MA*, Kozan R (Gazi University Medical Faculty Hospital, Turkey); Erol T*, Dincer HA (Hacettepe University Hospital, Turkey); Yildiz A* (Yildirim Beyazit University Yenimahalle Training and Research Hospital, Turkey); Iflazoglu N, Yalkın O (Bursa City Hospital, Bursa, Turkey); Isik A* (Erzincan University Hospital, Turkey); Ozben V*, Aytac E, Aliyeva Z, Akaydin E, Ozmen BB, Baca B (Acibadem Atakent Hospital, Turkey); Altinel Y*, Calikoglu F, Tokocin M, Hacim NA, Akbas A, Meric S, Vartanoglu T, Yigitbas H, Ercetin C, Ercan G (Bagcilar Research and Training Hospital, Turkey); Ozgur I*, Keskin M (Istanbul University Faculty of Medicine, Istanbul, Turkey); Saracoglu KT*, Cimenoglu B, Demirhan R, Kale A, Simsek T, Gundogdu EC (Kartal Dr. Lutfi Kirdar Training and Research Hospital, Turkey); Abbasov A* (Liv Hospital Ulus, Turkey); Tanal M*, Citgez B, Bozkurt E, Yetkin SG, Mihmanli M (University of Health Sciences Sisli Hamidiye Etfal Training and Research Hospital, Turkey); Alhamed A, Ergun S*, Sanli AN, Velidedeoglu M, OZcelik k MF, Uludag SS*, Zengin AK, Cebi S, Demirkiran F, Bese T, Acikgoz AS, Kayan B, Aykanat Y, Mutlu D (Istanbul University - Cerrahpasa, Cerrahpasa Medical Faculty, Turkey); Göksoy B* (Sehit Prof.Dr. İlhan Varank Training and Research Hospital, Turkey); Kara Y*, Bozkurt MA, Kocatas A (Kanuni Sultan Suleyman Training and Research Hospital, Turkey); Öğücü H, Uslu G, Arican C, Tugmen C, Aydin C, Yesilyurt D, Avci EK, Kebapçı E, Kilinc G, Sert İ, Tuncer K, Akalin M, Emiroglu M, Demirli Atici S*, Salimoğlu S, Kaya T*, Kirmizi Y (University of Health Sciences Tepecik Training and Research Hospital, Turkey); Tatar OC*, Yüksel E, Güler SA, Yildirim A, Utkan NZ, Gözal K, Köken H, Yabas A (Kocaeli University Teaching Hospital, Turkey); Gonullu E, Altintoprak F, Akin E, Kamburoglu B, Capoglu R, Kucuk F, Demir H, Cakmak G, Firat N, Celebi F, Kocer B, Mantoglu B, Bayhan Z, Dikicier E (Sakarya University Faculty of Medicine, Turkey); Colak E*, Kucuk GO (Samsun Training and Research Hospital, Turkey); Karaman E*, KolusabA, Karaaslan O (Van Yuzuncu Yil University, Medical Faculty, Turkey); Majid I, Alshryda S* (Al Jalila Children's Speciality Hospital, United Arab Emirates); Abbas F*, Abbas FMA (Dubai Hospital, United Arab Emirates); Mohammed D*, Tahlak MA* (Latifa Women and Children Hospital, Dubai, United Arab Emirates); Yammahi A, Albaroudi AA, Elyafawi B, Saber A, Khansaheb H, Alsaadi H*, Alzarooni N* (Rashid Hospital, United Arab Emirates); Bekheit M*, Kamera BS, Elhusseini M, Sharma P, Ahmeidat A, Gradinariu G, Cymes W, Hannah A, Mignot G, Shaikh S*, Agilinko J (Aberdeen Royal Infirmary, United Kingdom); Angelou D, Neely D*, McCanny A, McAree B (Antrim Area Hospital, Northern Health and Social Care Trust, United Kingdom); Baldwin AJ, West R*, Gammeri E, Catton A, Marinos Kouris S (Stoke Mandeville, Wycombe General, United Kingdom); Pereca J*, Singh J (University Hospital Ayr, United Kingdom); Seymour Z*, Jones R*, Leeson S, Peevor R, Lala AK, Houlden C (Ysbyty Gwynedd, United Kingdom); Kahiu J*, Hossain N, Hosny S (Barnet General Hospital, United Kingdom); Patel P*, Handa S, Kaushal M, Kler A, Reghuram V, Tezas S (Furness General Hospital, United

Kingdom); Fairhurst K*, Yates C, Mitchell S, Bunni J, Richards S, George R, Lee SM, Phull J, Frost J*, Burnard S, Crowley R, Airey A (Royal United Hospitals Bath, United Kingdom); Bevan K*, Makin-Taylor R, Ong CS, Callan R, Bloom O (Bedford Hospital, United Kingdom); Aljanadi F, Moawad N , Jones M*, Gregg A, Jeganathan R* (Royal Victoria Hospital, Belfast, United Kingdom); Pachl M*, Martin B (Birmingham Children's Hospital, United Kingdom); Archer JE*, Odeh A, Siddaiah N (Royal Orthopaedic Hospital, United Kingdom); Singhal R*, Naumann DN, Karandikar S*, Syed A*, Tucker ON, Alam R, Kalkat M* (Heartlands Hospital, United Kingdom); Mak JKC, Kulkarni R, Sharma N, Nankivell P, Tirotta F, Parente A, Breik O, Kisiel A, Cato LD, Saeed S, Bhangu A*, Griffiths E*, Pathanki AM, Ford S*, Desai A*, Almond M*, Kamal M (Queen Elizabeth Hospital Birmingham, United Kingdom); Sundar S*, Leung EYL*, Kaur R, Brett-Miller C, Buruiana FE (Pan-Birmingham Gynaecological Cancer Centre, United Kingdom); Markose G, De Gea Rico A, Taib A*, Myatt D, Sulaiman Khaled A, Younis F* (East Lancashire Hospitals NHS Trust, United Kingdom); Sultana A*, Taggarsi M, Vitone L*, Lambert J, Vaz OP, Sarantitis I, Shrestha D, Timbrell S, Shugaba A (Royal Blackburn Hospital, United Kingdom); Quddus B, Law J, Bittar MN*, Creanga M, Elniel M, Youssef M*, Ali S, Qadri ST (Blackpool Victoria Hospital, United Kingdom); Brixton G,Findlay L, Klatte T Majkowska A, Manson J*, Potter R (Royal Bournemouth Hospital, United Kingdom); Oktseloglou V*, Mosley F*, De La Cruz Monroy MFI, Bobak P, Omar I, Ahad S, Langlands F, Brown V, Hashem M (Bradford Royal Infirmary, United Kingdom); Kennedy L*, Jaunoo S, Coomber E, Williams O (Royal Sussex County Hospital, United Kingdom); Shalaby M*, Rhodes HL (Bristol Royal Hospital for Children, United Kingdom); Williams A, Ridgway A, Pournaras D, Britton E, Lostis E, Ambler GK, Chu H, Hopkins J*, Manara J, Chan M, Doe M, Moon RDC, Lawday S, Jichi T, Singleton W (Southmead Hospital, United Kingdom); Main B, Maccabe T, Newton C*, Blencowe NS, Fudulu DP, Bhojwani D, Baquedano M, Caputo M*, Rapetto F, Flannery, O, Hassan A (University Hospitals Bristol and Weston NHS Foundation Trust, United Kingdom); Coonar A*, Aresu G, Smith C, Gearon D, Hogan J, Pradeep IS, Durio Yates H, Peryt A, Barrett-Brown ZM, King M, Ahmadi N, Jenkins D*, Moorjani N, Taghavi F, Wells F (Royal Papworth Hospital, United Kingdom); Hardie J, Page S, Anazor F, King SD, Luck J, Kazzaz S* (Frimley Park, Frimley Health NHS FT, United Kingdom); Mannion R, Stewart GD*, Ramzi J, Mohan M, Singh AA, Ashcroft J, Baker OJ, Coughlin P*, Davies RJ*, Durst AZED, Abood A, Habeeb A, Hudson VE, Kolias A, Lamb B, Luke L, Mitrasinovic S, Murphy S, Ngu AWT, O'Neill JR*, Waseem S, Wong K, Georgiades F, Hutchinson PJ*, Tan XS, Pushpa-rajah J, Colquhoun A, Masterson L, Abu-Nayla I, Walker C, Balakrishnan A*, Rooney S, Irune E, Byrne MHV, Durrani A (Addenbrooke's Hospital, Cambridge University Hospitals NHS Foundation Trust, United Kingdom); Simoes A, Eddy B, Streeter E, Ahmed I, Yao M*, Wang W, Djouani A, Tait-Bailey J, Thomas M, Hassan F, Kommu S (Kent and Canterbury Hospital, United Kingdom); Chopra S (University Hospital Llandough, United Kingdom); Richards T*, Sethuraman Venkatesan A, Combellack T*, Williams J, Tahhan G, Mohammed M, Kornaszewska M, Valtzoglou V, Deglurkar I*, Rahman M, Von Oppell U, Mehta D, Koutentakis M, Syed Nong Chek SAH, Hill G, Morris C*, Shinkwin M, Torkington J, Cornish J (University Hospital of Wales, United Kingdom); Houston R, Mannan S*, Ayeni F, Tustin H, Bordenave M, Robson A (Cumberland Infirmary, United Kingdom); Dovell G, Preece R, Rolland P* (Cheltenham General Hospital, United Kingdom); Miranda BH* (St Andrew’s Centre for Plastic Surgery and Burns, Chelmsford, United Kingdom); Sobti A, Khaleel A*, Unnithan A, Memon K, Pala Bhaskar RR, Maqboul F, Kamel F, Al-Samaraee A, Madani R*, Kumar L, Nisar P, Agrawal S* (Ashford and St Peter's Hospital, United Kingdom); Vimalachandran D*, Manu N, Eardley N, Krishnan E, Serevina OL, Martin E, Smith C, Jones A, Roy Mahapatra S, Clifford R (Countess of Chester Hospital, United Kingdom); Jones GP, Gardner A, Tripathi SS*, Greenhalgh MS (Lancashire Teaching Hospitals NHS Foundation Trust, United Kingdom); Matthews W, Mohankumar K, Khawaja I, Palepa A, Doulias T (Colchester University Hospital, United Kingdom); Gill C, Dunne N, Sarma DR, Godbole C, Carlos W, Tewari N*, Jeevan D, Naredla P, Khajuria A, Connolly H, Robertson S, Sweeney C, Di Taranto G, Shanbhag S, Dickson K, McEvoy K, Skillman J, Sait M, Al-omishy H, Baig M, Heer B (University Hospitals Coventry and Warwickshire NHS Trusts, United Kingdom); Brown A* (Darlington Memorial Hospital, United Kingdom); Ebrahim A, Alwadiya A, Goyal A*, Phillips A, Bhalla A*, Demetriou C, Grimley E, Theophilidou E, Ogden E, Malcolm FL, Davies-Jones G, Ng JCK, Mirza M, Hassan M, Elmaleh N, Daliya P, Williams S, Bateman A*, Chia Z (Royal Derby Hospital, United Kingdom); Premakumar Y, Jauhari Y, Koshnow Z, Bowen D, Uberai A, Hirri F, Stubbs BM* (Dorset County Hospital, United Kingdom); Crichton R*, Sonksen J, Aldridge K (Russell's Hall Hospital, Dudley, United Kingdom); McDonald C*, Manickavasagam J*, Ragupathy K*, Davison S, Dalgleish S*, McGrath N, Kanitkar R, Payne CJ, Ramsay L (Ninewells Hospital, United Kingdom); Ng CE*, Collier T, Khan K*, Evans R (University Hospital North Durham, United Kingdom); Brennan C, Henshall DE, Drake T*, Harrison EM*, Zamvar V, Tambyraja A, Skipworth RJE*, Linder G, McGregor R, Brennan P, Mayes J, Ross L, Smith S, White T, Jamjoom AAB, Pasricha R (Royal Infirmary of Edinburgh, United Kingdom); Gallagher K, Swan R, Paterson H*, Maeda Y, Kwok AMF, Tsiaousidou A, Vaughan-Shaw PG, Boyle C, Fernando D, Tham D, Leung S, Laird A* (Western General Hospital,

For peer review only

United Kingdom); Holme T, Abbott S, Razik A, Thrumurthy S, Steinke J, Baker M*, Howden D, Baxter Z, Osagie L, Bence M (Epsom & St Helier University Hospitals NHS Trust, United Kingdom); Fowler GE, Massey L, Rajaretnam N, Evans J, John J, Goubran A, Campain N, McDermott FD*, McGrath JS*, Ng M, Pascoe J, Phillips JRA, Daniels IR (Royal Devon and Exeter Hospital, United Kingdom); A'Court J, Konarski A, Faulkner G* (Royal Bolton Hospital, United Kingdom); Emerson H, Vejsbjerg K, Pearce L, McCormick W, Fisher A*, Singisetti K, Aawsaj Y, Barry C (Gateshead Health NHS Foundation Trust, United Kingdom); Bajomo O, Rizvi S, Grimes C*, Dusu K, Tint PY (Medway Hospital, United Kingdom); Kirk A*, Irvine V (Golden Jubilee National Hospital, United Kingdom); Lammy S*, O'Kane R (Royal Hospital for Sick Children, United Kingdom); Elliott L, Mccabe G, Holroyd D*, Jamieson NB (Glasgow Royal Infirmary, United Kingdom); Geddes A, McMahon J, McCaul J*, Al-Azzawi M, Aitken E*, Glen P*, Sinan LOH, Lammy S*, Grivas A, Tilling EJ (Queen Elizabeth University Hospital, United Kingdom); Brown O, Boal M, Dean H, Higgs S*, Stanger S, Abdalaziz H, Constable J, Ishii H, Preece R, Dovell G, Gopi reddy R (Gloucestershire Royal Hospital, United Kingdom); Madhuri TK*, Tailor A, Flavin M, Walker D*, Humphries S, Assalaarachchi H (Royal Surrey NHS Foundation Trust, United Kingdom); Curl-Roper T, Westwood E, Delimpalta C, Liao CCL, Velchuru V* (James Paget University NHS Foundation Trust Hospital, United Kingdom); Raptis DA, Pollok JM*, Machairas N, Davidson B, Fusai G, Soggiu F, Xyda S, Hidalgo Salinas C, Tzerbinis H, Pissanou T, Gilliland J, Chowdhury S, Varcada M*, Hart C, Mirnezami R, Knowles J, Angamuthu N (Royal Free Hospital, United Kingdom); Vijay V*, Shakir T, Hasan R, Tansey R (Princess Alexandra Hospital, Harlow, United Kingdom); Hardie C, Powell-Smith E* (Harrogate District Hospital, United Kingdom); Kashora F*, Siddique MH, Singh A, Barmpagianni C, Basgaran A, Basha A, Okechukwu V, Bartsch A, Gallagher P, Maqsood A, Sahnan K, Leo CA, Lewis SE, Ubhi HK, Exley R, Khan U, Shah P, Saxena S, Zafar N, Abdul-Jabar H (Northwick Park Hospital, United Kingdom); Patel M, Shabana A, Alanbuki A*, Usman O (Brighton and Sussex NHS Trust, Princess Royal Hospital, United Kingdom); Ong CT, Butterworth W*, Budha Magar O, El Hadi M, Abas S, Annett J (Hereford County Hospital, United Kingdom); Ross E, Loubani M*, Wilkins A, Cao H, Capitelli-McMahon H, Hitchman L, Ikram H, Andronic A, Aboelkassem Ibrahim A, Totty J (Hull University Teaching Hospitals NHS Trust, United Kingdom); Blanco J*, Vanker R, Ghobrial M, Jones G, Kanthasamy S, Fawi H, Awadallah M, Chen F, Cheung J* (Hinchingbrooke Hospital, United Kingdom); Moscalu A*, Bhuvanakrishna T, Bibby L, Sinclair M (Ipswich Hospital, United Kingdom); Nahid MAK*, Williams L, Basnyat PS, Shrestha AK* (William Harvey Hospital, United Kingdom); Kumaran NK, Sambhwani S, Sheikh NA, Taylor OM* (Kettering General Hospital, United Kingdom); Liew I*, Al-Sukaini A, Mediratta S, Saxena D (Queen Elizabeth Hospital King's Lynn, United Kingdom); Sgrò A, Rashid MM, Milne K, McIntyre J, Akhtar MA*, Turnbull A, Brunt A, Stewart KE (Victoria Hospital Kirkcaldy, United Kingdom); Wilson MSJ*, Rutherford D, McGivern K, Massie E (Forth Valley Royal Hospital, United Kingdom); Ho M*, Wade RG*, Johnstone J, Bourke G, Brunelli A, Elkadi H, Otify M*, Pompili C, Burke JR, Bagouri E, Chowdhury M, Abual-Rub Z, Kaufmann A, Munot S, Lo T*, Young A, Kowal M, Wall J, Peckham-Cooper A (The Leeds Teaching Hospitals NHS Trust , United Kingdom); Layton GR*, Karki B, Jeong H, Pankhania S, Asher S, Folorunso A, Mistry S, Singh B, Winyard J, Mangwani J (Leicester General Hospital, United Kingdom); Caruana EJ*, Mohammad A, Acharya M, Chandarana K, Ang K, Chowdhry MF, Rathinam S, Nakas A (Glenfield Hospital, United Kingdom); Boddy A*, Hossain T, Ashmore C, Annamalai S, Kourdouli A, Irvine E, Al-Harbawi A, Kassam K*, Al-Harbawee A, Miller A,Mair M (Leicester Royal Infirmary, United Kingdom); Lunevicius R*, Sheel ARG, Sundhu M, Santini AJA, Fathelbab MSAT, Hussein KMA, Nunes QM, Jones RP, Shahzad K, Haq I, Baig MMAS, Hughes JL, Kattakayam A, Rajput K, Misra N, Shah SB, Clynch AL, Georgopoulou N, Sharples HM, Apampa AA, Nzenwa IC, Sud A (Aintree Hospital, Liverpool University Hospitals NHS Foundation Trust, United Kingdom); Harky A, Kirmani BH*, Shackcloth M (Liverpool Heart and Chest Hospital, United Kingdom); Jenkinson MD*, Zakaria R, Elmoslemany T, Millward CP (The Walton Centre NHS Foundation Trust, United Kingdom); Baron R*, Dunne D, Szatmary P, Thomas A, McNicol F, Gahunia S, Sochorova D, Nita GE, McKinney R, Russ J, Tan JR (Royal Liverpool University Hospital, United Kingdom); Harwood R, Corbett H J (Alder Hey In The Park, United Kingdom); Rossborough C, Skelly BL (Altnagelvin Area Hospital, United Kingdom); Che Bakri NA, Nazarian S, Vashisht R*, Jiao L, Jawad Z (BUPA Cromwell Hospital, United Kingdom); Allan AY, Kontovounisios C, Grove T, Warren O*, Fadel MG (Chelsea and Westminster Hospital, United Kingdom); Chatzikonstantinou M, Sorelli P*, Rahman S, Hadjipavlou M (Queen Elizabeth Hospital, Woolwich, United Kingdom); Holbrook C, Chong C, Kufeji D (Evelina London Children's Hospital, London, United Kingdom); Rufai SR, Lloyd IC, James G*, Chari A, Silva AHD (Great Ormond Street Hospital for Children, United Kingdom); Stroman L, Challacombe B*,Sayasneh A*, Najdy M, Billè A, Fraser S, Agoston P, Rizzo V, King J, Nath R, McCrindle Sarah, Mehra G, Harrison-Phipps K, Pilling J, Okiror L, Routledge T, Mills L, Wali A, El-Boghdadly K* (Guy's Hospital, United Kingdom); Fotopoulou C*, Saso S, Fehervari M*, Ploski J, Ghaem-Maghami S, Spalding D, Rajagopal P, Pai M, Habib N, Jawad Z, Hamrang-Yousefi S, Jiao L (Hammersmith Hospital, United Kingdom); Tayeh S*, Chase T, Humphreys L,

For peer review only - http://bmjopen.bmj.com/site/about/guidelines.xhtml

Ayorinde J, Ghanbari A, Cuming T (Homerton University Hospital, United Kingdom); Anscomb N*, Baldwin-Smith R, Rizk M, Grainger C, Davies M, A Surendran, Nunoo-Mensah JW (King's College Hospital, United Kingdom); Dunstan M*, Beak P, Gerogiannis I* (Kingston Hospital NHS Foundation Trust, United Kingdom); Jain A, Menon A, Pramodana B, Choi D*, Marcus HJ, Webber L, May R, Hutchison R, Luoma V (The National Hospital for Neurology and Neurosurgery, United Kingdom); Ranjit S, Parakh J, Sarodaya V, Daadipour A, Khalifa M (Newham University Hospital, United Kingdom); Bosch KD*, Bashkirova V, Dvorkin LS, Kalidindi VK (North Middlesex University Hospital, London, United Kingdom); Dudek J, Singhal T*, El-Hasani S (Princess Royal University Hospital, United Kingdom); De Souza A, Cannoletta M, Rochon M, Bhudia S (Royal Bromtpon Hospital, London, United Kingdom); Bennett S, Navaratne L, Venn M, Yip V*, Kayani B, Sohrabi C, Kocher HM, Minicozzi A, Banerjee A, Sullivan T, Sivaprakasam R*, Anzak A, Ghufoor K*, Thaha MA*, Knowles C, Ledesma FS, Patki P*, Popova D, Sadigh P*, Ramamoorthy R, Uff C*, Attwell L, Tanabalan C, Goh MA, Jayasinghe JD, Leal Silva I, Thakur B, Lebe M, Thet MS, Hughes F*, Rahman R, Fuwa O (Royal London Hospital, United Kingdom); Sanders J*, Oo A, Bueser T, Curtis M, Stamenkovic SA (Cardiac Unit, St Bartholomew's Hospital, United Kingdom); Abbott T*, Anwar S, Sohrabi C (St Bartholomew's Hospital, United Kingdom); Williams K*, Chung E, Hagger R, Karim A, Hainsworth A, Flatman M, Trompeter A, Hing C, Brown O, Tsinaslanidis P, Benjamin MW, Leyte A, Tan C, Smelt J, Vaughan P, Santhirakumaran G, Hunt I, Raza M, A Labib (St George's Hospital, United Kingdom); Luo X, Sudarsanam A, Rolls A, Lyons O, Onida S, Shalhoub J, Sugand K, Park C, Sarraf KM*, Erridge S, Kinross J*, Denning M, Yalamanchili S, Abuown A, Ibrahim M, Martin G (St Mary's Hospital, United Kingdom); Davenport D, Wheatstone S* (St Thomas' Hospital, United Kingdom); Kasivisvanathan V*, Kapriniotis K, Elhamshary A, Imam SMB (University College London Hospital At Westmoreland Street, United Kingdom); Kalavrezos N*, Sinha D, Chand M Green L, Beech N, McEwen R, Kiconco H (University College London Hospital, United Kingdom); Andreani SM*, Bath MF, Sahni A, Judkins N, Rigueros Springford L, Sohrabi C, Bacarese-Hamilton J, Taylor FG, Patki P*, Tanabalan C (Whipps Cross University Hospital, United Kingdom); Parmar C*, Mccluney S, Shah S (The Whittington Hospital, United Kingdom); Talwar R*, Patel K, Askari A, Jambulingam P S, Shaw S, Maity A, Hatzantonis C, Sagar J, Kudchadkar S, Cirocchi N, Chan CH (Luton and Dunstable University Hospital, United Kingdom); Reynolds J, Alexander ME, Smart CJ* (Macclesfield District General Hospital, United Kingdom); Jayasankar B, Balasubramaniam D*, Abdelsaid K, Mundkur N, Gallagher B* (Tunbridge Wells Hospital, United Kingdom); Shah J*, Anthoney J, Emmerson O (North Manchester General Hospital, United Kingdom); Stylianides N*, Abdalla M, Newton K, Bhatia K*, Edmondson R, Abdeh L, Jones D, Zeiton M, Ismail O, Naseem H, Advani R (Manchester Royal Infirmary, United Kingdom); Duff S*, Moura F, Brown BC, Khan A, Asaad P, Wadham B, Aneke IA, Collis J, Warburton H (Wythenshawe Hospital, Manchester University NHS Foundation Trust, United Kingdom); Fell A*, Smith A, Halkias C, Evans J, Nikolaou S, English C*, Kristinsson S, Oni T, Ilahi N, Ballantyne K, Woodward Z, Merh R (Queen Elizabeth The Queen Mother Hospital Margate, United Kingdom); Dunning J, Viswanath Y, Freystaetter K, Dixon J*, Hadfield JN, Hilley A, Egglestone A, Smith B (James Cook University Hospital, United Kingdom); Hine T, Keeler B*, Soulsby RE, Taylor A* (Milton Keynes University Hospital, United Kingdom); Davies E*, Ryska O, Raymond T, Rogers S*, Tong A, Hawkin P (Royal Lancaster Infirmary, United Kingdom); Tingle S, Abbadessa F, Sachdeva A, Rai B*, Chan CD, McPherson I, Booth K, Mahmoud Ali F, Pandanaboyana S, Grainger T, Nandhra S, Patience A, Rogers A*, Roy C, Williams T, Dawe N, McCaffer C, Riches J, Bhattacharya S, Moir J, Kalson NS, Elamin Ahmed H, Mellor C, Saleh C, Koshy RM, Hammond J*, Sanderson L, Wahed S, Phillips AW, Ghosh K* (Newcastle Upon Tyne Hospitals NHS Foundation Trust, United Kingdom); Tang A, Beamish AJ, Price C, Bosanquet D*, Magowan D, Solari F, Williams G, Nassa H, Smith L (Royal Gwent Hospital, United Kingdom); Robertson-Smith B, Mahmoud A, Ameerally P, Finch JG*, Gnanachandran C, Pop I, Rogers M, Yousef Y, Mohamed I, Woods R, Zahid H, Mundy G* (Northampton General Hospital, United Kingdom); Youssef M*, Sreedharan L, Baskaran D, Shaikh I, Seebah K, Reid J, Watts D, Kouritas V, Chrastek D, Maryan G, Gill DF, Khatun F (Norfolk and Norwich University Hospital, United Kingdom); Gajjar K, Williamson K, Bratt D, Konstantinidi K, Walton T*, Burnside N*, Weaver H, Hawari M, Addae-Boateng E, Rollett RA*, Collins ML, Tamimy MS, Riyat H, Wen J, Neil-Dwyer J (Nottingham City Hospital, United Kingdom); Brewer H, Humes D*, Worku D, Chowdhury A*, Oyende O, Lewis-Lloyd C, Adiamah A, Koh A, Jackman J, Vohra R*, Navarro A*, Reilly J (Queens Medical Centre, United Kingdom); Aujayeb A, Townshend D*, McLarty N, Shenfine A, Jackson K, Johnson C (Northumbria NHS Hospital Trust, United Kingdom); Dass D, Ford D (Robert Jones and Agnes Hunt Orthopaedic Hospital, United Kingdom); Winter SC*, Belcher E*, Stavroulias D, Di Chiara F, Wallwork K, Qureishi A, Lami M, Sravanam S, Mastoridis S, Shah K, Chidambaram S, Smillie R, Shaw AV, Bandyopadhyay S, Cernei C, Bretherton C, Jeyaretna D*, Ganau M*, Piper RJ*, Duck E, Brown S, Jelley C, Tucker SC*, Bond-Smith G*, Griffin XL*, Tebala GD, Neal N*, Vatish M*, Noton TM, Ghattaura H, Maher M, Fu H, Risk OBF, Soleymani majd H, Sinha S*, Shankar S, Aggarwal A, Kharkar H, Lakhoo K*, Verberne C, Mastoridis S

(Oxford University NHS Foundation Trust, United Kingdom); Dean B*, Luney C, Myatt R, Williams MA, McVeigh J (Nuffield Orthopaedic Centre, United Kingdom); Rogers LJ*, Labib PL, Miller D, Minto G, Hope N, Marchbank A, Emslie K, Panahi P, Ho B, Perkins C, Clough E, Roy H, Enemosah I, Campbell R, Natale J, Gohil K, Rela M, Raza N, (Derriford Hospital, United Kingdom); Biliatis I* (Poole Hospital, United Kingdom); Khan J*, Thiruchandran G, Toh SKC*, Ahmad Y*, Allana A, Bellis C, Babawale O, Phan YC, Lokman U, Ismail M, Koc T, Witek A, Duggleby L, Shamoon S, Stefan S, Clancy H (Queen Alexandra Hospital, United Kingdom); Chadha R*, Middleton SB, Wilmott K, Hayden C, Mclaren C, Sutton J, Whyte A (Royal Berkshire Hospital, United Kingdom); Belgaumkar A*, Day A, Gilbert C, Oyewole B, Narayan P, Dent H, Sandhya A, De Silva T, Waheed S (Surrey and Sussex Healthcare NHS Trust, United Kingdom); Day A, Kapoor K, Belgaumkar AP*, Narayan P, Fahim M, (East Surrey Hospital, United Kingdom); Gala T, Mithany R, Morgan R*, Abdelkarim M, Ibrahim S, Maw A*, Asqalan A, Sundaram Venkatesan G (Glan Clwyd Hospital, United Kingdom); Singh S, Mukherjee S*, Ferguson D, Smith C, Mansuri A, Thakrar A, Wickramarachchi L, Cuthbert R, Sivayoganathan S, Chui K, Karam E, Dott C, Shankar S (Queen's Hospital Romford, United Kingdom); Madhvani K, Hampton M, Hormis AP* (Rotherham District General Hospital, United Kingdom); Thomas M, Pearce L*, Fountain DM, Laurente R, Sigamoney KV, Dasa M, George K*, Naqui Z*, Galhoum M, Lipede C, Gabr A, Radhakrishnan A, Hasan MT, Kalenderov R, Pathmanaban O, Colombo F, Chelva R (Salford Royal NHS Foundation Trust, United Kingdom); Branagan G*, Longstaff L, Ding D, Barlow C, Foster J (Salisbury NHS Foundation Trust, United Kingdom); Edwards J*, Ward A, Tadross D, Majkowski L, Blundell C, Forlani S, Nair R, Guha S, Brown SR, Steele C, Kelty CJ*, Newman T, Lee M, Chetty G*, Lye G, Balasubramanian SP, Sureshkumar Shah N, Sherif M, Al-mukhtar A, Whitehall E, Giblin A, Wells F, Sharkey A, Adamec A, Madan S (Sheffield Teaching Hospital NHS Foundation Trust, United Kingdom); Narice B, Sterrenburg M, Thompson A, Varley I, Stavrakas M, Rominiyi O, Ray J, Adamec A, Crank M, Bacon A, Al-Tamimi Y, Catto J, Saad S, Abd Kahar NN, Sinha S (Royal Hallamshire Hospital, United Kingdom); Sou A, Simpson D, Hamilton E*, Blair J (Shrewsbury and Telford Hospitals, United Kingdom); Jallad S, Lord J, Anderson C, El Kafsi J*, Logishetty K*, Saadya A, Midha R, Ip M, Subbiah Ponniah H, Stockdale T, Bacarese-Hamilton T, Foster L, James A, Anjarwalla N, Marujo Henriques D, Hettige R, Baban C, Tenovici A, Salerno G (Wexham Park, Frimley Health NHS FT, United Kingdom); Singh R*, Lane J, Colvin HV, Badran A*, Cadersa A, Williams S, Cumpstey A, Hamady Z, Aftab R, Wensley F, Byrne J, Morrison-Jones V, Sekhon GK, Shields H, Shakoor Z, Yener A, Talbot T, Khan A, Alzetani A*, Cresner R (Southampton General Hospital, United Kingdom); Babu BHB*, Liyanage ASD, Newman S, Blake I, Weerasinghe C (Southport and Formby District General Hospital, United Kingdom); Baumber R, Parry J* (Royal National Orthopaedic Hospital, United Kingdom); Menakaya C*, Webb JI, Antar M, Modi N, Sofat R, Noel J, Nunn R, Adegbola S, Eriberto F, Sharma V, Tanna R, Lodhia S (East and North Hertfordshire NHS Trust, Lister Hospital, United Kingdom); Johnson D*, Hughes I, Hall J, Rooney J, Chatterji S, Zhang Y, Owen R, Rudic M, Hunt J (Stepping Hill Hospital, United Kingdom); Zakai D, Thomas M*, Aladeojebi A, Ali M, Gaunt A*, Barmayehvar B, Kitchen M, Gowda M, Mansour F, Jarvis M, Halliday E, Lefroy R, Nanjaiah P, Ali S, Kitchen M* (Royal Stoke University Hospital, United Kingdom); Lin DJ, Rajgor AD, Scurrah RJ, Kang C, Watson LJ, Harris G, Royle T*, Cunningham Y, James G, Steel B, Luk ACO (Sunderland Royal Hospital, United Kingdom); Boulton AJ, Khan T, Bakolas G, Ahmed I* (Good Hope Hospital, United Kingdom); Herrod P, Gemmill E*, Boyd-Carson H, Jibreel M, Lenzi E, Saafan T, Sapre D (King's Mill Hospital, United Kingdom); Li Z*, Parkins K, Spencer N, Harries R, Egan RJ, Motter D, Jenvey C, Mahoney R, Fine N, Minto T, Henry A (Morriston Hospital Swansea, United Kingdom); Hollyman M*, Grieco C, Gemmell C (Musgrove Park Hospital, United Kingdom); Whitmore H, Babar MS, Goodrum S, Scott R, Collard B, Lau K, Thomas E, Patel A, Allison J, Bowen J (Torbay and South Devon NHS Trust, United Kingdom); Dias A, Mahendran B, Gopalswamy S* (Royal Cornwall Hospital, Truro, United Kingdom); Patil S, Scott L, Sarveswaran J*, Michel M, Ravindran S (Pinderfields Hospital, United Kingdom); Subba K, Abou-Foul AK, Khalefa M, Hossain F*, Moores T, Pickering L (Walsall Manor Hospital NHS Trust, United Kingdom); Stables G*, Doorgakant A*, Thiruvasagam VG, Carter J, Reid S, Mohammed R, Marlow W (Warrington & Halton Teaching Hospitals NHS Trust, United Kingdom); Ferguson H*, Wilkin R, Konstantinou C, Yershov D, Vatish J, Denning A (South Warwickshire NHS Foundation Trust, United Kingdom); Shah HB, Cross GWV, Seyed-Safi P*, Smart YW, Kuc A, Al-Yaseen M (Watford General Hospital, United Kingdom); Olivier J*, Hanna M, Eskander P, Duncan R, Halaseh S (Weston General Hospital, United Kingdom); Das R* (Hampshire Hospitals NHS Trust, United Kingdom); Wynn Jones H, Divecha H*, Whelton C, Board T (Wrightington, Wigan & Leigh NHS Foundation Trust, United Kingdom); Powell S*, Magee C*, Agarwal K*, Mangos E, Nambirajan T (Wirral University Teaching Hospital, United Kingdom); Vidya R*, Chauhan G, Kaur J, Burahee A, Bleibleh S, Pigadas N*, Snee D, Bhasin S*, Crichton A, Habeebullah A, Bodla AS, Yassin N*, Mondragon M, Dewan V (Royal Wolverhampton NHS Trust, United Kingdom); Flindall I, Mahendran V, Hanson A (Worcestershire Royal Hospital, United Kingdom); Jenner E, Richards J,Thomas-Fernandez K, Wall R (Worcestershire Acute

Hospitals NHS Trust, United Kingdom); Alqallaf A, Ben-Sassi A*, Mohamed I, Mellor K, Joshi P, Joshi Y (Wrexham Maelor Hospital, United Kingdom); Young R*, Miu V, Sheridan K, MacDonald L, Green S, Onos L (York Teaching Hospitals NHS Trust, United Kingdom); Wong JJ, Napolitano L*, Hemmila M (Michigan Medicine, United States); Amin D*, Abramowicz S, Roser SM (Emory University, United States); Olson KA, Riley C, Heron C, Cardenas T*, Leede E, Thornhill M, Haynes AB*, McElhinney K, Roward S, Trust MD*, Hill CE, Teixeira PG* (Dell Seton Medical Center at the University of Texas at Austin, United States); Etchill E, Stevens K*, Ladd MR, Long C, Rose J, Kent A, Yesantharao P, Vervoort D, Jenny H, Gabre-Kidan A, Margalit A, Tsai L, Malapati H, Yesantharao L (Johns Hopkins Hospital, United States); Abdou H, Diaz J*, Richmond M, Clark J, O'Meara L, Hanna N (University of Maryland Medical Center, United States); Ying Y*, Fleming J, Ovaitt A, Gigliotti J, Fuson A (University of Alabama Birmingham, United States); Cooper Z*, Salim A*, Hirji SA, Brown A, Chung C, Hansen L, Okafor BU, Roxo V, Raut CP, Jolissaint JS, Mahvi DA (Brigham and Women's Hospital, United States); Kaafarani H*, Breen K, Bankhead-Kendall B, Alser O, Mashbari H, Velmahos G, Maurer LR, El Moheb M, Gaitanidis A, Naar L, Christensen MA, Kapoen C, Langeveld K, El Hechi M, Mokhtari A (Massachusetts General Hospital, United States); Haqqani MH, Drake FT* (Boston Medical Center, United States); Goldenberg-Sandau A*, Galbreath B (Cooper University Hospital, United States); Reinke C*, Ross S, Thompson K, Manning D, Perkins, R (Atrium Health Carolinas Medical Center, United States); Eriksson E*, Evans H (Medical University of South Carolina, United States); Masrur M, Giulianotti P, Benedetti E (University of Illinois at Chicago, Chicago, IL, United States); Chang G*, Ourieff J, Dehart D (Mount Sinai Hospital, United States); Dorafshar A, Price T, Bhama AR, Torquati A*, Cherullo E, Kennedy R, Myers J (Rush University Medical Centre, United States); Rubin K* (University Hospitals of Cleveland, United States); Ban VS*, Aoun SG, Batjer HH, Caruso J (University of Texas Southwestern, United States); Carmichael H, Velopulos CG*, Wright FL, Urban S, McIntyre Jr RC, Schroeppel TJ*, Hennessy EA, Dunn J*, Zier L (University of Colorado Hospital/Memorial Hospital/Medical Center of the Rockies, United States); Burlew C*, Coleman J* (Denver Health, United States); Colling KP* (Saint Mary's Medical Center-Essentia Health, United States); Hall B, Rice HE*, Hwang ES, Olson SA, Moris D (Duke University Medical Center, United States); Verma R*, Hassan R (Nassau University Medical Center, United States); Volpe A, Merola, S (NewYork Presbyterian Queens, Flushing, United States); O'Banion LA*, Lilienstein J, Dirks R (University of California San Francisco-Fresno, United States); Marwan H*, Almasri M*, Kulkarni G, Mehdi M, Abouassi A, Abdallah M, San Andrés M, Eid J, Aigbivbalu E, Sundaresan J, George B (University of Texas Medical Branch, United States); Ssentongo A, Ssentongo P, Oh JS, Hazelton J*, Maines J, Gusani N, Garner M, Horvath S (Pennsylvania State University, United States); Zheng F* (Houston Methodist Hospital, United States); Ujiki M (Northshore University Healthsystem, United States); Kinnaman G, Meagher A*, Sharma I, Holler E (Iu Health Methodist Hospital, United States); McKenzie K*, Chan J, Fretwell K, Nugent III W, Khalil A, Chen D, Post N, Rostkowski T, Brahmbhatt D (Jamaica Hospital, United States); Huynh K, Hibbard ML (Kaiser Permanente West Los Angeles, United States); Schellenberg M* (Lac+Usc Medical Center, United States); Martin RCG*, Bhutiani N (University of Louisville Hospital and Norton Hospital, Louisville, United States); Giorgakis E*, Laryea J, Bhavaraju A, Sexton K, Roberts M, Kost M, Kimbrough M, Burdine L, Kalkwarf K, Robertson R (University of Arkansas for Medical Sciences, United States); Gosain A*, Camp L, Lewit R (Le Bonheur Children's Hospital, United States); Kronenfeld JP, Urrechaga E, Goel N, Rattan R, Hart V*, Allen M, Gilna G (Jackson Memorial Hospital, United States); Cioci A, Ruiz G*, Allen M, Rakoczy K, Pavlis W, Saberi R (University of Miami Hospital, United States); Morris R*, Karam BS (Froedtert Hospital, United States); Brathwaite CEM*, Liu H, Petrone P, Hakmi H, Sohail AH, Baltazar G, Heckburn R (NYU Langone Health-NYU Winthrop Hospital, United States); Nygaard RM*, Colonna ET, Endorf FW, Hill MJ (Hennepin Healthcare, Minneapolis, United States); Maiga A, Dennis B*, Levin JH, Lallemand M (Vanderbilt University Medical Center, United States); Choron R, Peck G*, Soliman F, Rehman S (Robert Wood Johnson University Hospital, United States); Glass N*, Juthani B, Deisher D (The University Hospital, United States); Ruzgar NM, Ullrich SJ*, Sion M* (Yale New Haven Hospital, United States); Paranjape C*, El Moheb M, Kar AR (Newton Wellesley Hospital, United States); Gillezeau C, Rapp J, Taioli E, Miles BA*, Alpert N (Mount Sinai Hospital, United States); Podolsky D*, Coleman NL, Callahan MP (NewYork-Presbyterian / Columbia University Medical Center, United States); Ganly I*, Brown L (Memorial Sloan Kettering Cancer Center, United States); Monson JRT (AdventHealth Orlando, Orlando, United States); Dehal A* (Kaiser Permanente Panorama City Medical Center, United States); Abbas A, Soliman A, Kim B, Jones C, Dauer, MD E, Renza-Stingone E, Hernandez E, Gokcen E, Kropf E, Sufrin H, Hirsch H, Ross H, Engel J, Sewards J, Diaz J, Poggio J, Sanserino K, Rae L, Philp M*, Metro M, McNelis P, Petrov R, Rehman S, Pazionis T (Temple University Hospital, United States); Till B, Lamm R, Rios-Diaz AJ, Palazzo F* (Thomas Jefferson University Hospital, United States); Rosengart M*, Nicholson K (University of Pittsburgh Medical Center, United States); Carrick MM*, Rodkey K (Medical City Plano, United States); Suri A, Callcut R* (UC Davis Medical Center, United

States); Nicholson S*, Talathoti N (UT Health San Antonio (UTHSA), University Hospital (UHS), San Antonio, United States); Klaristenfeld D* (Kaiser Permanente San Diego Medical Center, United States); Biffl W*, Marsh C, Schaffer K (Scripps Memorial Hospital, United States); Berndtson AE*, Averbach S, Curry T (University of California San Diego, United States); Kwan-Feinberg R*, Consorti E, Gonzalez R, Grolman R, Liu T, Merzlikin O (Alta Bates Summit Medical Center (Sutter Health), United States); Abel MK, Ozgediz D, Boeck M, Kornblith LZ*, Nunez-Garcia B, Robinson B, Park P (University of California San Francisco (UCSF), United States); Utria AF, Rice-Townsend SE*, Javid P, Hauptman J, Kieran K (Seattle Children's Hospital, United States); Nehra D*, Walters A, Cuschieri J, Davidson GH (Harborview Medical Center, United States); Nunez J*, Cosker R (University of Utah Healthcare, Salt Lake City, United States); Eckhouse S* (Washington University School of Medicine, United States); Choudhry A, Marx W* (Suny Upstate University Hospital, United States); Jamil T*, Seegert S, Al-Embideen S (ProMedica Toledo Hospital, United States); Quintana M*, Jackson H (The George Washington University Hospital, United States); Wexner SD, Kent I (Cleveland Clinic Florida, Weston, United States); Martins PN* (University of Massachusetts, UMass Memorial Hospital, United States); Alshehari M*, Al-Naggar H*, Alsayadi M, Alyazidi M, Shream S, Alhaddad W, Maqus A, Abu Hamra'a M,Alsayadi R, Ghannam R, Al-Maqtari S, Masdoos S, Al-Harazi Y, Bajjah H (Al-Thawra Modern General Hospital, Yemen); Al-ameri S, Aldawbali M (Royal Hospital, Yemen); Gwini GP* (Mpilo Central Hospital, Bulawayo, Zimbabwe); Mazingi D* (Parirenyatwa hospital, Harare, Zimbabwe)

Acknowledgements: Xiao Liu, Alistair Denniston, Royal College of Surgeons of England COVID-19 Research Group

For peer review only

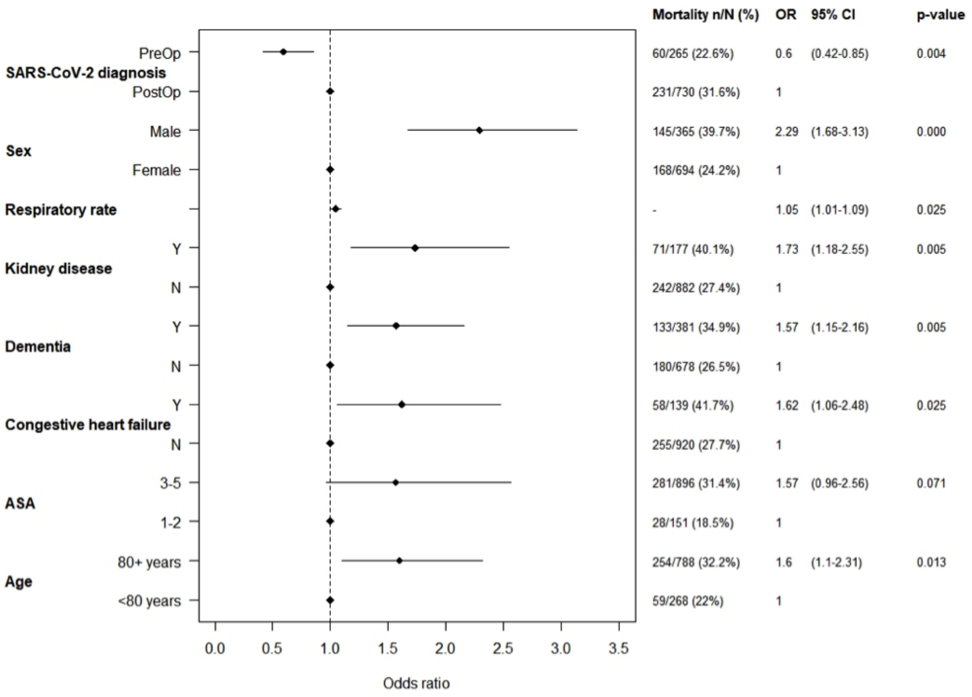

Fig. 1 Mixed-effects logistic regression model for 30 day mortality

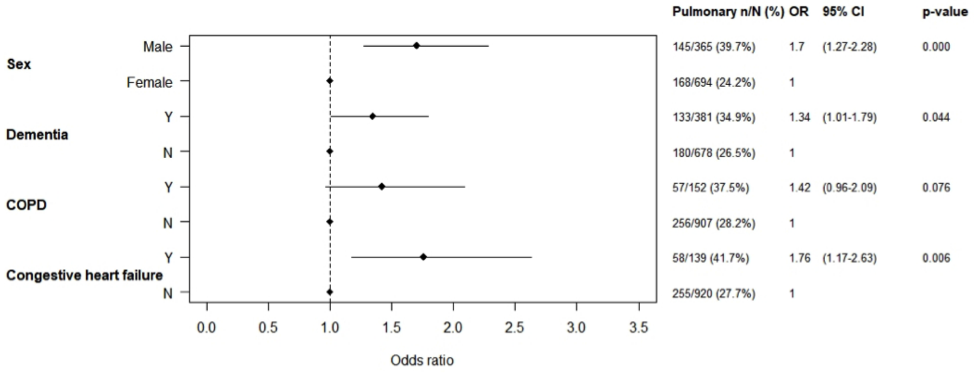

Fig. 2. Mixed-effects logistic regression model for pulmonary complications

Supplementary Material

Table S1. Baseline characteristics (sex, age, ASA grade, cardiac risk, time of diagnosis, method of diagnosis and month study participant recruited) of the full study population (n = 1063), and died (n = 313) and alive (n =746) groups; p-values are for Fisher's exact tests comparing groups for each characteristic.

Characteristic	Full (n = 1063)	Alive (n = 746)	Died (n = 313)	Died (%)
*Sex (p-value = <0.001 ***)*				
Female	696 (65.5%)	526	168	24.2
Male	367 (34.5%)	220	145	39.7
Missing	0 (0.0%)	0	0	0.0
*Age (p-value = 0.001 **)*				
20-29 years	3 (0.3%)	3	0	0.0
30-39 years	2 (0.2%)	1	1	50.0
40-49 years	4 (0.4%)	3	1	25.0
50-59 years	24 (2.3%)	21	3	12.5
60-69 years	50 (4.7%)	39	11	22.0
70-79 years	189 (17.8%)	145	43	22.9
80-89 years	507 (47.7%)	360	144	28.6
90+ years	284 (26.7%)	174	110	38.7
Missing	0(0.0%)	0	0	0.0
*ASA (p-value = 0.001 **)*				
1-2	151 (14.2%)	123	28	18.5
3-5	899 (84.6%)	615	281	31.4
Missing	13(1.2%)	8	4	30.8

For peer review only

*Cardiac risk (p-value = <0.001 ***)*				
0	487 (45.8%)	372	114	23.5
1	349 (32.8%)	238	110	31.6
2	169 (15.9%)	106	61	36.5
3	44 (4.1%)	23	21	47.7
4	8 (0.8%)	5	3	37.5
5	1 (0.1%)	0	1	100.0
Missing	5 (0.47%)	2	3	60.0
*Time of diagnosis (p-value = 0.006 **)*				
Post-op	733 (69%)	499	231	31.6
Pre-op	266 (25%)	205	60	22.6
Missing	64 (6.0%)	42	22	34.4
Diagnosis (p-value = 0.668)				
Clinical	62 (5.8%)	42	20	32.3
Swab	992 (93.3%)	696	292	29.6
Missing				
*Month (p-value = 0.007 **)*				
February	26 (2.4%)	23	3	11.5
March	474 (44.6%)	313	159	33.7
April	558 (52.5%)	406	150	27.0
Missing	1(0.09%)	0	1	100.0

For peer review only

Table S2. Comorbidity data summaries by died (n = 313) and alive (n =746) groups. Data tabulated are counts, with estimated odds ratios (OR), with 95% confidence intervals, and p-values from Fisher's exact tests for each comorbidity.

Comorbidity	Alive	Died	OR	p-value

	(n = 746)	(n = 313)	(95% CI)		
	Y:N (%Y)	Y:N (%Y)			
Current smoker	34:712 (4.6%)	9:304 (2.9%)	0.62 (0.26, 1.34)	0.235	-
Asthma	53:693 (7.1%)	21:292 (6.7%)	0.94 (0.53, 1.62)	0.895	-
Current cancer diagnosis	57:689 (7.6%)	24:289 (7.7%)	1.00 (0.58, 1.68)	0.999	-
Chronic kidney disease (moderate/severe)	106:640 (14.2%)	71:242 (22.7%)	1.77 (1.25, 2.51)	0.001	**
Chronic obstructive pulmonary disease (COPD)	95:651 (12.7%)	57:256 (18.2%)	1.53 (1.05, 2.21)	0.027	*
Congenital abnormality - cardiac	4:742 (0.5%)	1:312 (0.3%)	0.60 (0.01, 6.04)	0.999	-
Congenital abnormality - non-cardiac	0:746 (0.0%)	4:309 (1.3%)	-	-	-
Congestive heart failure	81:665 (10.9%)	58:255 (18.5%)	1.87 (1.27, 2.73)	<0.001	***
Dementia	248:498 (33.2%)	133:180 (42.5%)	1.48 (1.12, 1.96)	0.005	**
Diabetes mellitus	142:604 (19.0%)	63:250 (20.1%)	1.07 (0.76, 1.51)	0.671	-
Hypertension	387:359 (51.9%)	186:127 (59.4%)	1.36 (1.03, 1.79)	0.026	*
Myocardial infarction or ischemic heart disease	103:643 (13.8%)	63:250 (20.1%)	1.57 (1.09, 2.25)	0.012	*
Peripheral vascular disease	34:712 (4.6%)	21:292 (6.7%)	1.51 (0.82, 2.72)	0.172	-
Stroke/ TIA	107:639 (14.3%)	57:256 (18.2%)	1.33 (0.92, 1.92)	0.114	-
Other (including other lung disease)	377:369 (50.5%)	158:155 (50.5%)	1.00 (0.76, 1.31)	0.999	-

Table S3. Diagnosis data summaries by died (n = 313) and alive (n =746) groups. Data tabulated are counts, with estimated odds ratios (OR), with 95% confidence intervals, and p-values from Fisher's exact tests for each diagnosis method.

Diagnosis	Alive (n = 746)	Died (n = 313)	OR (95% CI)	p-value	
	Y:N (%Y)	Y:N (%Y)			
Pre-op 4-7days					
CT thorax scan (negative for SARS-CoV-2)	10:736 (1.3%)	2:311 (0.6%)	0.47 (0.05, 2.24)	0.526	-

For peer review only

CT thorax scan (positive for SARS-CoV-2)	12:734 (1.6%)	0:313 (0%)	0.00 (0.00, 0.85)	0.023	*
Swab (negative for SARS-CoV-2)	17:729 (2.3%)	3:310 (1%)	0.42 (0.08, 1.45)	0.215	-
Swab (positive for SARS-CoV-2)	31:715 (4.2%)	8:305 (2.6%)	0.61 (0.24, 1.37)	0.283	-
Pre-op 1-3days					
CT thorax scan (negative for SARS-CoV-2)	8:738 (1.1%)	3:310 (1%)	0.89 (0.15, 3.75)	0.999	-
CT thorax scan (positive for SARS-CoV-2)	10:736 (1.3%)	3:310 (1%)	0.71 (0.13, 2.79)	0.765	-
Swab (negative for SARS-CoV-2)	41:705 (5.5%)	9:304 (2.9%)	0.51 (0.22, 1.08)	0.080	-
Swab (positive for SARS-CoV-2)	86:660 (11.5%)	15:298 (4.8%)	0.39 (0.20, 0.69)	<0.001	*
Pre-op surgery					
CT thorax scan (negative for SARS-CoV-2)	0:746 (0%)	0:313 (0%)	-	0.999	-
CT thorax scan (positive for SARS-CoV-2)	4:742 (0.5%)	0:313 (0%)	0.00 (0.00, 3.61)	0.326	-
Swab (negative for SARS-CoV-2)	13:733 (1.7%)	2:311 (0.6%)	0.36 (0.04, 1.62)	0.254	-
Swab (positive for SARS-CoV-2)	18:728 (2.4%)	7:306 (2.2%)	0.93 (0.32, 2.35)	1	-
Post-op Admission					
CT thorax scan (negative for SARS-CoV-2)	4:742 (0.5%)	1:312 (0.3%)	0.60 (0.01, 6.04)	0.999	-
CT thorax scan (positive for SARS-CoV-2)	8:738 (1.1%)	4:309 (1.3%)	1.19 (0.26, 4.50)	0.756	-
Swab (negative for SARS-CoV-2)	51:695 (6.8%)	12:301 (3.8%)	0.54 (0.26, 1.05)	0.064	-
Swab (positive for SARS-CoV-2)	317:429 (42.5%)	143:170 (45.7%)	1.14 (0.87, 1.50)	0.342	-
Discharge 30days					
CT thorax scan (negative for SARS-CoV-2)	4:742 (0.5%)	0:313 (0%)	0.00 (0.00, 3.61)	0.326	-
CT thorax scan (positive for SARS-CoV-2)	0:746 (0%)	1:312 (0.3%)	-	-	-
Swab (negative for SARS-CoV-2)	8:738 (1.1%)	0:313 (0%)	0.00 (0.00, 1.39)	0.114	-
Swab (positive for SARS-CoV-2)	75:671 (10.1%)	27:286 (8.6%)	0.85 (0.51, 1.36)	0.496	-
Diagnosis					
Positive SARS-CoV-2 swab - before surgery	122:624 (16.4%)	31:282 (9.9%)	0.56 (0.36, 0.86)	0.007	*
Positive SARS-CoV-2 swab - after surgery	409:337 (54.8%)	177:136 (56.5%)	1.07 (0.82, 1.41)	0.636	-

For peer review only

CT scan of the chest confirming SARS-CoV-2 - before surgery	20:726 (2.7%)	2:311 (0.6%)	0.23 (0.03, 0.97)	0.033	*
CT scan of the chest confirming SARS-CoV-2 - after surgery	9:737 (1.2%)	5:308 (1.6%)	1.33 (0.35, 4.46)	0.569	-
Clinical diagnosis or chest x-ray - suspected before time of surgery	39:707 (5.2%)	12:301 (3.8%)	0.72 (0.34, 1.43)	0.432	-
Clinical diagnosis or chest x-ray - suspected after time of surgery	67:679 (9%)	31:282 (9.9%)	1.11 (0.69, 1.77)	0.643	-

Table S4. SARS-CoV-2 symptoms data summaries by died (n = 313) and alive (n =746) groups. Data tabulated are counts, with estimated odds ratios (OR), with 95% confidence intervals, and p-values from Fisher's exact tests for each symptom.

Symptom	Alive<br>(n = 746)	Died<br>(n = 313)	OR<br>(95% CI)	p-value
	Y:N (%Y)	Y:N (%Y)		
Abdominal pain	11:735 (1.5%)	1:312 (0.3%)	0.21 (0.01, 1.49)	0.124 -
Breathlessness (dyspnoea)	54:692 (7.2%)	31:282 (9.9%)	1.41 (0.86, 2.28)	0.172 -
Cough	73:673 (9.8%)	35:278 (11.2%)	1.16 (0.73, 1.81)	0.505 -
Diarrhoea	8:738 (1.1%)	1:312 (0.3%)	0.30 (0.01, 2.22)	0.295 -
Fatigue	21:725 (2.8%)	10:303 (3.2%)	1.14 (0.47, 2.56)	0.695 -
Fever (>38 celsius)	61:685 (8.2%)	25:288 (8%)	0.98 (0.57, 1.61)	0.999 -
Haemoptysis	0:746 (0.0%)	0:313 (0.0%)	-	- -
Myalgia	10:736 (1.3%)	3:310 (1%)	0.71 (0.13, 2.79)	0.765 -
Nausea/vomiting	13:733 (1.7%)	7:306 (2.2%)	1.29 (0.43, 3.52)	0.623 -
Sputum	8:738 (1.1%)	4:309 (1.3%)	1.19 (0.26, 4.50)	0.756 -
Other	311:435 (41.7%)	136:177 (43.5%)	1.08 (0.82, 1.42)	0.633 -

Table S5. Pre-surgery measures data (n, mean and sd) for the full study population (n = 1063), and died (n = 313) and alive (n =746) groups, and the difference in means between groups, with 95% confidence interval, and p-values from unpaired t-tests.

For peer review only

Measure	Full (n = 1063)		Alive (n = 746)		Died (n = 313)		Difference (95%CI)	p-valu
	n	Mean (SD)	n	Mean (SD)	n	Mean (SD)		
Respiratory rate (breaths/minute)	996	17.75 (3.52)	706	17.65 (3.68)	289	18.02 (3.09)	-0.37 (-0.85, 0.11)	0.132
Heart rate (bpm)	1022	81.10 (14.63)	723	81.16 (14.59)	298	80.95 (14.77)	0.22 (-1.76, 2.20)	0.830
Systolic blood pressure (mmHg)	1023	138.15 (26.04)	724	138.33 (25.85)	298	137.61 (26.53)	0.72 (-2.80, 4.24)	0.687
Diastolic blood pressure (mmHg)	1021	72.97 (13.89)	723	73.07 (13.86)	297	72.70 (14.00)	0.37 (-1.52, 2.25)	0.703
Haemoglobin (g/L)	1062	117.94 (19.22)	745	118.35 (19.21)	313	116.93 (19.26)	1.41 (-1.13, 3.95)	0.276
White cell count (10^9/L)	1060	10.33 (4.26)	744	10.33 (4.36)	313	10.34 (4.04)	-0.01 (-0.57, 0.56)	0.976
C-reactive protein (mg/L)	738	54.70 (66.26)	514	54.84 (67.68)	221	54.63 (63.34)	0.21 (-10.28, 10.70)	0.969

Table S6. Operation details for the full study population (n = 1063), and died (n = 313) and alive (n =746) groups; p-values are for Fisher's exact tests comparing groups for each characteristic.

Characteristic	Full (n = 1063)	Alive (n = 746)	Died (n = 313)	Died (%)
Anaesthesia (p-value = 0.787)				
General	527 (49.6%)	368	157	29.9
Regional	524 (49.3%)	372	152	29.0
Missing	12 (27.8%)	6	4	33.3
*Pre-op respiration (p-value = 0.031 *)*				
None	714 (67.2%)	520	194	27.2
Oxygen	336 (31.6%)	220	115	34.3
Ventilated	2 (0.2%)	2	0	0.0
Missing	11(1.03%)	4	4	36.4
Pre-op delay (p-value = 0.220)				

For peer review only

< 6 hours	16 (1.5%)	11	5	31.2
6-23 hours	322 (30.3%)	232	88	27.5
24-47 hours	224 (21.1%)	147	77	34.4
48-71 hours	82 (7.7%)	61	21	25.6
72+ hours	123 (11.6%)	94	29	23.6
Missing	296 (27.8%)	201	93	31.4
*Procedure (p-value = 0.015 *)*				
LIMB - lower limb - total hip replacement	45 (4.2%)	41	4	8.9
LIMB - lower limb fracture - Cannulated Screws	5 (0.5%)	5	0	0.0
LIMB - lower limb fracture - Reduction and Internal Fixation	15 (1.4%)	10	5	33.3
LIMB - lower limb fracture - Dynamic Hip Screw	276 (26%)	195	81	29.3
LIMB - lower limb fracture - Reduction and Intramedullary Fixation	243 (22.9%)	169	73	30.2
LIMB - lower limb fracture - Partial Hip Replacement (Hemiarthroplasty)	479 (45.1%)	326	150	31.5
Missing	0 (0.0%)	0	0	0.0

Figure S1. Boxplots showing distributions of pre-surgery measures by outcome status (died or alive). Boxes show interquartile range (IQR), bars medians and whiskers are 1.5 times IQR.

For peer review only

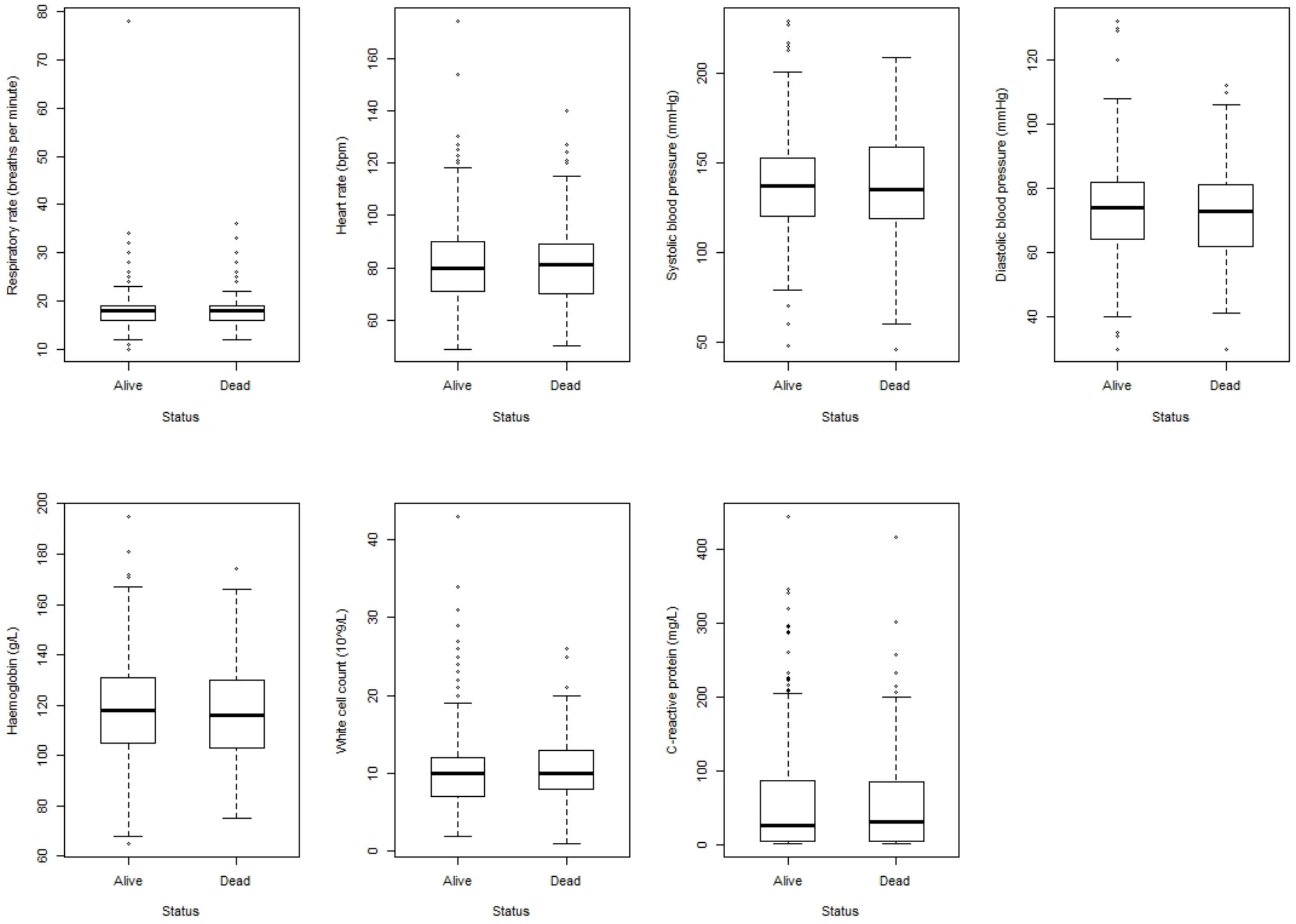
Respiratory rate (breaths per minute)
Heart rate (bpm)
Systolic blood pressure (mmHg)
Diastolic blood pressure (mmHg)
Haemoglobin (g/L)
White cell count (10^9/L)
C-reactive protein (mg/L)
Alive
Dead
Status

44

For peer review only

STROBE Statement—Checklist of items that should be included in reports of ***cohort studies***

	Item No	Recommendation	Page No
**Title and abstract**	1	(*a*) Indicate the study's design with a commonly used term in the title or the abstract<br>(*b*) Provide in the abstract an informative and balanced summary of what was done and what was found	1 & 2
**Introduction**			
Background/rationale	2	Explain the scientific background and rationale for the investigation being reported	3
Objectives	3	State specific objectives, including any prespecified hypotheses	4
**Methods**			
Study design	4	Present key elements of study design early in the paper	5
Setting	5	Describe the setting, locations, and relevant dates, including periods of recruitment, exposure, follow-up, and data collection	5
Participants	6	(*a*) Give the eligibility criteria, and the sources and methods of selection of participants. Describe methods of follow-up<br>(*b*) For matched studies, give matching criteria and number of exposed and unexposed	6
Variables	7	Clearly define all outcomes, exposures, predictors, potential confounders, and effect modifiers. Give diagnostic criteria, if applicable	7
Data sources/ measurement	8*	For each variable of interest, give sources of data and details of methods of assessment (measurement). Describe comparability of assessment methods if there is more than one group	6
Bias	9	Describe any efforts to address potential sources of bias	8
Study size	10	Explain how the study size was arrived at	N/A
Quantitative variables	11	Explain how quantitative variables were handled in the analyses. If applicable, describe which groupings were chosen and why	8
Statistical methods	12	(*a*) Describe all statistical methods, including those used to control for confounding<br>(*b*) Describe any methods used to examine subgroups and interactions<br>(*c*) Explain how missing data were addressed<br>(*d*) If applicable, explain how loss to follow-up was addressed<br>(*e*) Describe any sensitivity analyses	8
**Results**			
Participants	13*	(a) Report numbers of individuals at each stage of study—eg numbers potentially eligible, examined for eligibility, confirmed eligible, included in the study, completing follow-up, and analysed<br>(b) Give reasons for non-participation at each stage<br>(c) Consider use of a flow diagram	9
Descriptive data	14*	(a) Give characteristics of study participants (eg demographic, clinical, social) and information on exposures and potential confounders<br>(b) Indicate number of participants with missing data for each variable of interest<br>(c) Summarise follow-up time (eg, average and total amount)	9
Outcome data	15*	Report numbers of outcome events or summary measures over time	9

For peer review only

Main results	16	(*a*) Give unadjusted estimates and, if applicable, confounder-adjusted estimates and their precision (eg, 95% confidence interval). Make clear which confounders were adjusted for and why they were included<br>(*b*) Report category boundaries when continuous variables were categorized<br>(*c*) If relevant, consider translating estimates of relative risk into absolute risk for a meaningful time period	9-12
Other analyses	17	Report other analyses done—eg analyses of subgroups and interactions, and sensitivity analyses	9-12
**Discussion**			
Key results	18	Summarise key results with reference to study objectives	13
Limitations	19	Discuss limitations of the study, taking into account sources of potential bias or imprecision. Discuss both direction and magnitude of any potential bias	16
Interpretation	20	Give a cautious overall interpretation of results considering objectives, limitations, multiplicity of analyses, results from similar studies, and other relevant evidence	13-15
Generalisability	21	Discuss the generalisability (external validity) of the study results	13-15
**Other information**			
Funding	22	Give the source of funding and the role of the funders for the present study and, if applicable, for the original study on which the present article is based	1

*Give information separately for exposed and unexposed groups.

Note: An Explanation and Elaboration article discusses each checklist item and gives methodological background and published examples of transparent reporting. The STROBE checklist is best used in conjunction with this article (freely available on the Web sites of PLoS Medicine at http://www.plosmedicine.org/, Annals of Internal Medicine at http://www.annals.org/, and Epidemiology at http://www.epidem.com/). Information on the STROBE Initiative is available at http://www.strobe-statement.org.

For peer review only

For peer review only - http://bmjopen.bmj.com/site/about/guidelines.xhtml
